# Ultralarge Virtual Screening Identifies SARS-CoV-2 Main Protease Inhibitors with Broad-Spectrum Activity against Coronaviruses

**DOI:** 10.1021/jacs.1c08402

**Published:** 2022-02-10

**Authors:** Andreas Luttens, Hjalmar Gullberg, Eldar Abdurakhmanov, Duy Duc Vo, Dario Akaberi, Vladimir O. Talibov, Natalia Nekhotiaeva, Laura Vangeel, Steven De Jonghe, Dirk Jochmans, Janina Krambrich, Ali Tas, Bo Lundgren, Ylva Gravenfors, Alexander J. Craig, Yoseph Atilaw, Anja Sandström, Lindon W. K. Moodie, Åke Lundkvist, Martijn J. van Hemert, Johan Neyts, Johan Lennerstrand, Jan Kihlberg, Kristian Sandberg, U. Helena Danielson, Jens Carlsson

**Affiliations:** † Science for Life Laboratory, Department of Cell and Molecular Biology, Uppsala University, SE-75124 Uppsala, Sweden; § Science for Life Laboratory, Biochemical and Cellular Assay Facility, Drug Discovery and Development Platform, Department of Biochemistry and Biophysics, Stockholm University, Solna, SE-17121 Stockholm, Sweden; $ Science for Life Laboratory, Department of Chemistry-BMC, Uppsala University, SE-75123 Uppsala, Sweden; ∇ Department of Chemistry-BMC, Uppsala University, SE-75123 Uppsala, Sweden; ∥ Department of Medical Biochemistry and Microbiology, Zoonosis Science Center, Uppsala University, SE-75123 Uppsala, Sweden; ⊥ BioMax beamline, MAX IV Laboratory, Fotongatan 2, SE-22484 Lund, Sweden; # KU Leuven, Department of Microbiology, Immunology and Transplantation, Rega Institute, Laboratory of Virology and Chemotherapy, 3000 Leuven, Belgium; 7 Global Virus Network, Baltimore, Maryland 21201, United States; 8 Department of Medical Microbiology, Leiden University Medical Center, 2333ZA Leiden, The Netherlands; 9 Science for Life Laboratory, Drug Discovery & Development Platform, Department of Organic Chemistry, Stockholm University, Solna, SE-17121 Stockholm, Sweden; 10 Department of Medicinal Chemistry, Uppsala University, SE-75123 Uppsala, Sweden; 11 Uppsala Antibiotic Centre, Uppsala University, SE-75123 Uppsala, Sweden; 12 Department of Medical Sciences, Section of Clinical Microbiology, Uppsala University, SE-75185 Uppsala, Sweden; 13 Department of Physiology and Pharmacology, Karolinska Institutet, SE-17177 Stockholm, Sweden; 14 Science for Life Laboratory, Drug Discovery & Development Platform, Uppsala Biomedical Center, Uppsala University, SE-75123 Uppsala, Sweden

## Abstract

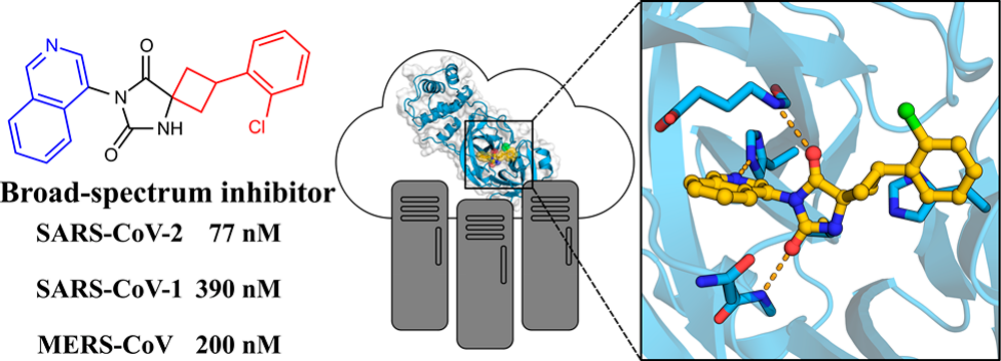

Drugs targeting SARS-CoV-2 could have saved millions of lives during the COVID-19 pandemic, and it is now crucial to develop inhibitors of coronavirus replication in preparation for future outbreaks. We explored two virtual screening strategies to find inhibitors of the SARS-CoV-2 main protease in ultralarge chemical libraries. First, structure-based docking was used to screen a diverse library of 235 million virtual compounds against the active site. One hundred top-ranked compounds were tested in binding and enzymatic assays. Second, a fragment discovered by crystallographic screening was optimized guided by docking of millions of elaborated molecules and experimental testing of 93 compounds. Three inhibitors were identified in the first library screen, and five of the selected fragment elaborations showed inhibitory effects. Crystal structures of target–inhibitor complexes confirmed docking predictions and guided hit-to-lead optimization, resulting in a noncovalent main protease inhibitor with nanomolar affinity, a promising in vitro pharmacokinetic profile, and broad-spectrum antiviral effect in infected cells.

## Introduction

The severe acute respiratory syndrome coronavirus 2 (SARS-CoV-2) has caused the greatest health crisis of this generation and already led to >5 million deaths worldwide.^1^ Despite promising vaccination programs against COVID-19, antiviral drugs will likely be crucial to control the inevitable future outbreaks of coronaviruses. Variants of SARS-CoV-2 for which the vaccines are less effective have already emerged, which is a strong indication that antiviral drugs are needed to complement vaccines in the long term.^2^ Analogous to common cold viruses, SARS-CoV-2 is expected to continue to circulate and remain a major threat to our society. In this scenario, antiviral agents are needed to treat patients that have been infected as well as be given prophylactically to protect high-risk groups. Although the road to development of a drug may be long, discovery of inhibitors targeting coronavirus replication must be prioritized as such therapeutic agents can improve the quality of life of millions of patients worldwide.

In early 2020, major global efforts were initiated to develop drugs to treat coronavirus infections. Attempts to repurpose approved drugs identified several promising candidates,^3^ but in larger clinical studies most of these compounds (e.g., remdesivir and hydroxychloroquine) had little or no effect on mortality or the duration of hospitalization.^4^ Among the proteins encoded by the SARS-CoV-2 genome, the main protease (M^pro^) has emerged as a promising target.^5^ Inhibition of M^pro^ blocks the processing of polyproteins produced by translation of the viral RNA, which is an essential step in SARS-CoV-2 replication. Targeting proteases has been a successful strategy for infections caused by the human immunodeficiency and hepatitis C viruses,^6^ but as M^pro^ is structurally and mechanistically different, new inhibitors need to be developed for coronaviruses. Prior to the COVID-19 pandemic, several compounds targeting M^pro^ of coronaviruses via covalent (e.g., **GC376**(7)) or noncovalent mechanisms (e.g., **ML188**(5,8,9)) had been identified. However, the noncovalent scaffolds were peptidomimetics, a chemotype that tends to have poor pharmacokinetic properties, and covalent modifiers typically require extensive optimization to modulate activity and selectivity.^10−12^ It was clear that development of safe and efficacious drugs targeting coronaviruses could benefit from the identification of novel noncovalent M^pro^ inhibitors with more favorable properties.

The size of commercial compound libraries is growing rapidly, and >10 billion make-on-demand molecules are currently available from chemical suppliers.^13^ These libraries provide opportunities to identify potential therapeutic agents that can readily be synthesized and tested for activity but require development of effective strategies for navigation in this enormous chemical space. The determination of high-resolution crystal structures of SARS-CoV-2 proteins^14^ has enabled virtual screening campaigns to identify hits that can be developed into antiviral drugs. Structure-based docking algorithms can sample and score binding poses in seconds, making it possible to evaluate large libraries, and this approach is not restricted to compounds that are physically available. As only a small set of top-scoring compounds are synthesized and tested, docking screens have the potential to substantially improve the efficiency of lead discovery.^15^ Encouragingly, molecular docking of chemical libraries also resulted in astonishingly high hit rates^16^ and identified novel chemotypes with high affinity.^17,18^ Although numerous virtual screens of large chemical libraries for SARS-CoV-2 inhibitors have recently been presented,^19,20^ only a small fraction of these have tested their predictions experimentally.^21,22^ Considering the high false-positive rate of docking, thorough validation of hits by several experimental methods is essential for progress toward developing drugs.^15^

We explored two different strategies to search for M^pro^ inhibitors in ultralarge chemical libraries using structure-based docking. In the first screen, a library with several hundred million diverse lead-like molecules was docked to the active site of M^pro^, and top-ranked compounds were selected for experimental evaluation. The second screen focused on optimization of a fragment identified in a crystallographic screen^23^ by creating a focused library with millions of compounds. Hits emerged from both sets of compounds, and structure-guided hit-to-lead optimization identified potent inhibitors with antiviral effect in cellular models. The most promising lead compounds were compared to previously identified M^pro^ inhibitors, including the clinical candidate **PF-07321332**.^24^ Comparison of the two screening approaches illuminates their utility as effective strategies to navigate in ultralarge chemical space for finding starting points for drug discovery.

## Results

### Docking Screen of an Ultralarge Library for M^pro^ Inhibitors

**Figure 1:**
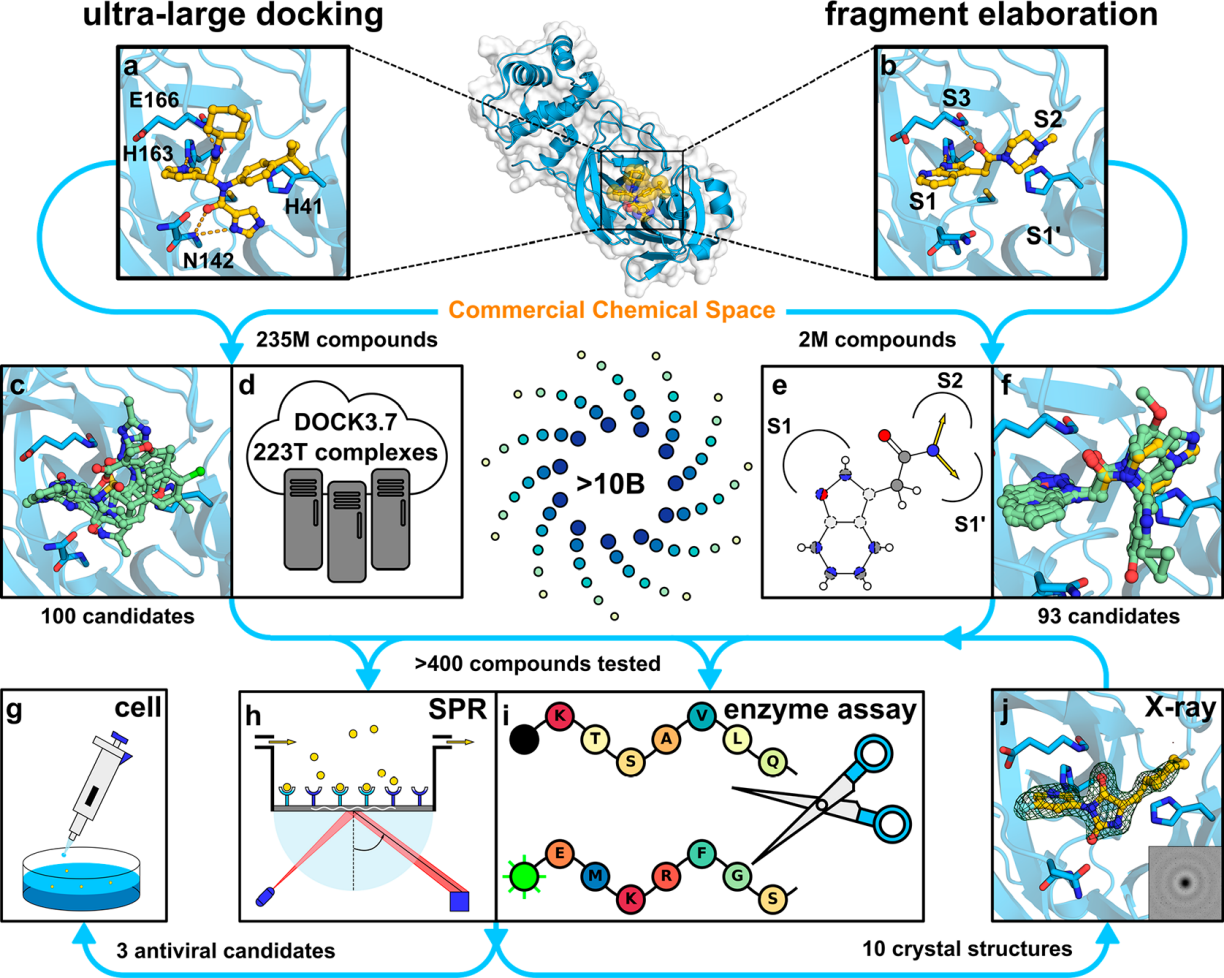
Overview of the two virtual screening approaches. (a) Crystal structure of M^pro^ in complex with inhibitor **X77** (PDB accession code: 6W63) was used in the ultralarge docking screen. (b) Crystal structure of M^pro^ in complex with a fragment (compound **4**, PDB accession code: 5RF7) was used in the docking screen of a focused library (fragment elaboration). (c, d) Compounds selected from molecular docking of a ZINC15 library containing 235 M lead-like molecules. (e, f) Compounds were selected from molecular docking of focused libraries with elaborated fragments. (g–j) Predicted inhibitors were evaluated in biophysical and biochemical assays, by crystallography, and in virus-infected cell models.

In the first virtual screen, 235 million compounds were docked to a crystal structure of M^pro^ determined in complex with the substrate-based inhibitor **X77** (PDB code: 6W63(25)). **X77** is an M^pro^ inhibitor that occupies all four major pockets (S1, S1′, S2, and S3) of the active site (Figure 1a). **X77** (racemic) was a suitable reference compound in our experimental assays, in which it had an affinity (*K*_D_) of 1.9 μM (Table 1) and a half maximal inhibitory concentration (IC_50_) of 2.8 μM (Supplementary Figure 1). The screened chemical library was composed of structurally diverse compounds with physicochemical properties characteristic of good starting points for drug discovery.^26^ The vast majority of the compounds originated from a commercial make-on-demand library and were hence novel chemical structures that had never been synthesized before. Each compound in the library was sampled in thousands of conformations in the M^pro^ active site, and a total of >223 trillion complexes were evaluated using the DOCK3.7 scoring function^27^ (Figure 1d) The screen required 83261 core hours (corresponding to 10 CPU years) and was performed in approximately 1 day on 3500 cores. The 300000 top-ranked compounds, corresponding to 0.12% of the library, were then clustered by topological similarity to identify a diverse set of candidate molecules. From the 5000 top-ranking clusters, 82 compounds were selected for experimental evaluation based on visual inspection of their complementarity to the active site. An additional set of 18 molecules was selected among the 3000 top-ranked compounds that formed the same set of hydrogen bonds as **X77**, i.e., to His163, Glu166, and Gly143. In the compound selection step, we also took into account contributions to ligand binding that are not included in the docking scoring function. Examples of reasons to exclude compounds from experimental testing are ligand strain, unsatisfied polar atoms of binding site residues or the compound, and unlikely tautomeric/ionization states.^28^

**Table 1:**
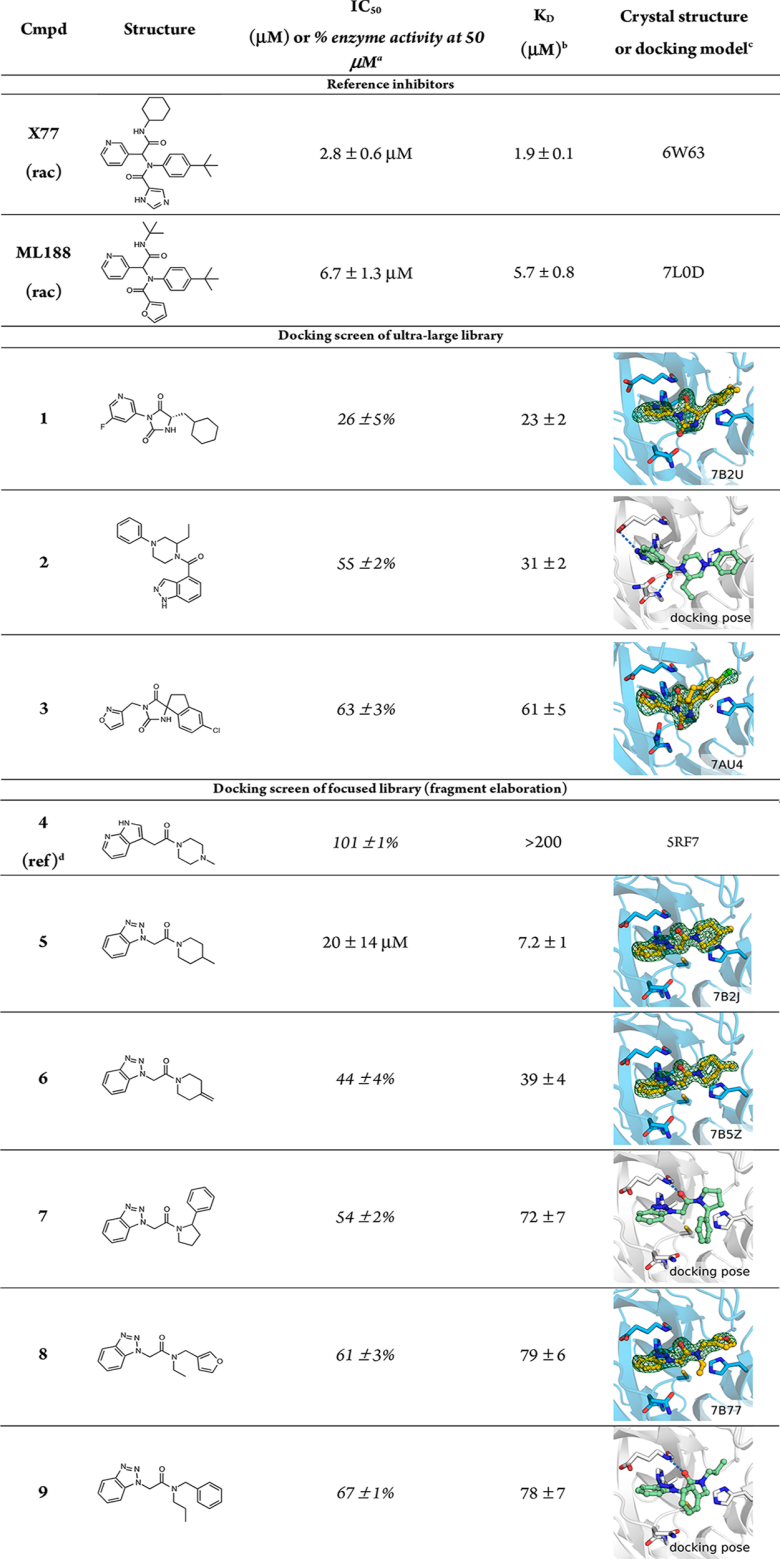
Hits from the Virtual Screens, Inhibitory Potencies, Equilibrium Constants, and Structures of Complexes with M^pro^

a Percent enzyme activity is expressed as mean ± SEM from two independent experiments. IC_50_ values are expressed as mean ± SD of 2–3 independent experiments (Supplementary Figure 1).

b Uncertainties are reported as standard deviations (*n* = 1). *K*_D_ values were determined by SPR biosensor analysis. The sensorgrams are shown in Supplementary Figure 2.

c Crystal structures with (*F*_o_ – *F*_c_) electron density difference maps (green isomesh) at +3σ carved at 1.5 Å from the ligand are shown in blue and docking models in gray. M^pro^ is shown as a cartoon. Selected side chains and the inhibitors are shown as sticks.

d Fragment identified by crystallographic screening.^23^

**Figure 2:**
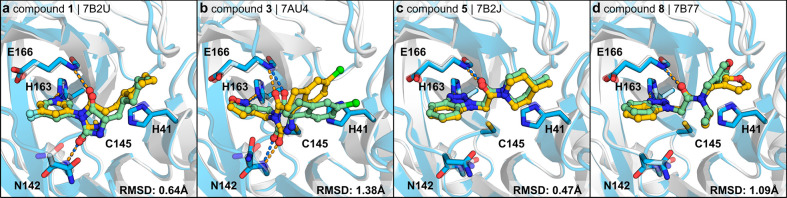
Confirmation of predicted binding modes by high-resolution crystal structures. The complexes predicted by docking (PDB accession codes of the protein structures: 6W63 for (a, b) and 5RF7 for (c, d); protein and inhibitors are shown as gray cartoons and green sticks, respectively) are shown together with solved crystal structures (protein and inhibitors are shown in blue and yellow, respectively). Selected side chains are shown as sticks, and hydrogen bonds are shown as dashed lines.

The 100 selected compounds (Figure 1c, Supplementary Data file 1) were tested in an M^pro^ enzyme inhibition assay (Figure 1i) and a direct binding interaction assay (Figure 1h) using a surface plasmon resonance (SPR) based biosensor at three concentrations (5, 15–20 and 50 μM). Nineteen compounds showed measurable and dose-dependent binding in the SPR biosensor screen, and three of these (compounds **1**–**3**) were also found to inhibit M^pro^ in the enzyme inhibition assay (<70% activity at 50 μM, Table 1, Supplementary Data file 1). For the three hits that were confirmed in both assays, the *K*_D_ values ranged from 23 to 61 μM and the enzyme activity was reduced to 26–63% at the highest tested concentration (50 μM). In agreement with the low affinities estimated for **2** and **3** by the SPR biosensor assay (Supplementary Figure 2), these compounds only showed inhibitory effects at 50 μM in the enzyme activity assay. Compound **1** had the highest inhibitory potency with an IC_50_ value of approximately 40 μM. The *K*_D_ determined by the SPR biosensor assay was 23 μM, corresponding to a good ligand efficiency of 0.30 kcal mol^–1^ heavy atom^–1^. Crystal structures of compounds **1** and **3** in complex with M^pro^ were determined at 1.6 and 1.8 Å resolution, respectively (Figure 1j, Supplementary Table 1). The crystallographic binding modes of the two inhibitors, which were based on a hydantoin scaffold, agreed remarkably well with the predicted complexes obtained by molecular docking with root-mean-square deviations (RMSDs) of 0.6 and 1.4 Å, respectively (Figure 2a,b). The hydantoin carbonyl groups formed hydrogen bonds to the backbone of residues Gly143 and Glu166, and substituents on the hydantoin core extended into the S2 and S1 pockets. Based on the docking scores, compound **1** was ranked in the top 0.002% of the chemical library, and the pyridinyl-hydantoin scaffold was strongly enriched by the virtual screen. Compound **2** was also among the high-ranking molecules and composed of a phenylpiperazine scaffold connected to an indazole-4-carbonyl group, which were predicted to extend into the S2 and S1 pockets, respectively (Table 1).

### Fragment-Guided Virtual Screening for M^pro^ Inhibitors

The second virtual screen was initiated based on a hit from a crystallographic fragment screen performed at the Diamond Light Source.^23^ One of the 24 identified active site bound fragments (compound **4**) was selected as a starting point for further elaboration (Figure 1b). In the crystal structure (PDB code: 5RF7), compound **4** occupied the S1 and S2 pockets but did not extend into S1′ or S3 in contrast to the larger inhibitor **X77**. The fragment showed superstoichiometric binding to M^pro^ in the SPR biosensor assay (*K*_D_ > 200 μM) and had no effect on enzyme activity at 50 μM (Table 1). Fragment-to-lead optimization was guided by searches in a library with >10 billion make-on-demand compounds combined with docking screens to select the best candidates. Based on visual analysis of the complex, we designed chemical patterns that could encompass the key features of the fragment’s polar interactions and also had the potential to place growth vectors into the S1′ and S2 pockets (Figure 1e,f). A five-membered aromatic heterocyclic ring and an amide that formed hydrogen bonds to His163 and Glu166, respectively, were considered to be crucial for the scaffold, but the size and topology of the heterocycle in the S1 pocket were allowed to vary. We also searched for both branched and tethered amides with potential to grow into the S2 pocket. More than two million elaborations in the make-on-demand library matched these patterns and were subsequently docked to the active site. Of these, ∼5000 compounds maintained the core-binding mode with favorable docking scores. These complexes were visually inspected, and 93 compounds were selected for experimental evaluation (Supplementary Data file 1). The selected compounds explored diverse heterocyclic rings to optimize hydrogen-bonding interactions with the S1 pocket and aliphatic as well as aromatic chemical groups in the S2 and S1′ pocket.

Twenty-one elaborated fragments showed dose-dependent binding in the SPR biosensor experiments, and five of these also inhibited M^pro^ activity at 50 μM (<70% activity, compounds **5**–**9**, Table 1 and Supplementary Data file 1). *K*_D_ values determined by SPR biosensor measurements ranged from 7.2 to 79 μM for the five hits confirmed in both assays (Supplementary Figure 2). Compound **5** had an IC_50_ of 20 μM in the enzyme assay and a *K*_D_ of 7.2 μM in the SPR assay (Supplementary Figure 2), corresponding to an excellent ligand efficiency of 0.37 kcal mol^–1^ heavy atom^–1^. Crystal structures of complexes were successfully determined for compounds **5**, **6**, and **8** with resolutions of 1.6 Å in all three cases (Supplementary Table 1). The crystallographic binding modes confirmed the docking predictions with RMSDs of 1.3, 1.5, and 1.4 Å, respectively (Figure 2c,d).

### Structure-Guided Inhibitor Optimization

Hit-to-lead optimization was pursued for three scaffolds identified from the virtual screens. Compound design was guided by docking predictions and determined crystal structures. Rapid design-make-test cycles were enabled by combining commercial make-on-demand compounds with in-house synthesis, which led to the discovery of nanomolar M^pro^ inhibitors in less than 4 months.

**Figure 3:**
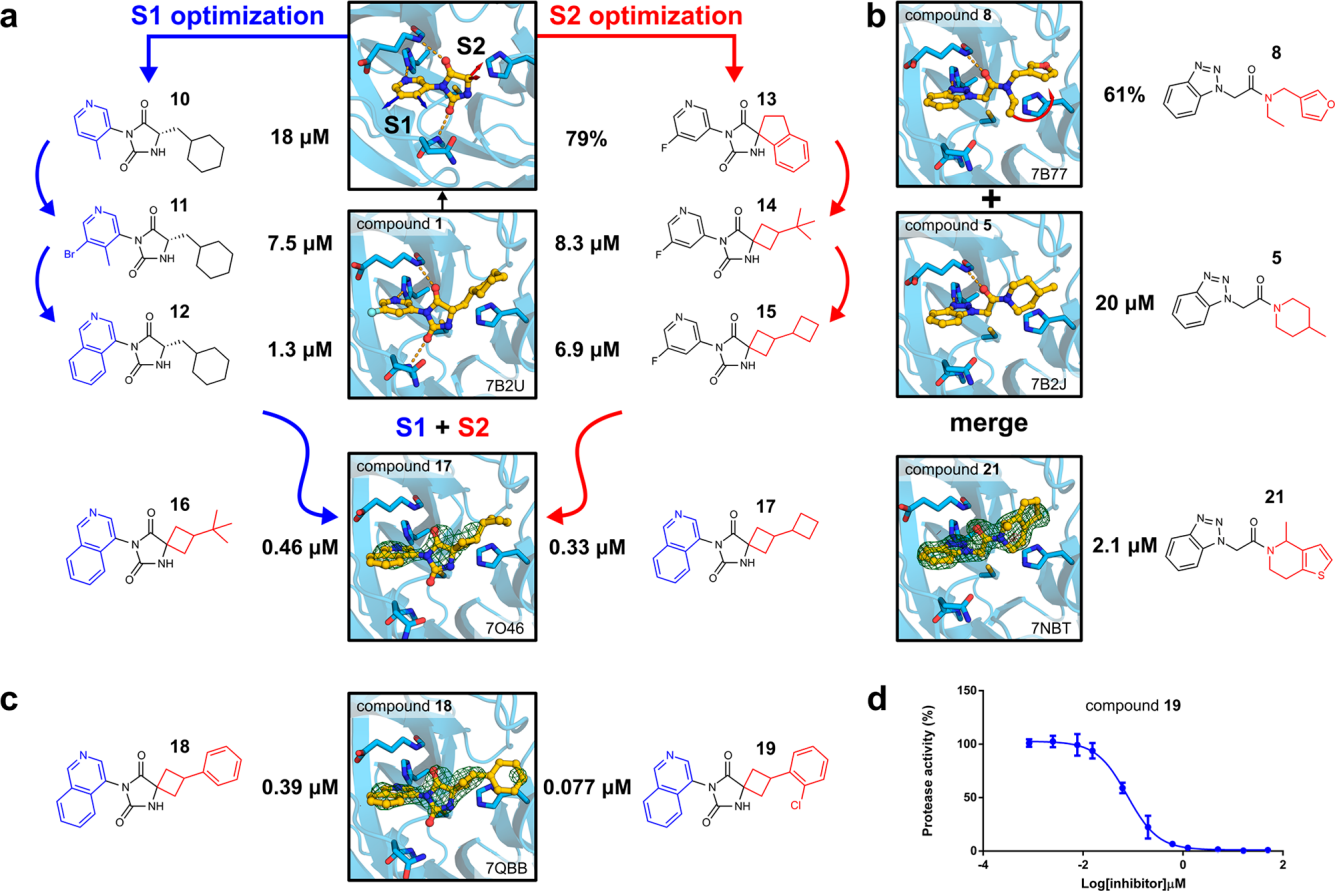
Overview of hit-to-lead optimization. (a) Docking hit confirmed by crystallography revealed two potential growing vectors (toward the S1 and S2 pockets) that were explored in parallel. Crystal structures of M^pro^ in complex with compounds **1** and **17** are shown. (b) Crystal structures of two docking hits inspired a fragment merging, yielding the more potent inhibitor **21**. Crystal structures of M^pro^ in complex with compounds **5** and **21** are shown. (c) Crystal structure of M^pro^ in complex with compound **18** led to the design of the potent inhibitor **19**. (d) M^pro^ activity assay of compound **19**. Data points represent mean ± SD from three independent experiments. IC_50_ values are expressed as means of three to four independent experiments. Percentage enzyme activity values are expressed as means of two independent experiments. Electron density difference maps (*F*_o_ – *F*_c_) at +3σ carved at 1.5 Å from ligands are shown as green isomeshes.

Hit optimization of the scaffold represented by compounds **1** and **3** was guided by crystal structures of these inhibitors bound to M^pro^ (Figure 3a). As their common hydantoin scaffold showed excellent hydrogen-bonding complementarity to the active site, this chemotype was maintained. The commercial make-on-demand library contained 43 million hydantoins, and we performed searches among these to identify promising candidates for experimental evaluation. Variations in the S1 and S2 pockets were explored systematically in several cycles to identify the optimal combination of substituents. Chemical pattern matching based on these two growth vectors identified >200000 possible elaborations. In total, 137 hydantoin-based analogues were experimentally evaluated (Supplementary Data file 1). In the S1 pocket, pyridine-based moieties based on compound **1** resulted in the greatest increase of potency (Figure 3a). Guided by structural information, substitutions at positions adjacent to the aromatic nitrogen were excluded because these were likely to clash with the protein surface. Instead, efforts were focused on the growth vector extending toward Asn142 in the crystal structure, which was also supported by the bulkier benzotriazole scaffold of compound **5** occupying S1 and by merging fragments identified by crystallographic screening.^23^ Pyridine elaborations of increasing size gradually improved the potency of derivatives of compound **1** from a weak effect at 50 μM to IC_50_ values of 18, 7.5, and 1.3 μM for compounds **10**, **11**, and **12**, respectively. The isoquinoline moiety, which was also identified as a promising elongation of the pyridine by the COVID Moonshot Consortium,^29^ resulted in the highest potency in this series. In the S2 pocket, the best substituents (compounds **14** and **15**) were identified from spirocyclic analogues of compound **3**. Combining the spiro-indanyl moiety of compound **3** with a pyridine in the S1 pocket (compound **13**) did not improve the potency compared to the virtual screening hit, but the predicted binding mode of this inhibitor was supported by crystallography (Supplementary Figure 3). Several attempts were then made to incorporate a tethered motif that would rigidify the alkyl substituent of compound **1** and increase complementarity to the S2 pocket. Spiro-cyclobutyl substitutions in compounds **14** and **15** improved IC_50_ values to 8.3 and 6.9 μM, respectively, and the SPR biosensor measurements confirmed that compound **15** interacted with M^pro^ (*K*_D_ = 6.6 μM, Supplementary Figure 2). The optimal substituents in the S1 and S2 pockets were integrated into a single chemical series by in-house synthesis, resulting in a synergistic improvement of inhibitor potency and affinity. Compounds **16** and **17** had IC_50_ values of 0.46 and 0.33 μM, respectively (Supplementary Figure 1), and also showed high affinities for M^pro^ (*K*_D_ values of 0.14 and 0.15 μM, respectively, Supplementary Figure 2). A crystal structure (2.2 Å resolution) supported the predicted interactions of the isoquinoline and hydantoin rings of compound **17** (Figure 3). The stereoisomeric mixtures of compounds **16** and **17** were separated and their structures were determined using NOESY experiments as compounds **16a**, **16b**, **17a**, and **17b** (Supplementary Figure 9). Of these, **16a** and **17b** showed the highest inhibitory potencies with IC_50_ values of 0.24 and 0.35 μM (Supplementary Table 4). As further elaboration of the aliphatic tails of compounds **16** and **17** was synthetically challenging, we introduced a phenyl ring at this position, and this inhibitor displayed similar activity (IC_50_ = 0.39 μM and *K*_D_ = 0.17 μM). A crystal structure of M^pro^ in complex with compound **18** (2.0 Å resolution, Figure 3c) was obtained and guided further optimization. Addition of an *o*-chloro substituent to the phenyl resulted in compound **19**, which showed improved potency (IC_50_ = 0.077 μM, Figure 3c,d) and affinity (*K*_D_ = 0.038 μM, Supplementary Figure 2). In the same enzyme inhibition assay, **GC376** and **PF-07321332** had potencies of 0.073 and 0.033 μM, respectively. The potency of the latter compound may be misleading considering that it has a covalent mechanism with a reversibility that may not be accounted for in this steady-state-based assay. Compared to the virtual screening hit (compound **1**), **19** had >600-fold higher affinity and an improved ligand efficiency (0.37 kcal mol^–1^ heavy atom^–1^ for the optimized compounds and 0.30 kcal mol^–1^ heavy atom^–1^ for compound **1**, calculated from *K*_D_ values) with physicochemical properties characteristic of promising leads for drug discovery (Supplementary Table 2).^30,31^

Using the same strategy as for compounds **1** and **3**, improvement of the phenylpiperazine scaffold represented by compound **2** involved testing of 67 compounds (Supplementary Data file 1). In this case, optimization of interactions in the S2 pocket was guided by the docking model of compound **2**. Decorating the aromatic ring in the S2 pocket with small substituents yielded the micromolar inhibitor **20** (IC_50_ = 7.2 μM, Supplementary Figures 1 and 4).

The benzotriazole scaffold identified by fragment elaboration was further optimized by fine-tuning interactions in the S2 pocket, and the most successful strategy was to integrate information from several crystal structures combined with molecular docking of commercial compounds (Figure 3b). Elaborated compounds (Supplementary Data file 1) were identified by an iterative chemical pattern searching among >10 billion make-on-demand molecules. By combining features of compounds **5** and **8**, the improved inhibitor **21** (IC_50_ = 2.1 μM) was identified after evaluation of 79 commercially available compounds, and crystal structures confirmed the predicted interactions in the S2 pocket (Figure 3b).

For compounds **20** and **21**, the *K*_D_ values determined by the SPR biosensor assay were 4.7 and 2.3 μM, respectively (Supplementary Figure 2). These two scaffolds have good physicochemical properties and ligand efficiencies (0.29 and 0.35 kcal mol^–1^ heavy atom^–1^ for **20** and **21**, respectively) and, hence, represent favorable starting points for further optimization (Supplementary Table 2).

### Counter Screens

To assess if inhibition involved covalent modification of cysteine residues, we performed assays in the presence of the reducing agent dithiothreitol (DTT) for several of the discovered compounds (Supplementary Table 3, Supplementary Figure 5). Addition of DTT ensures that accessible cysteines are in their reduced form and will indicate if inhibitors act via a false mechanism of redox-cycling.^32^ The IC_50_ values of compounds **16**–**21** were not altered by the addition of DTT. Additional enzymatic assays for M^pro^ were performed in the presence of detergent to control for promiscuous inhibition due to colloidal aggregation.^33^ IC_50_ values were not sensitive to the presence of Triton X-100, supporting that the observed inhibition was not due to colloidal aggregation. Selectivity was assessed by testing the inhibitors against human cathepsin S. Compounds **16**–**21** did not show any effect on cathepsin S activity (IC_50_ > 50 μM, Supplementary Figure 5), but the peptidomimetics **GC376** and **PF-07321332** inhibited this off-target (IC_50_ = 0.002 and 5.7 μM respectively). To further assess the selectivity profile of our scaffold, compound **19** was tested against a panel of nine other human proteases. Compound **19** showed no significant inhibitory effect against these antitargets at a concentration of 10 μM (Supplementary Table 5). Together with SPR biosensor assays, these controls indicated that the discovered compounds were noncovalent and selective M^pro^ inhibitors (Supplementary Figure 5, Supplementary Table 5).

### Antiviral Effect in Cell Assays and In Vitro Pharmacokinetic Profiling

**Figure 4:**
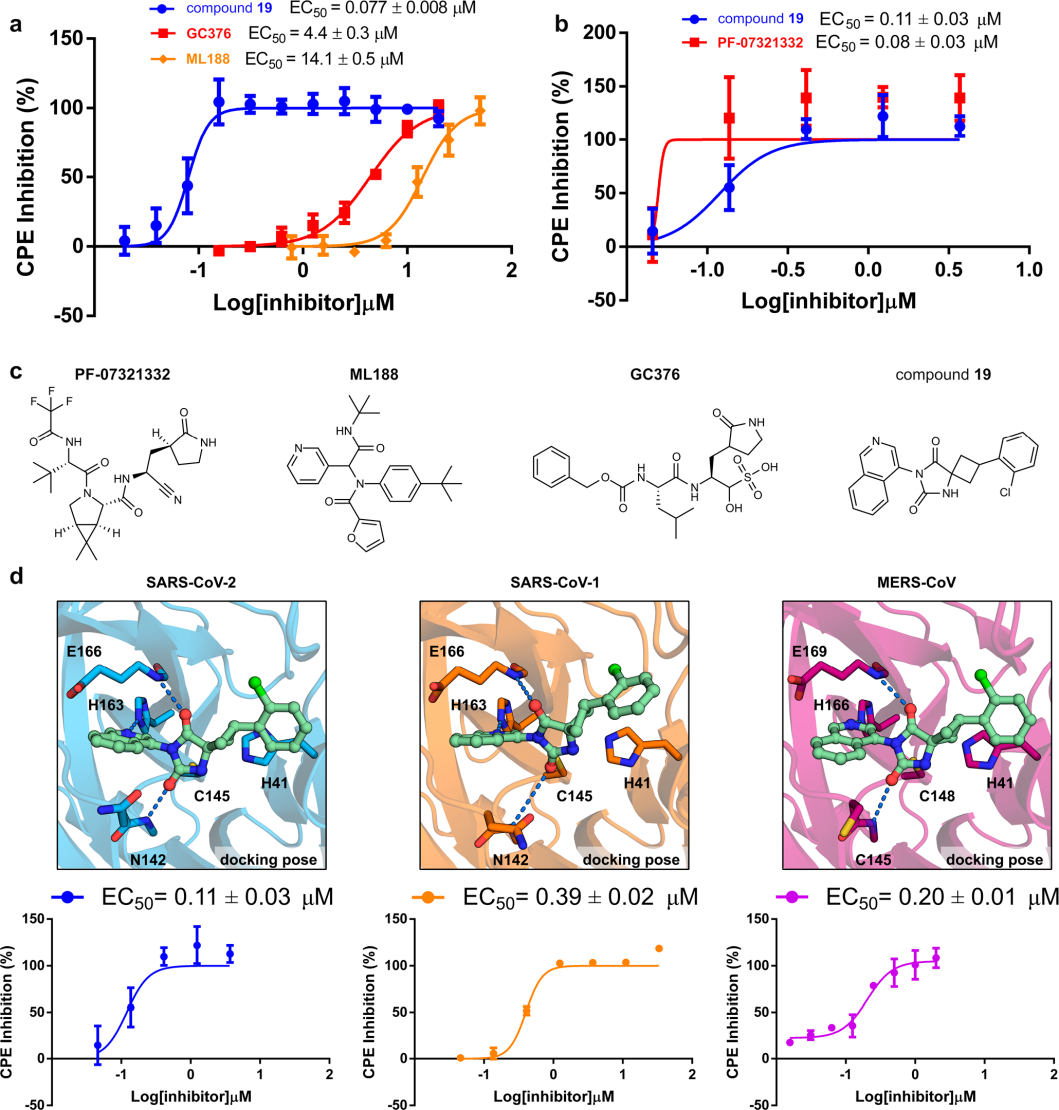
Overview of cell assays of compound **19** and reference inhibitors. (a) Inhibitory effect of compounds **19**, **GC376**, and **ML188** (rac) on CPE induced by SARS-CoV-2 infection in Vero E6 cells 72 h post infection. Assay was performed with a 3 h preincubation step for **GC376** and **ML188**. EC_50_ values are expressed as mean ± SEM from at least two independent experiments. (b) Inhibitory effect of compound **19** and **PF-07321332** on CPE induced by SARS-CoV-2 infection in Huh7 cells (without preincubation). EC_50_ values are expressed as mean ± SEM from two independent experiments. (c) Compounds tested in cellular assays. (d) Predicted binding modes of compound **19** in the active sites of M^pro^ from SARS-CoV-2 (blue), SARS-CoV-1 (orange), and MERS-CoV (purple) are shown. Protein–ligand hydrogen bonds are shown as dashed lines. CPE developed in infected Huh7 cells (SARS-CoV-2 and MERS-CoV) and Vero E6 cells (SARS-CoV-1) treated with compound **19**. EC_50_ values are expressed as mean ± SEM from two independent experiments.

The antiviral effect of compounds **16**, **17**, and **19** was evaluated in SARS-CoV-2-infected cells (Figure 1g). The three compounds showed dose-dependent inhibition of the cytopathic effect (CPE) (Figure 4a, Supplementary Figure 6) with EC_50_ values of 1.7, 1.6, and 0.077 μM and no significant cytotoxicity at the highest tested concentration in Vero E6 cells (50% cytotoxicity concentration, CC_50_ > 20 μM, Supplementary Table 6). The antiviral activity was also confirmed in a yield reduction assay that assessed the inhibitory activity of the compounds on the viral replication using RT-qPCR. In these experiments, compounds **16**, **17**, and **19** inhibited SARS-CoV-2 viral replication with EC_50_ values of 1.0, 0.9, and 0.044 μM, respectively (Supplementary Figure 7). The previously reported M^pro^ inhibitors **GC376**, **ML188**, and **X77** were also tested in the CPE-based assay for comparison (Figure 4c). The covalent peptidomimetic **GC376** and noncovalent inhibitors **ML188** and **X77** (rac) showed antiviral effect (EC_50_ = 4.4, 14.1, and 34.3 μM, respectively, in Vero E6 cells), but their potencies were lower than for **16**, **17**, and **19**. Pfizer’s clinical candidate (**PF-07321332)** and compound **19** were compared in CPE-based assays performed in Huh7 cells. These M^pro^ inhibitors were found to have comparable potencies, with EC_50_ values of 0.08 and 0.11 μM, respectively (Figure 4b).

To further assess the potential of compound **19** as a starting point for development of broad-spectrum coronavirus drugs, we computationally docked this inhibitor to homology models of 29 reported active site mutants of M^pro^ and crystal structures of SARS-CoV-1 and MERS-CoV variants.^34−36^ The binding mode of compound **19** was maintained in all of the predicted complexes (Figure 4d, Supplementary Table 7), which indicated that our inhibitor could be active against other coronaviruses. In agreement with our computational modeling, compound **19** also inhibited the cytopathic effect of SARS-CoV-1 (EC_50_ = 0.39 μM, Vero E6 cells) and MERS-CoV (EC_50_ = 0.20 μM, Huh7 cells) (Figure 4d).

The in vitro ADME properties of compound **19** were promising with good metabolic stability in the presence of human liver microsomes (intrinsic clearance CL_int_ = 22 μL/min/mg) and plasma protein binding in human plasma (fraction unbound fu = 3.3%). In agreement with the potent antiviral effect in cells, compound **19** was highly permeable in a Caco-2 cell assay (*P*_app_ AB = 5.9 × 10^–5^ cm/s and *P*_app_ BA = 5.0 × 10^–5^ cm/s) with no observed efflux effect (efflux ratio of 0.8).

## Discussion

Three main findings emerged from our virtual screens of ultralarge chemical libraries for SARS-CoV-2 inhibitors. First, molecular docking of diverse and focused screening libraries identified eight M^pro^ inhibitors, and the predicted binding modes were confirmed by crystallography for five of these. Second, efficient hit-to-lead optimization was enabled by crystal structures and searches among billions of virtual compounds, resulting in three series of noncovalent inhibitors. Finally, the most promising leads have nanomolar IC_50_ values in an M^pro^ enzymatic assay, good in vitro ADME properties, and showed a potent antiviral effect against several coronaviruses in cellular assays.

The billions of compounds that are available in commercial libraries provide novel avenues for drug discovery, but navigating this vast chemical space to find leads is challenging. We compared two strategies to exploit ultralarge chemical libraries using docking screens. M^pro^ inhibitors were discovered by docking of an unbiased library with several hundred million molecules and a focused library based on a fragment. More diverse scaffolds were accessible in the first screen, but we anticipated that higher hit rates and potencies would be obtained from the focused library as the binding mode of the core fragment had been validated by crystallography.^23^ Encouragingly, both virtual screens delivered novel and nonoverlapping sets of inhibitors. However, to our surprise, the hit rates of the two strategies (i.e., the percentage of active compounds among those tested experimentally) were similar, as shown by validation both in the enzyme activity assay (3% and 5%) and the more sensitive SPR biosensor screen (19% and 21%). Although the most potent hit was discovered by the fragment elaboration (compound **5**, IC_50_ = 20 μM), compound **1** from the diverse library only had a 2-fold higher IC_50_ value (∼40 μM). These observations could reflect that docking scoring functions may be more proficient in ranking diverse chemotypes rather than differentiating between closely related elaborations of the same scaffold.^37^ Our results suggest that docking of large and unbiased libraries should be prioritized over focused subsets because more diverse leads will be identified with a marginal loss of hit rates and activities.

The commercial libraries also facilitated hit-to-lead optimization. Optimization was primarily driven by searches among >10 billion make-on-demand compounds, which enabled rapid design-make-test cycles. Remarkably, millions of analogues with diverse substituents were available for each scaffold in the first iterations. These molecules had generally not been previously synthesized, but as they are made with well-characterized reactions and available building blocks, the chemical supplier was able to deliver optimized inhibitors within a few weeks. In only a few iterations, three scaffolds were optimized from ∼20–40 μM to single-digit micromolar potencies. However, as the size and molecular complexity of the compounds increased, the number of analogues in the make-on-demand library decreased and designs based on the active site structure were not available. As we approached the outer edge of commercial chemical space, in-house synthesis proved essential to obtain the leads **16**, **17**, and **19**. These compounds belong to the small set of noncovalent and nonpeptidomimetic inhibitors of M^pro^ with nanomolar inhibitory activities.^29,38,39^ Compared to the noncovalent inhibitors that were available when the virtual screen was performed (**ML188** and **X77**), compound **19** has a better ligand efficiency and physicochemical properties and an antiviral activity that is 2 orders of magnitude more potent. Subsequent to our discovery of the hydantoin-based M^pro^ inhibitors, the COVID Moonshot Consortium also explored this moiety in their lead series.^29^ These results illustrate the ability of different structure-guided design approaches to identify privileged scaffolds that can rapidly be developed to potent inhibitors.^29,38^ Our study represents one of the few prospective docking campaigns of several hundred million compounds^17,18,40^ and further supports how access to make-on-demand libraries can expedite drug discovery. The initial hit (compound **1**) had good ligand efficiency and low lipophilicity, which was an excellent starting point for optimization. Only six additional heavy atoms were added in optimization of the hydantoin scaffold, leading to >600-fold increase of affinity and an improved ligand efficiency, an unusual achievement in hit-to-lead optimization.

The clinical candidate from Pfizer (**PF-07321332**) is the most advanced effort to develop an M^pro^ inhibitor to treat SARS-CoV-2 infections.^24^ In our enzyme inhibition assays, compound **19** and **PF-07321332** both have nanomolar potencies. Encouragingly, compound **19** did not inhibit any human proteases considered to be potential off-targets, whereas **PF-07321332** also inhibits human cathepsin S, indicating that promiscuity may be a liability of this compound class. Moreover, compound **19** showed comparable efficacy as **PF-07321332** against SARS-CoV-2 and also displayed antiviral effect in SARS-CoV-1 and MERS-CoV infected cells. Based on the broad-spectrum antiviral effect combined with promising selectivity and in vitro pharmacokinetic profile, the scaffold represented by **19** is one of the most promising for development of an antiviral drug targeting SARS-CoV-2.

## Materials and Methods

### Molecular Docking

A crystal structure (PDB accession code: 6W63(25)) of the SARS-CoV-2 M^pro^ bound to an inhibitor (**X77**) was used in the large-scale docking screen. Crystallographic waters and other solvent molecules were first removed from the structure. The atoms of **X77** were used to define the binding site by generating 45 matching spheres. DOCK3.7^27^ uses a flexible ligand algorithm that superimposes rigid segments of a molecule’s precalculated conformational ensemble on top of the matching spheres. A total of 19 additional matching spheres were added to enhance orientational sampling in the binding site. Histidine protonation states were assigned manually based on visual inspection of hydrogen bonding networks. For example, the key residue His163 was protonated at the Nε atom because of the hydrogen-bond interaction with the pyridine of **X77** (acceptor) and hydroxyl group of Tyr161 (donor). Histidines 41, 163, 164, 172, and 246 were protonated at the Nε, whereas histidines 64 and 80 were protonated at the Nδ. The remainder of the enzyme structure was protonated by REDUCE^41^ and assigned AMBER^42^ united atom charges. The dipole moments of three residues involved in recognition of **X77** were increased to favor interactions with these. This technique is common practice for users of DOCK3.7 to improve docking performance^43^ and has been used in previous virtual screens.^17,18,22,44,45^ For residue His163, the partial atomic charges of the Nε and Hε atoms were increased without changing the net charge of the residue. The dipole moment of the backbone amide of residues Asn142 and Glu166 was also increased using the same technique. The atoms of the cocrystallized inhibitor were combined with atoms of bound noncovalent fragments from other crystal structures^23^ to create two sets of sphere layers on the protein surface (referred to as thin spheres). One set of thin spheres with radius 1.2 Å described the low protein dielectric and defines the boundary between solute and solvent. A second set of thin spheres with radius 0.3 Å was used to calibrate ligand desolvation penalties. Scoring grids were precalculated using QNIFFT^46^ for Poisson–Boltzmann electrostatic energies, SOLVMAP^47^ for ligand desolvation energies, and CHEMGRID^48^ for AMBER van der Waals energies. The crystal structure (PDB accession code: 5RF7(23)) of M^pro^ bound to compound **4** was used for fragment elaboration docking calculations. Scoring grids were generated using the same protocol as described above, and the dipole moments of residues His163 and Glu166 were increased in this case. Property-matched and property-perturbed decoys^49,50^ of noncovalent bound fragments identified by crystallography^23^ were generated using in-house scripts. The obtained control sets were used to evaluate the performance of the docking grids by means of ligands-over-decoys enrichments. Enrichment of ligands and the predicted binding poses of the fragments were used to select the final grid parameters.

In the screen of the ultralarge library, DOCK3.7 was used to dock the lead-like subset of ZINC15^51^ (http://zinc15.docking.org) to the M^pro^ active site. This subset is characterized by good physicochemical properties for screening assays (cLogP ≤ 3.5 and molecular weight ≤350 Da). The library contained more than 235 M commercially available molecules, of which 228 M molecules were successfully docked. For each compound, 5551 orientations were calculated on average, and for each orientation, an average of 475 conformations was sampled. For each ligand, the best scoring pose was optimized using a simplex rigid-body minimizer. Top-scoring molecules were filtered using a PAINS-filter to reduce the risk of encountering false positives.^52^ The diversity among the top-ranked compounds was increased by clustering the 300000 best scoring molecules using ECFP4-based fingerprints and a Tanimoto coefficient of 0.5.^53^ The best scoring member of the cluster was chosen as a representative. Out of the 33876 resulting clusters, the 5000 top-scoring were visually inspected. In addition, the top 3000 nonclustered molecules that formed hydrogen bonds with residues His163, Glu166, and Gly143 were also visually inspected. In the screens of the >2 million fragment elaborations (based on compound **4**), the 50 lowest energy poses of each molecule were retained from the docking. The lowest energy pose that had an RMSD value below 2 Å from compound **4** was considered as the most relevant pose. Using an in-house script, the RMSD value was calculated between atoms that fulfilled the SMARTS definition, allowing for the exchange of heteroatoms in the core scaffold.

Crystal structures of the SARS-CoV-1 (PDB accession code: 7LMG) and MERS-CoV (PDB accession code: 4YLU) M^pro^ bound to inhibitors were prepared for molecular docking using the same protocols as for the SARS-CoV-2 M^pro^ structures.^35,36^ The atoms of the cocrystallized inhibitors were used to generate 45 matching spheres in the active sites. For SARS-CoV-1, dipole moments of Asn142, His163, and Glu166 were increased with the same protocol as the SARS-CoV-2 model. For MERS-CoV, the dipole moments of Cys145, His166, and Glu169 were increased.

### Cheminformatics and Preparation of Chemical Libraries

Chemical SMARTS patterns were constructed using structural information and an interactive SMARTS visualizer (https://smarts.plus).^54^ Chemical pattern matching was performed with OpenEye’s OEToolkits and Enamine’s November 2019 REAL space library (12.3 billion compounds). Matching molecules were filtered by a PAINS-filter to reduce the risk of having false positives.^52^ Molecules were prepared for docking using DOCK3.7 protocols (db2 format). Conformational ensembles were capped at 400 conformations per rigid segment and an interconformer RMSD diversity threshold of 0.25 Å.

### Modeling of SARS-CoV-2 M^pro^ Mutants

Mutation data were obtained from the GISAID SARS-CoV-2 database.^34^ A total of 841 unique mutant protein sequences were aligned to the reference sequence. For each sequence, a ligand-bound homology model was constructed using MODELER with a SARS-CoV-2 crystal structure (PDB code: 6W63) as template.^55^ Molecular docking grids were prepared using the same protocols as for the M^pro^ crystal structures. Symmetry-corrected ligand RMSD values were calculated after aligning the homology model to a reference crystal structure (PDB code: 7QBB).

### Expression and Purification of M^pro^ for Protease Activity Assays and Crystallography

SARS-CoV-2 M^pro^ was produced adopting a previously described construct^67^ containing nucleotide sequences corresponding to residues S1-Q306 (Chinese isolate, NCBI accession no. YP_009725301). In this construct, M^pro^ is flanked by an N-terminal GST (glutathione S-transferase) tag followed by a M^pro^ recognition sequence for autoproteolysis and a C-terminal 6 × His-tag preceded by a HRV M^pro^ recognition sequence. Except for some minor adjustments, the expression and purification of SARS-CoV-2 M^pro^ was performed according to a previously described procedure.^68^ The vector (pGEX-6P-1) containing the coding sequence of the SARS-CoV-2 M^pro^ (obtained from Diamond Light Source, Oxford, UK) was transformed into *E. coli* BL21 (DE3)-T1R competent cells. L-Broth media (Formedium, Norfolk, UK) supplemented with carbenicillin (100 μg/mL) was inoculated with fresh transformants and grown at 37 °C until an OD600 of 1.5 was reached. The starter culture was then used to inoculate the main culture in Auto Induction Media (AIM) Terrific Broth base with trace elements (Formedium, Norfolk, UK) supplemented with 1% glycerol and carbenicillin (100 μg/mL). The cultures were grown at 37 °C until an OD600 of 2 was reached, and the protein expression was continued overnight at 18 °C for 13.5 h. Cells were thereafter harvested by centrifugation (10 min at 4500 gav, 4 °C), resuspended in IMAC lysis buffer (50 mM Tris, 300 mM NaCl, pH 8.0) supplemented with benzonase nuclease (10 μL/1.5 L culture, 250 U/μL, E1014, Merck, Darmstadt, Germany), and disrupted by sonication (4*s*/4s 3 min, 80% amplitude, Sonics Vibracell-VCX750, Sonics & Materials Inc., Newtown, CT, USA). Lysates were centrifuged at 49000 gav for 20 min at 4 °C. The supernatants were filtered (Corning bottle-top vacuum filter, 0.45 μm, Corning, NY), and imidazole was added to a final concentration of 10 mM before loading onto an HisTrap HP 5 mL (Cytiva, Uppsala, Sweden), mounted on an ÄKTAxpress system (Cytiva, Uppsala, Sweden). The column was washed with wash buffer (50 mM Tris, 300 mM NaCl, 25 mM imidazole, pH 8.0), and the bound protein was eluted with elution buffer (50 mM Tris, 300 mM NaCl, 500 mM imidazole, pH 8.0). For crystallization experiments, the protein was further purified by size-exclusion chromatography (SEC) using a HiLoad 16/60 Superdex 200 pg (Cytiva, Uppsala, Sweden) pre-equilibrated with gel filtration buffer (50 mM Tris, 300 mM NaCl, pH 8.0). To remove the His-tag, the protein containing fractions were pooled and treated with HRV 3C protease (1 μg/500 μg target protein, SAE0045, Merck, Darmstadt, Germany) overnight at 4 °C in gel filtration buffer supplemented with 0.5 mM TCEP and 0.5 mM DTT. For the M^pro^ protease activity assay the protein was treated with HRV 3C protease directly after the IMAC purification step, and the buffer was at the same time exchanged by dialysis (dialysis buffer 50 mM Tris, 300 mM NaCl, 0.5 mM TCEP and 0.5 mM DTT, pH 8.0) with a dialysis cassette (Slide-A-Lyzer Dialysis Cassette, 10K MWCO, 3 mL, Thermo Fisher Scientific, Waltham, MA) overnight at 4 °C. The cleaved SARS-CoV-2 M^pro^ samples were subsequently purified by reverse IMAC purification using a HisTrap 1 mL (Cytiva, Uppsala, Sweden). The same wash buffer described above was used, and the flow through was collected. The reverse IMAC purification was followed by a second SEC step using the same column and buffer as described earlier. Fractions containing the target protein were examined by SDS–PAGE, pooled together, and concentrated with Vivaspin 20 mL centrifugal concentrators (10 kDa MWCO, Sartorius, Göttingen, Germany) at 4000 gav, 4 °C. The protein was finally flash frozen in liquid nitrogen and stored at −80 °C.

### M^pro^ Protease Activity Assay

An internally quenched fluorogenic substrate for SARS-CoV-2 M^pro^ (DABCYL-Lys-HCoV-SARS Replicase Polyprotein 1ab (3235–3246)-Glu-EDANS trifluoroacetate salt, > 95% pure) was custom synthesized and obtained from Bachem AG, Switzerland.

The M^pro^ protein used for catalytic activity assays was obtained from the Protein Science Facility (PSF, Karolinska Institutet, Stockholm, Sweden) and is described in a prior section. All test compounds were dissolved to 10 mM stocks in 100% DMSO (Merck KGaA, Darmstadt, Germany) and transferred to Echo LDV source plates (Labcyte, Inc., CA). M^pro^ activity was analyzed by detection of hydrolysis of an internally quenched SARS-CoV-2 M^pro^ substrate. The assay was performed in 20 mM Tris, 50 mM NaCl, and 0.1 mM EDTA (Merck KGaA, Darmstadt, Germany) at room temperature (pH 7.5). Compounds were transferred with an Echo 550 noncontact dispenser (Labcyte, Inc., USA) to Corning 3575 nonbinding 384 well assay plates. M^pro^ (75 nM final concentration) was added to the assay plate using a 16-channel pipet (Integra ViaFlo, BergmanLabora AB, Sweden) and shaken for 15 min at 1000 rpm in an Eppendorf Mixmate. After a pulse centrifugation, the M^pro^ fluorogenic substrate (stock solution at 5 mM in 100% DMSO) was added to the assay plate to a final concentration of 10 μM, thus contributing with 0.2% DMSO in final assay, with a Labcyte Echo 550 noncontact dispenser. After 10 min incubation with shaking at 1000 rpm in an Eppendorf Mixmate and a pulse centrifugation, fluorescence was measured in a PerkinElmer Envision plate reader at ambient temperature with excitation at 340 nm and emission at 490 nm.

### Compound Screening

Screening compounds (Supplementary Data file 1) were purchased from Enamine Ltd. (compound purity >90%, which was confirmed in-house by LC–MS). **GC376** was obtained from Carbosynth, and a freshly synthesized reference sample of **PF-07321332** was kindly provided by the Drugs for Neglected Diseases *initiative* (DND*i*, Switzerland). Using the activity assay described above, compounds were screened against M^pro^ at three concentrations, 5, 15, and 50 μM, respectively, and hits were retested in an 11-point concentration series (1:3 dilutions, starting concentration 50 μM). The dose–response curve was generated using Echo 550 noncontact dispensing from 10 mM compound stocks. Screening results were calculated as percent of M^pro^ activity in each data point (100 × (RFU sample − RFU Blank control)/(RFU DMSO control − RFU Blank control)) using Microsoft Excel with XLfit 5.5, IDBS, Guildford, U.K. A nonlinear fit of 11-point dose−response curves (log(inhibitor) vs response − variable slope (four parameters) and IC_50_ calculations were performed using GraphPad Prism version 9.1.0 for Windows (GraphPad Software, San Diego, CA). Outliers identified by GraphPad Prism were excluded from graphs and analysis in 11-point dose response data.

### Counter Assay Screening

Compounds were evaluated in an 11-point concentration series in the standard M^pro^ activity assay with the addition of 0.01% Triton X-100 to identify aggregating compounds. The compounds were also run with the addition of reducing agent (1 mM DTT). The effect of selected compounds on the activity on human cathepsin S was determined with the SensoLyte 440 cathepsin S Assay Kit (Anaspec, Inc.) used according to the manufacturer’s recommendations. The screen against a panel of nine human proteases (cathepsin K, cathepsin D, cathepsin B, cathepsin L, thrombin, caspase-2, elastase, calpain 1, and trypsin) was performed by Cerep/Panlabs/Eurofins. Compound **19** was tested in duplicate at 10 μM against each enzyme. Reference control compounds were included in each assay.

### Surface Plasmon Resonance (SPR) Biosensor Assays

The SPR experiments were performed using a Biacore S200 instrument and Sensor Chip CM5 (Cytiva, Uppsala, Sweden) at 25 °C. Streptavidin (Sigma) was immobilized by a standard amine coupling procedure. The Sensor Chip CM5 surface was activated by an injection of a 1:1 mixture of EDC and NHS for 7 min at a flow rate 10 μL/min. Streptavidin was diluted to 250 μg/mL in sodium acetate buffer (pH 5.0) and injected over the activated surface at a flow rate 2 μL/min for 10 min. The surface was then deactivated by the injection of 1 M ethanolamine for 7 min. Subsequently, the biosensor chip was conditioned with four pulse injections of 1 M NaCl/50 mM NaOH solution. M^pro^ with a C-terminal Avi-tag (obtained from Diamond Light Source, Oxford, UK) was diluted to 100 μg/mL in 1.02 × running buffer (50 mM TrisHCl, pH 7.5, 0.05% Tween-20) and injected at the flow rate of 2 μL/min, reaching a typical immobilization level of 8000–9000 RU.

After immobilization, compounds were injected over the surface at 5, 20, and 50 μM, at a flow rate 30 μL/min in 50 mM TrisHCl, pH 7.5, 0.05% Tween-20. An association phase was monitored for 60 s and a dissociation phase for 120 s. A solvent correction accounting for 2% DMSO was performed. The data was analyzed using Biacore S200 Evaluation Software, v. 1.1 (Cytiva, Uppsala, Sweden). Selected compounds were further analyzed using a 10-point concentration series. Sensorgrams were double-referenced by subtracting the signals from a reference surface and the signal from one blank injection. For determination of *K*_D_ values, an equation corresponding to a reversible, one-step, 1:1 interaction model was fitted by nonlinear regression analysis to report points taken at the end of the injection, representing steady-state signals. **GC376** and **PF-07321332** were analyzed using a single-cycle kinetics experiment due to no/slow dissociations.

### Protein Crystallization

M^pro^ was crystallized at 20 °C in 96-Well-3-Drop MRC plates (SWISSCI AG, Switzerland) using sitting drop vapor diffusion method and Mosquito pipetting robot (TTP Labtech, UK). Initial crystallization hits (clusters of plate-like crystals) were obtained from 300 nl 1:1 protein to reservoir (100 mM MES pH 6–6.5, 9–11% (m/v) PEG8K) drops and were used to prepare crystallization seeds. After optimization, the final crystallization conditions were as follows: 100 nL of M^pro^ (8.3 mg mL^–1^ in 50 mM Tris pH 8.0, 300 mM NaCl), 50 nL seeds, and 450 nL reservoir solution (100 mM Tris pH 8.25 or 200 mM HEPES pH 7.75, 5% (v/v) DMSO, 12.5% PEG4K). Individual crystals nucleated within 24 h and grew to maximal dimensions within 2 days. Soaking was performed in 100 mM Tris pH 8.25 or 200 mM HEPES pH 7.75, 6.25–15 mM ligand, 5% DMSO, and 20% PEG4K soaking buffers, supplemented with PEG300 to 10% (v/v) for in situ cryo-protection. After 2 h of soaking at 20 °C, crystals were harvested and cryo-cooled in liquid nitrogen.

All diffraction data was collected at cryogenic temperature at the BioMAX beamline (MAX IV Laboratory, Sweden).^56^ Data collection parameters are given in Supplementary Table 1. Data sets were indexed, merged, and scaled on-site and analyzed with the DIMPLE pipeline, implemented within the CCP4 software suite,^57^ using 7K3T as a search model and an optional molecular replacement with PHASER MR.^58^ The data sets of interest were reprocessed with XDS^59^ and AIMLESS, ligand dictionaries were created using AceDRG,^60^ model building and ligand fitting were carried out using *Coot*,^61^ and structure refinement was performed using either Refmac5 or phenix.refine.^62^ Validation of the structures was performed using MolProbity.^63^ Data collection and refinement statistics are shown in Supplementary Table 1. Crystallographic structures and the corresponding structural factors were deposited in the Protein Data Bank (PDB IDs: 7B2U, 7AU4, 7B2J, 7B5Z, 7B77, 7BIJ, 7NEO, 7O46, 7QBB, and 7NBT). Images were generated using PyMOL (v.2.0.6). Omit difference maps were calculated by removing ligand atoms from the structural models, followed by 10 cycles of refinement in Refmac5.

### SARS-CoV-2 Antiviral Activity in Huh7 Cell Culture

The human hepatoma cell line Huh7 was maintained in DMEM (Gibco catalog no. 41965-039) supplemented with 10% fetal bovine serum (FBS), 2% HEPES 1 M (Gibco catalog no. 15630106), 5 mL of sodium bicarbonate 7.5% (Gibco catalog no. 25080-060) 1% nonessential amino acids (NEAA Gibco catalog no. 11140050), and 1% penicillin-streptomycin 10000 U mL^–1^ (Gibco catalog no. 15140148) in a humidified 5% CO_2_ incubator at 37 °C. Assay medium, used for producing virus stocks and antiviral testing, was prepared by supplementing DMEM with 4% FBS, 2% HEPES 1 M, 5 mL of sodium bicarbonate 7.5, and 1% NEAA. To quantify antiviral activity on Huh7 cells, a SARS-CoV-2 virus strain that produces a sufficient cytopathogenic effect (CPE) on this cell line was selected. Passage 6 of the SARS-CoV-2 strain BetaCov/Belgium/GHB-03021/2020 (EPI ISL 407976, 3 February 2020)^64^ was passaged three times on Huh7 cells while selecting those cultures that showed the most CPE. This resulted in a virus stock (passage 9) that confers full CPE on Huh7 (5.6 × 10^4^ median tissue culture infectious dose (TCID_50_) per mL) as well as on Vero E6 cells (1.8 × 10^7^ TCID_50_ per mL). The genotype of this virus stock shows four nucleotide changes as compared with the mother virus stock (P6) and these are currently being analyzed. None of the nucleotide changes occur in the part of the genome that encodes the 3C-like protease, validating this virus stock for testing protease inhibitors. For antiviral testing, Huh7 cells were seeded in 96-well plates (Corning CellBIND 96-well Microplate catalog no. 3300) at a density of 6000 cells per well in assay medium. After overnight growth, cells were treated with the indicated compound concentrations and infected with a multiplicity of infection of 0.005 TCID_50_ per cell of the P9 virus (final volume of 200 μL per well in assay medium). On day 4 post-infection, differences in cell viability caused by virus-induced CPE or by compound-specific side effects were analyzed using MTS (3-(4,5-dimethylthiazol-2-yl)-5-(3-carboxymethoxyphenyl)-2-(4-sulfophenyl)-2*H*-tetrazolium, inner salt). For this, an MTS:phenazine methosulfate (PMS) stock solution (2 mg mL^–1^ MTS (Promega) and 46 μg mL^–1^ PMS (Sigma-Aldrich) in PBS at pH 6–6.5) was diluted 1:20 in MEM without phenol red (Gibco catalog no. 51200038). Medium was aspirated from wells of the test plates, and 70 μL of MTS/PMS solution was added. After 0.5–1 h incubation at 37 °C, absorbance was measured at 498 nm. Cytotoxic effects caused by compound treatment alone were monitored in parallel plates containing mock-infected cells. These experiments were performed in the high-containment BSL3+ facilities of the KU Leuven Rega Institute (3CAPS) under licenses AMV 30112018 SBB 219 2018 0892 and AMV 23102017 SBB 219 2017 0589 according to institutional guidelines.

### SARS-CoV-2 Antiviral Activity in Vero E6 Cell Culture

Vero E6 cells were grown in DMEM (Gibco, 41966029) supplemented with 10% FBS (Gibco, 10500064) and 1× penicillin-streptomycin (Sigma-Aldrich, PA333) and incubated at 37 °C, 5% CO_2_ atmosphere. SARS-CoV-2 was isolated at the Zoonosis Science Center (Uppsala University) from naso-oropharyngeal swab collected from a Swedish patient as described in Nissen et al.^65^ All infection experiments were performed in a biosafety level 3 laboratory (BSL3) at the Zoonosis Science Center (Uppsala University).

### SARS-CoV-2 CPE-Based Antiviral Assay in Vero E6 Cell Culture

Compounds **16**, **17**, and **GC376** were tested at final concentrations ranging from 20 to 0.156 μM with and without a 3 h preincubation step. Compound **ML188** was tested at final concentrations ranging from 100 to 0.78 μM with a 3 h preincubation step. Compound **19** was tested at final concentrations ranging from 20 to 0.019 μM without a preincubation step. Each concentration was tested with triplicates in at least two independent experiments. Vero E6 cells were seeded in 96-well plates at a density of 10^4^ cells/well in a final volume of 100 μL of DMEM (2% FBS, 1× penicillin–streptomycin) and incubated overnight at 37 °C, 5% CO_2_ atmosphere. On the day of the assay, aliquots of the compounds in DMSO (stored at −20 °C) were thawed, serially diluted (1:2) in DMSO, and then further diluted in DMEM (2% FBS, 1× penicillin–streptomycin) to 4× working solutions of the desired final concentrations. Cell media was removed and substituted with 50 μL of fresh cell media (2% FBS, 1× penicillin–streptomycin). Cells were infected at a MOI of 0.01 by adding 25 μL of SARS-CoV-2 solution and treated by adding 25 μL of the compounds’ 4× working solutions giving a total volume of 100 μL (1:4 dilution). Compounds **16**, **17**, **GC376**, and **ML188** were also tested with a 3 h pretreatment step. Vero E6 cells were pretreated with the compounds by removing 25 μL of cell media and adding 25 μL of 4x working solutions of the compounds’ desired final concentrations to the remaining 75 μL of cell media (1:4 dilution). After 3 h of pretreatments, the cell media was removed, cells were washed with 100 μL of PBS, 50 μL of fresh DMEM (2% FBS, 1× penicillin–streptomycin) was added to each well, and cells were retreated and infected as previously explained. Treated–uninfected, infected–untreated, and untreated and infected control wells were also included in triplicates. DMSO concentration in each well was kept to 0.2% (v/v).

After 72 h, the cell media in each well was replaced with 100 μL of fresh DMEM (2% FBS, 1x Penicillin-Streptomycin) to which 10 μL of a 5 mg/mL MTT (Sigma-Aldrich, M2128) solution in PBS was added. Following 4 h incubation, 100 μL of a 10% SDS, 0.01 M HCl solution was added to solubilize the formed formazan crystal. After overnight incubation optical density (OD) at 570 and 690 nm was read using a Tecan infinite M200 PRO plate reader (Tecan Trading AG, Switzerland). OD readings at different wavelengths were subtracted, the resulting values were normalized to the controls, and EC_50_ were determined by nonlinear regression analysis using GraphPad Prism (v.6.0) (GraphPad Software, La Jolla California, USA).

### SARS-CoV-2 Virus Yield Reduction Assay with RT-qPCR

Vero E6 cells were seeded in 96-well plates at a density of 10^4^ cells/well in a final volume of 100 μL of DMEM (2% FBS, 1× penicillin–streptomycin). After overnight incubation at 37 °C, 5% CO_2_, cells were infected with SARS-CoV-2 at a MOI of 0.01 for 1 h, after which the virus inoculum was removed, cells were washed with 100 μL of PBS and 75 μL of fresh DMEM (2% FBS, 1× penicillin–streptomycin) was added to each well. Cells were then treated as explained in the CPE based assay paragraph by adding 25 μL of 4× working solutions of the compounds’ desired final concentrations. Treated–uninfected, infected–untreated, and untreated and infected control wells were also included in triplicates. DMSO concentration in each well was kept to 0.2% (v/v). After 72 h, 50 μL of supernatant from each well was collected and mixed with 150 μL of TRIzol LS (Invitrogen, ThermoFisher Scientific, Waltham, MA) for viral RNA extraction and quantification by RT-qPCR. The remaining supernatant was removed and substituted with 100 μL of fresh DMEM (2% FBS, 1× penicillin–streptomycin). Quantified viral RNA from infected wells treated with different concentrations of the compounds were normalized to the controls and EC_50_ values were determined by nonlinear regression analysis using GraphPad Prism v.6.0 (GraphPad Software, La Jolla CA).

Viral RNA was extracted using the Direct-zol-96 RNA Kit (Zymo Research, Irvine, CA) from the collected supernatant with a sample volume of 200 μL (50 μL of supernatant + 150 μL of TRIzo LS) according to the manufacturer’s protocol. Portions of the SARS-CoV-2 envelope small membrane protein (E) gene was amplified by RT-qPCR, using primers (Thermo Fisher Scientific, Waltham, MA) previously described and the SuperScript III OneStep RT-PCR System with Platinum Taq DNA Polymerase kit (Invitrogen, Thermo Fisher Scientific, Waltham, MA). Target E:^66^ forward primer 5′-ACAGGTACGTTAATAGTTAATAGCGT-3′; reverse primer 5′-TGTGTGCGTACTGCTGCAATAT-3′; and the probe 5′-FAM-ACACTAGCCATCCTTACTGCGCTTCG-TAMRA-3′. The reaction mixture contained 12.5 μL of reaction buffer (a buffer containing 0.4 mM of each dNTP, 3.2 mM MgSO_4_), 0.5 μL of SuperScript III RT/Platinum Taq Mix, 0.5 μL of each primer (10 μM (μM) stock concentrations), 0.25 μL probe (10 μM stock concentration), 2.4 μL of 25 mM magnesium sulfate, 3.35 μL of nuclease-free water, and 5 μL of RNA template. The RT-qPCR assay was performed on a CFX96 Touch Real-Time PCR Detection System (Bio-Rad Laboratories, Hercules CA) under the following conditions: reverse transcription at 55 °C for 30 min and 95 °C for 3 min, followed by 45 cycles of denaturation at 95 °C for 15 s, extension at 57 °C for 30 s, and collecting the fluorescence signal at 68 °C for 30 s.

All samples were run in triplicate. The corresponding number of copies for each Ct was calculated from a standard curve prepared with synthetic DNA gene fragments (gBLOCKs; IDT, San Jose, CA) with a five-base-pair deletion in the amplified regions of the viral genome diluted in deionized, nuclease-free water to concentrations of 10^3^–10^5^ copies per μL. The five-base-pairs were deleted to be able to distinguish between viral RNA and gBLOCKs during sequencing. The LODs for both genes were 10^1^ copies per μL. The relative fluorescence unit (RFU) data were obtained from the CFX Maestro Software (Bio-Rad CFX Maestro for Mac 1.1 Version 4.1.2434.0214, Bio-Rad Laboratories, Hercules, CA).

### SARS-CoV-1 Antiviral Activity in Vero E6 Cell Culture

The cell line Vero E6 was maintained in DMEM (Gibco catalog no. 41965-039) supplemented with 10% fetal bovine serum (FBS), 2% HEPES 1 M (Gibco catalog no. 15630106), 5 mL of sodium bicarbonate 7.5% (Gibco catalog no. 25080-060), 1% nonessential amino acids (NEAA Gibco catalog no. 11140050), and 1% penicillin–streptomycin 10000 U mL^–1^ (Gibco catalog no. 15140148) in a humidified 5% CO_2_ incubator at 37 °C. Assay medium, used for producing virus stocks and antiviral testing, was prepared by supplementing DMEM with 4% FBS, 2% HEPES 1 M, 5 mL of sodium bicarbonate 7.5, and 1% NEAA. To quantify antiviral activity on Vero E6 cells, a SARS-CoV-1 virus strain that produces sufficient cytopathogenic effect (CPE) on this cell line was selected. SARS-CoV-1 strain 200300592 was obtained from the Centers for Disease Control and Prevention (CDC, Atlanta, GA) and passaged three times on Vero E6 cells while selecting those cultures that showed the most CPE. This resulted in a virus stock (passage 3) that confers full CPE on Vero E6 cells (3.4 × 10^7^ TCID_50_ per mL). For antiviral testing, Vero E6 cells were seeded in 96-well plates (Corning CellBIND 96-well Microplate catalog no. 3300) at a density of 8000 cells per well in assay medium. After overnight growth, cells were treated with the indicated compound concentrations and infected with a multiplicity of infection of 0.02 TCID_50_ per cell of the P3 virus (final volume of 200 μL per well in assay medium). On day 4 post infection, differences in cell viability caused by virus-induced CPE or by compound-specific side effects were analyzed using MTS (3-(4,5-dimethylthiazol-2-yl)-5-(3-carboxymethoxyphenyl)-2-(4-sulfophenyl)-2*H*-tetrazolium, inner salt). For this, an MTS:phenazine methosulfate (PMS) stock solution (2 mg mL^–1^ MTS (Promega) and 46 μg mL^–1^ PMS (Sigma-Aldrich) in PBS at pH 6–6.5) was diluted 1:20 in MEM without phenol red (Gibco catalog no. 51200038). Medium was aspirated from wells of the test plates and 70 μL of MTS/PMS solution was added. After 0.5–1 h incubation at 37 °C, absorbance was measured at 498 nm. Cytotoxic effects caused by compound treatment alone were monitored in parallel plates containing mock-infected cells. These experiments were performed in the high-containment BSL3+ facilities of the KU Leuven Rega Institute (3CAPS) under licenses AMV 30112018 SBB 219 2018 0892 and AMV 23102017 SBB 219 2017 0589 according to institutional guidelines.

### MERS-CoV Antiviral Activity in Huh7 Cell Culture

Huh7 cells were cultured in Dulbecco’s modified Eagle’s medium (DMEM; Lonza) with 8% fetal calf serum (Bodinco), 2 mM l-glutamine (Sigma-Aldrich), 100 IU/mL penicillin, and 100 g/mL streptomycin (Sigma-Aldrich). After infection, cells were kept in Eagle’s minimal essential medium (EMEM; Lonza) with 25 mM HEPES (Lonza), 2% FCS, l-glutamine, and antibiotics. MERS-CoV-Jordan-N3/2012 (Genbank accession no. KC776174) stocks were grown on Vero E6 cells, and titers were determined by plaque assay on the same cells. All experiments with infectious MERS-CoV were performed at the LUMC biosafety level 3 facilities.

### MERS-CoV CPE-Based Antiviral Assay

Huh7 cells (1.5 × 10^4^ cells/well) were seeded in 96-well plates 1 day before infection. Cells were incubated with 100 μL volumes of 2-fold serial dilutions of compound in infection medium, followed by infection with 225 PFU of MERS-CoV in 50 μL, yielding a total assay volume of 150 μL (highest compound concentration 2 μM). In parallel, noninfected cells in the same plate were treated with the same dilution series of compound to assess cytotoxicity. Plates were incubated for 42 h at 37 °C, after which cell viability was quantified with the CellTiter-96 aqueous nonradioactive cell proliferation kit (Promega). After incubation for ∼1.5 h, absorption at 495 nm was measured with an EnVision multilabel plate reader (PerkinElmer). Cell viability was normalized against the readings for noninfected untreated cells (100%). EC_50_ (compound concentration that reduces virus-induced cell death by 50%) values were determined by nonlinear regression using GraphPad Prism v8.0. Experiments, consisting of biological quadruplicates, were repeated twice.

### Metabolic Stability in the Presence of Human Liver Microsomes

Metabolic stability was determined in 0.5 mg/mL human liver microsomes at a compound concentration of 1 μM in 100 mM KPO_4_ buffer pH 7.4 in a total incubation volume of 500 μL. The reaction was initiated by addition of 1 mM NADPH. At various incubation times, i.e., at 0, 5, 10, 20, 40, and 60 min, a sample was withdrawn from the incubation, and the reaction was terminated by addition of cold acetonitrile with warfarin as an internal standard. The amount of parent compound remaining was analyzed by liquid chromatography coupled to triple quadrupole mass spectrometry (LC–MS/MS).

### Plasma Protein Binding and Plasma Stability in Human Plasma

Pooled human plasma was provided by Uppsala Academic Hospital and was collected from two donors (nonsmoking) (citric acid). In brief, 0.2 mL of the plasma (50% plasma, 50% isotonic buffer) test solution (typically 10 μM final compound concentration) was transferred to the membrane tube in the RED insert (ThermoFisher Scientific). Then 0.35 mL of isotonic phosphate buffer pH 7.4 was added to the other side of the membrane. The 96-well base plate was then sealed with an adhesive plastic film (Scotch Pad) to prevent evaporation. The sample was incubated with rapid rotation (≫900 rpm) on a Kisker rotational incubator at 37 °C for 4 h to achieve equilibrium. Prior to LC–MS/MS analysis, the plasma and buffer sample were treated by the addition of methanol (1:3) containing warfarin as the internal standard to precipitate proteins. The standard curve was created using the plasma standard. The plate was then sealed and centrifuged, and the supernatant was analyzed by LC−MS/MS.

### Caco-2 Cell Permeability Assay

Caco-2 cell monolayers (passage 94–105) were grown on permeable filter support and used for transport study on day 21 after seeding. Prior to the experiment a drug solution of 10 μM was prepared and warmed to 37 °C. The Caco-2 filters were washed with prewarmed HBSS prior to the experiment, and thereafter, the experiment was started by applying the donor solution on the apical or basolateral side. The transport experiments were carried out at pH 7.4 in both the apical and basolateral chamber. The experiments were performed at 37 °C and with a stirring rate of 500 rpm. The receiver compartment was sampled at 15, 30, and 60 min and at 60 min also a final sample from the donor chamber was taken in order to calculate the mass balance of the compound. The samples (100 μL) were transferred to a 96-well plate containing 100 μL of methanol and warfarin as IS and was sealed until analysis by LC−MS/MS.

### Liquid Chromatography Coupled to Triple Quadrupole Mass Spectrometry (LC–MS/MS)

The test compounds were optimized on a Waters Acquity UPLC XEVO TQ-S micro system (Waters Corp.) operating in multiple reaction monitoring (MRM) mode with positive or negative electrospray ionization. Compounds were optimized by using the QuanOptimize software (Waters Corp.). The MS conditions listed in Table 2 were used.

**Table 2:** MS Conditions

transition *m*/*z*	dwell time (s)	cone voltage	collision energy
324.4 > 128.04	0.028	10	60
324.4 > 171.03	0.028	10	50

For chromatographic separation, a C_18_ BEH 1.7 μm column was used with a general gradient of 5–1000% of mobile phase B over a total running time of 2 min. Mobile phase A consisted of 0.1% formic acid in purified water and mobile phase B of 0.1% formic acid in 100% acetonitrile. The flow rate was set to 0.5 mL/min, and 5 μL of the sample was injected.

## Supporting Information Available

Crystallographic data collection and refinement statistics, physicochemical properties of compounds, counter screen data, dose–response curves, SPR sensorgrams, biological evaluation of key compounds, results from docking to M^pro^ mutants, selectivity of compound **19**, experimental details and characterization (NMR) of synthesized compounds (PDF)
Chemical structures, SMILES, chemical identifiers, inhibition assay data, binding assay data of compounds (XLSX)

## Notes

Crystal structures have been deposited in the PDB with accession codes: 7B2U, 7AU4, 7B2J, 7B5Z, 7B77, 7BIJ, 7NEO, 7O46, 7QBB, and 7NBT.
