# Supplementary material for: Ultralarge Virtual Screening Identifies SARS-CoV-2 Main Protease Inhibitors with Broad-Spectrum Activity against Coronaviruses
Source: J Am Chem Soc. 2022 Feb 10;144(7):2905–20. doi: 10.1021/jacs.1c08402 (PMC8848513; doi:10.1021/jacs.1c08402)
Supplement: Supplementary file 1 — ja1c08402_si_001.pdf [file ja1c08402_si_001.pdf]

Ultralarge Virtual Screening Identifies SARS-CoV-2 Main Protease Inhibitors with Broad-Spectrum Activity against Coronaviruses

Andreas Luttens[1], Hjalmar Gullberg[2,‡], Eldar Abdurakhmanov[3,‡], Duy Duc Vo[1,‡], Dario Akaberi[4,‡], Vladimir O. Talibov[5], Natalia Nekhotiaeva[2], Laura Vangeel[6,7], Steven De Jonghe[6,7], Dirk Jochmans[6,7], Janina Krambrich[4], Ali Tas[8], Bo Lundgren[2], Ylva Gravenfors[9], Alexander J. Craig[10], Yoseph Atilaw[11], Anja Sandström[10], Lindon W. K. Moodie[10,12], Åke Lundkvist[4], Martijn J. van Hemert[8], Johan Neyts[6,7], Johan Lennerstrand[13], Jan Kihlberg[11], Kristian Sandberg[10,14,15], U. Helena Danielson[3], Jens Carlsson[1,*]

[1]Science for Life Laboratory, Department of Cell and Molecular Biology, Uppsala University, SE-75124 Uppsala, Sweden

[2]Science for Life Laboratory, Biochemical and Cellular Assay Facility, Drug Discovery and Development Platform, Department of Biochemistry and Biophysics, Stockholm University, SE-17121 Solna, Stockholm, Sweden

[3]Science for Life Laboratory, Department of Chemistry-BMC, Uppsala University, SE-75123 Uppsala, Sweden

[4]Department of Medical Biochemistry and Microbiology, Zoonosis Science Center, Uppsala University, SE-75123 Uppsala, Sweden

[5]BioMax beamline, MAX IV Laboratory, Fotongatan 2, SE-22484 Lund, Sweden

[6]KU Leuven Department of Microbiology, Immunology and Transplantation, Rega Institute, Laboratory of Virology and Chemotherapy, 3000 Leuven, Belgium

[7]Global Virus Network, Baltimore, Maryland 21201, United States

[8]Department of Medical Microbiology, Leiden University Medical Center, 2333ZA Leiden, The Netherlands

[9]Science for Life Laboratory, Drug Discovery & Development Platform, Department of Organic Chemistry, Stockholm University, SE-17121 Solna, Stockholm, Sweden

[10]Department of Medicinal Chemistry, Uppsala University, SE-75123 Uppsala, Sweden

[11]Department of Chemistry-BMC, Uppsala University, SE-75123 Uppsala, Sweden

[12]Uppsala Antibiotic Centre, Uppsala University, SE-75123 Uppsala, Sweden

[13]Department of Medical Sciences, Section of Clinical Microbiology, Uppsala University, SE-75185 Uppsala, Sweden

[14]Department of Physiology and Pharmacology, Karolinska Institutet, SE-17177 Stockholm, Sweden

[15]Science for Life Laboratory, Drug Discovery & Development Platform, Department of Medicinal Chemistry, Uppsala Biomedical Center, Uppsala University, SE-75123 Uppsala, Sweden

Table of Contents

Page

Supplementary Tables

Table 1. Crystallographic data collection and refinement statistics. S3
Table 2. Properties of compounds **1**,**16**-**21** and reference inhibitors. S7
Table 3. Summary of counter screens for compounds **16**-**21**, **GC376** and **PF-07321332**. S8
Table 4. Summary of potencies of compounds **16a**, **16b**, **17a** and **17b**. S9
Table 5. Enzyme inhibition induced by compound **19** for a set of human proteases. S10
Table 6. CC_{50} for compounds **16**, **17**, **19** and **PF-07321332**. S11
Table 7. Docking to M^{pro} active site mutants. S12

Supplementary Figures

Fig. 1. Enzyme inhibition assays (M^{pro}) of compounds **16**-**21**, **X77**, and **ML188**. S13
Fig. 2. Analysis of interactions between inhibitors and M^{pro} using an SPR assay. S14
Fig. 3. Crystal structure of compound **13** in complex with M^{pro}. S18
Fig. 4. Optimization of compound **2** and predicted binding mode of compound **20**. S19
Fig. 5. Summary of counter screens for compounds **16**-**19**, **GC376** and **PF-07321332**. S20
Fig. 6. CPE inhibition data for compounds **16** and **17** in Vero E6 and Huh7 cells. S21
Fig. 7. Inhibitory effect of compounds **16**, **17**, **19**, and **GC376** on viral replication. S22
Fig. 8. CPE inhibition data for compounds **16** and **17** without preincubation in Vero E6 cells. S23
Fig. 9. Relative configuration of stereoisomers (**16a**, **16b**, **17a** and **17b**) via NOESY NMR. S30

Methods

Synthesis procedures S24
Stereoisomeric separation S29
Structural elucidation of stereoisomers S30
Chemical shifts of synthesized compounds S31
NMR Spectra S48
References S71

Supplementary Table 1a. Data collection parameters

Beamline	BioMAX MAX IV
Wavelength (Å)	0.976
Beam size (μM)	50
Temperature (K)	100
Resolution (Å)	1.6-1.7
Images	3600
Omega range per image (°)	0.1
Exposure per image (s)	0.011
Transmission (%)	30.00
Flux (photon s^{-1})	$0.9\text{-}1.2\times10^{12}$

Supplementary Table 1b. Data collection and refinement statistics. Values given in parentheses are for the highest resolution shell. RMS deviations for bond geometries were calculated with MolProbity[63]

PDB ID	7B2U	7AU4	7B2J	7B5Z
Ligand Enamine ID	Z3349773787	Z1470552630	Z73239323	Z1127023064
Ligand identifier	Compound **1**	Compound **3**	Compound **5**	Compound **6**
**Data collection**				
Space group	$P\ 1\ 2_1\ 1$	$C\ 1\ 2\ 1$	$C\ 1\ 2\ 1$	$C\ 1\ 2\ 1$
Unit cell, a, b, c (Å)	44.52, 53.68, 114.51	113.83, 53.23, 44.59	114.55, 53.65, 44.57	114.46, 53.66, 44.48
Unit cell, a, b, c (°)	90.00, 100.59, 90.00	90, 102.44, 90	90.00, 100.56, 90.00	90.00, 100.51, 90.00
Resolution (Å)	53.68-1.55	55.58-1.82	48.44-1.55	48.63-1.63
Highest resolution shell (Å)	1.58-1.55	1.86-1.82	1.58-1.55	1.68-1.63
R_{merge}	0.057 (0.964)	0.056 (0.962)	0.063 (0.65)	0.055 (0.312)
R_{pim}	0.036 (0.776)	0.038 (0.461)	0.04 (0.513)	0.035 (0.209)
Reflections	481597 (13748)	119963 (7311)	244033 (6883)	206479 (8580)
Unique reflections	74540 (3130)	23453 (1401)	37807 (1554)	31557 (1437)
I/σ(I)	10.1 (1.3)	8.4 (1.5)	13.2 (2.1)	18.6 (5.2)
$CC_{1/2}$	0.999 (0.708)	0.999 (0.707)	0.999 (0.727)	0.999 (0.948)
Completeness (%)	96.5 (82.6)	99.8 (99.9)	97.8 (81.4)	98.6 (92.9)
Multiplicity	6.5 (4.4)	5.1 (5.2)	6.5 (4.4)	6.5 (6)
**Refinement**				
N. refl. Test set	3649	2222	1914	1504
R/R_{free} (%/%)	0.21/0.25	0.17/0.21	0.16/0.20	0.16/0.20
Ramachandran plot (%)				
Favored	98.18	98.35	98.67	98.67
Outliers	0	0.33	0	0
Rotamer outliers (%)	0.19	0	0.73	0.37
RMS deviations				
Bond (Å)	0.008	0.006	0.008	0.02
Angle (°)	1.14	0.934	1.33	1.59
Clashscore	3.94	4.04	2.65	4.27
Nr. atoms/Av. B-factor				
Protein	4796/27.68	2358/40.43	2434/24.27	2434/21.34
Ligand	74/42.04	30/49.86	44/36.81	43/33.05
Water	525/36.97	135/45.45	357/35.55	266/28.77

(**Supplementary Table 1b** Continued.)

PDB ID	7B77	7BIJ	7NEO	7O46
Ligand Enamine ID	Z1022393778	Z4685004274	Z3798380755	-
Ligand identifier	Compound **8**	Compound **13**	Compound **15**	Compound **17**
**Data collection**				
Space group	*C* 1 2 1	*C* 1 2 1	*P* 1 2_1 1	*C* 1 2 1
Unit cell, a, b, c (Å)	114.54, 53.60, 44.57	114.75, 53.84, 44.59	44.57, 53.82, 114.77	113.67, 54.06, 44.93
Unit cell, a, b, c (°)	90.00, 100.50, 90.00	90.00, 100.58, 90.00	90.00, 100.49, 90.00	90.00, 101.47, 90.00
Resolution (Å)	48.4-1.6	31.91-1.47	48.58-1.64	48.64-2.23
Highest resolution shell (Å)	1.63-1.6	1.50-1.47	1.67-1.64	2.31-2.23
R_{merge}	0.055 (0.42)	0.058 (0.459)	0.065 (0.431)	0.052 (1.336)
R_{pim}	0.035 (0.324)	0.037 (0.372)	0.045 (0.302)	0.023 (0.587)
Reflections	230000 (7728)	292309 (8348)	366074 (18018)	89181 (8644)
Unique reflections	34900 (1587)	44811 (1891)	65712 (3232)	13122 (1242)
I/σ(I)	15 (3.2)	13.7 (2.4)	11.3 (3.2)	14.6 (1.4)
$CC_{1/2}$	0.999 (0.931)	0.998 (0.839)	0.998 (0.94)	0.998 (0.537)
Completeness (%)	99.3 (92.5)	98.5 (85.4)	99.9 (100)	99.7 (97.7)
Multiplicity	6.6 (4.9)	6.5 (4.4)	5.6 (5.6)	6.8 (7.0)
**Refinement**				
N. refl. Test set	1726	2183	3193	638
R/R_{free} (%/%)	0.17/0.21	0.18/0.21	0.20/0.24	0.20/0.25
Ramachandran plot (%)				
Favored	99	99.01	98.5	94.59
Outliers	0	0	0	0.34
Rotamer outliers (%)	0	0	0.19	1.54
RMS deviations				
Bond (Å)	0.01	0.01	0.01	0.014
Angle (°)	1.13	1.82	1.88	1.85
Clashscore	2.91	3.1	2.61	2.37
Nr. atoms/Av. B-factor				
Protein	2393/26.7	2414/23.09	4781/19.25	2329/84.78
Ligand	45/43.32	38/39.93	70/31.66	24/116.54
Water	228/34.14	322/34.3	492/27.84	2/66.68

(**Supplementary Table 1b** Continued.)

PDB ID	7QBB	7NBT
Ligand Enamine ID	-	Z226482770
Ligand identifier	Compound **18**	Compound **21**
**Data collection**		
Space group	*C* 1 2 1	*C* 1 2 1
Unit cell, a, b, c (Å)	113.77, 53.82, 44.99	114.58, 53.77, 44.52
Unit cell, a, b, c (°)	90.00, 101.46, 90.00	90.00, 100.53, 90.00
Resolution (Å)	48.47-2.00	56.33-1.63
Highest resolution shell (Å)	2.07-2.00	1.66-1.63
R_{merge}	0.066 (1.654)	0.06 (0.747)
R_{pim}	0.041 (1.025)	0.043 (0.55)
Reflections	122406 (11897)	166095 (8406)
Unique reflections	18097 (1754)	32705 (1649)
I/σ(I)	11.9 (1.1)	11.1 (2)
$CC_{1/2}$	0.997 (0.534)	0.998 (0.774)
Completeness (%)	99.7 (98.3)	98.1 (100)
Multiplicity	6.8 (6.8)	5.1 (5.1)
**Refinement**		
N. refl. Test set	891	1621
R/R_{free} (%/%)	0.21/0.26	0.19/0.23
Ramachandran plot (%)		
Favored	96.99	98.33
Outliers	0.33	0
Rotamer outliers (%)	0.77	0.75
RMS deviations		
Bond (Å)	0.014	0.01
Angle (°)	1.78	1.65
Clashscore	3.2	2.72
Nr. atoms/Av. B-factor		
Protein	2347/69.76	2389/29.08
Ligand	34/85.02	34/37.96
Water	18/68.32	149/34.37

Supplementary Table 2. Properties of compounds **1**, **16-21**, and reference inhibitors.

Compound	MW (g mol^{-1})	cLogP[a]	HAC[b]	LE (kcal mol^{-1} HAC^{-1})[c]	LLE (kcal mol^{-1})[d]
**1**	291.3	2.1	21	0.30	8.6
**16**	323.4	2.7	24	0.39	13.1
**17**	321.3	2.3	24	0.39	13.4
**18**	343.4	2.8	26	0.36	12.8
**19**	377.8	3.4	27	0.37	13.7
**20**	356.8	2.6	25	0.29	9.6
**21**	312.4	2.6	22	0.35	10.3
**X77** (rac)	459.6	4.0	34	0.23	9.2
**ML188** (rac)	433.6	4.2	32	0.22	7.9

[a] Calculated log of partition coefficient (n-octanol / water). [b] Heavy atom count. [c] Ligand efficiency: negative change in free energy divided by number of ligand heavy atoms. Change in free energy was derived from the K_D value obtained by steady state SPR interaction analysis (Supplementary Figure 2). [d] Ligand-lipophilic efficiency: negative log of the ratio between the K_D value obtained by steady state SPR interaction analysis and the calculated partition coefficient (n-octanol / water).

Supplementary Table 3. Summary of counter screens for compounds **16-21**, **GC376** and **PF-07321332**.

Cmpd	M^{pro} (IC_{50}, μM)[a]			Cathepsin S (IC_{50}, μM)[a]
	Enzyme inhibition assay	+ DTT[b]	+ Triton X-100[c]	
**16**	0.46 ± 0.06 (3)	0.34 ± 0.05 (3)	0.53 ± 0.04 (2)	> 50 (1)
**17**	0.33 ± 0.04 (3)	0.29 ± 0.04 (3)	0.31 ± 0.08 (2)	> 50 (1)
**18**	0.39 ± 0.05 (3)	0.46 ± 0.08 (2)	0.42 ± 0.06 (2)	> 50 (1)
**19**	0.077 ± 0.006 (3)	0.080 ± 0.004 (1)	0.10 ± 0.01 (1)	> 50 (1)
**20**	7.2 ± 3.1 (3)	10 ± 1 (2)	9.5 ± 8.2 (2)	> 50 (1)
**21**	2.1 ± 0.7 (3)	2.2 ± 1.2 (2)	2.3 ± 0.7 (2)	> 50 (1)
**GC376**	0.073 ± 0.004 (6)	0.067 ± 0.005 (4)	0.10 ± 0.01 (3)	0.0022 ± 0.0002 (2)
**PF-07321332**	0.033 ± 0.002 (2)	-	-	5.7 ± 2.1 (1)

[a] IC_{50} values are expressed as mean ± SEM from (n>1) independent experiments or mean ± standard error of the fit SE (n=1). [b] Addition of dithiothreitol (DTT), a reducing agent, to identify compounds with covalent mechanism of action. [c] Addition of Triton X-100 (0.01%) as a control for promiscuous inhibition by colloidal aggregation.

Supplementary Table 4. Summary of potencies of compounds **16a**, **16b**, **17a** and **17b**.

Cmpd	M^{pro} (IC_{50}, μM)[a]	SARS-CoV-2 (EC_{50}, μM)[b]
**16a**	0.24 ± 0.02	0.95 ± 0.10
**16b**	1.4 ± 0.3	16 ± 1
**17a**	0.37 ± 0.05	1.5 ± 0.1
**17b**	0.35 ± 0.03	1.4 ± 0.1

[a] IC_{50} values are expressed as mean ± SEM from three independent experiments. [b] Inhibitory effect of compounds on CPE induced by SARS-CoV-2 infection in Huh7 cells. EC_{50} values are expressed as mean ± SEM from two independent experiments.

Supplementary Table 5. Enzyme inhibition induced by compound **19** for a set of human proteases.

**Human Protease**	**IC_{50} (μM)[a]**
Cathepsin K	>10
Cathepsin D	>10
Cathepsin B	>10
Cathepsin L	>10
Thrombin	>10
Caspase-2	>10
Elastase	>10
Calpain 1	>10
Trypsin	>10

[a] Compound was tested in duplicate at 10 μM in a Cerep/Panlabs/Eurofins protease panel screen.

Supplementary Table 6. CC_{50} (compound concentration that reduces viability of uninfected cells to 50%) for compounds **16**, **17**, **19** and **PF-07321332**.

Cmpd	Vero E6 (μM)[a]	Huh7 (μM)[b]
**16**	>20	>100
**17**	>20	>100
**19**	>20	>5
**PF-07321332**	-	>100

[a] Highest tested concentration was 20 μM. [b] Highest tested concentration was 100 μM.

Supplementary Table 7. Docking of compound **19** to homology models of M^{pro} active site mutants.

**Mutant**[a]	**Ligand RMSD (Å)**[b]	**Docking score (kcal mol^{-1})**[c]
H41Y	0.5	−53.8
H41L	1.1	−45.0
H41Q	1.2	−38.6
C44S	1.2	−54.9
M49L	1.0	−50.2
M49V	1.3	−35.9
M49T	1.2	−42.3
M49I	1.1	−48.5
P52S	1.2	−41.3
P52L	0.9	−42.8
L141I	1.1	−51.4
L141F	0.8	−55.5
N142H	1.3	−48.6
N142D	1.2	−44.1
N142S	0.6	−53.4
N142I	0.7	−55.5
S144A	0.8	−54.6
C145S	0.9	−51.2
C145Y	0.7	−35.9
C145F	0.6	−52.6
M165L	1.2	−51.7
M165V	1.1	−50.0
M165K	1.2	−48.7
M165I	1.4	−34.8
E166G	1.0	−45.7
H172Y	0.9	−53.4
Q189K	1.2	−50.6
Q189L	0.9	−54.7
Q189H	0.9	−54.8

[a] Active site residues were identified as residues within 7 Å of the co-crystalized inhibitor **X77** in M^{pro} crystal structure (PDB accession code 6W63). [b] The RMSD value of the docking pose of compound **19** was calculated to the corresponding atoms of compound **18** bound to M^{pro} in crystal structure (PDB accession code: 7QBB) after alignment to the structure used in the virtual screen (PDB accession code: 6W63). [c] DOCK3.7 docking energy score.

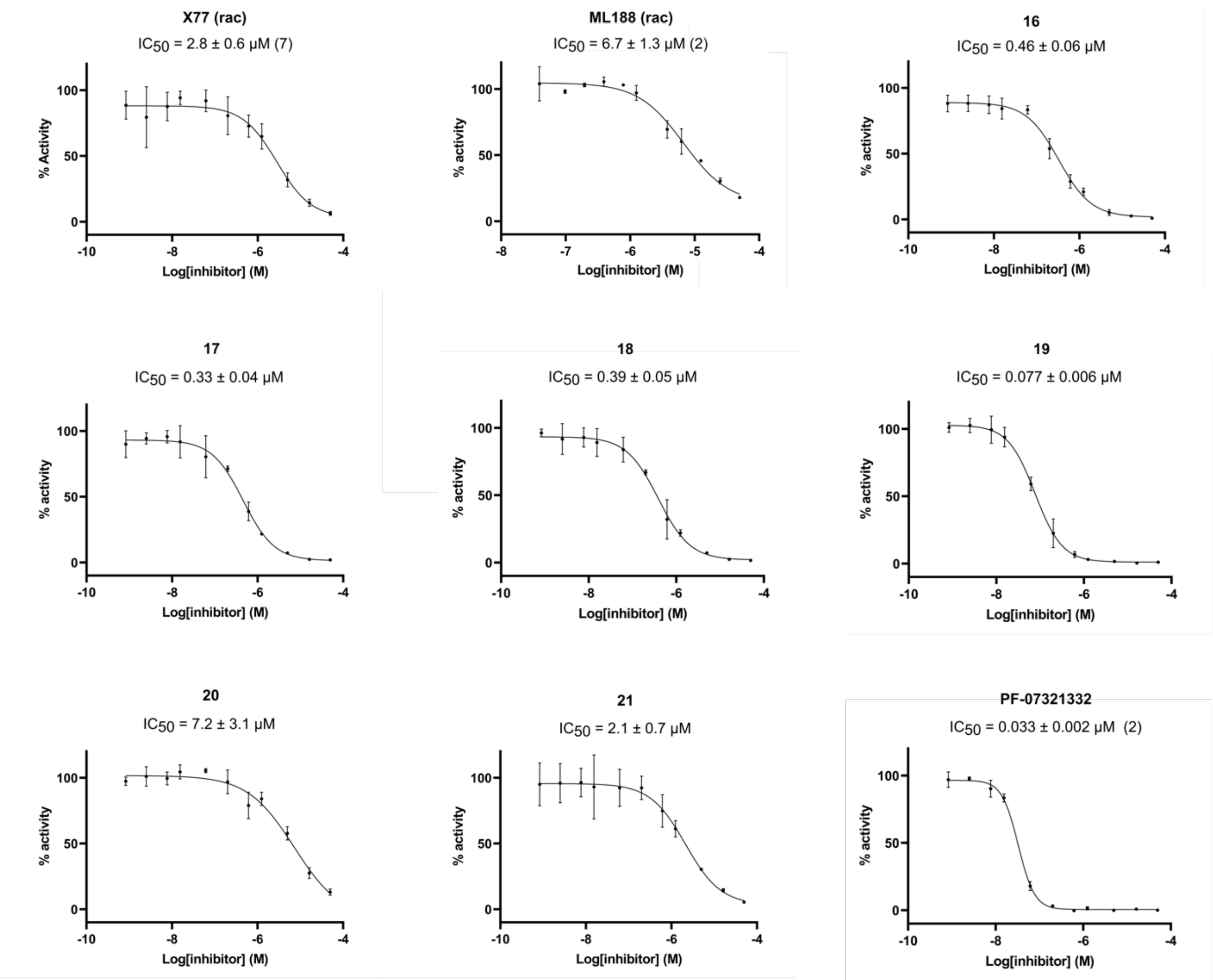

Supplementary Fig. 1. Inhibition of M^{pro} activity by compounds **16**-**21**, **ML188** (rac), **X77** (rac) and **PF-07321332**. Data points represent mean ± SD from 2-7 independent experiments. IC_{50} values are expressed as mean ± SEM from three independent experiments, unless stated otherwise. Note that the data for **PF-07321332** in this steady-state assay is not directly comparable to those for the other compounds due to its covalent mechanism.

X77 (rac)

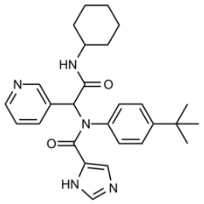

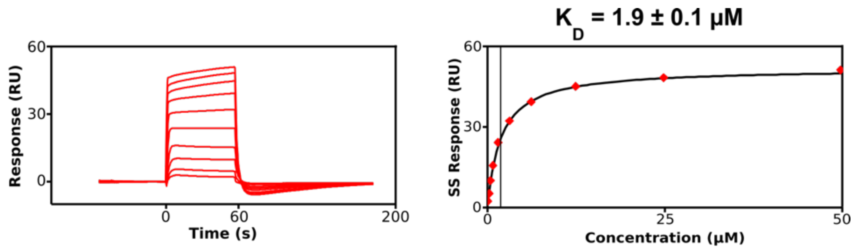

1

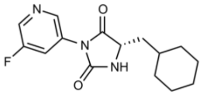

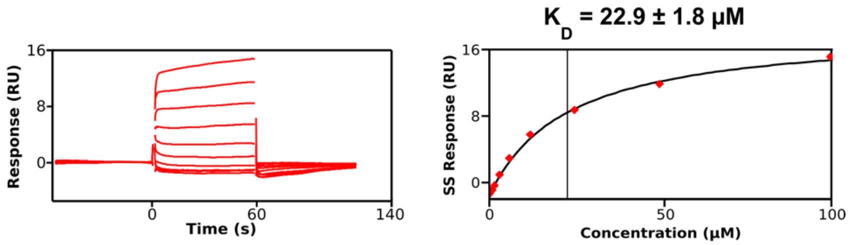

2

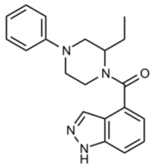

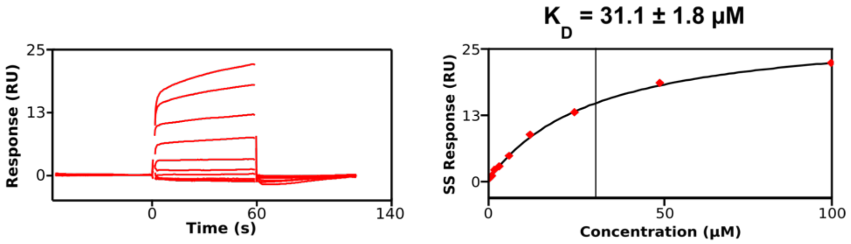

3

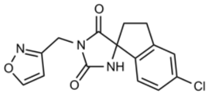

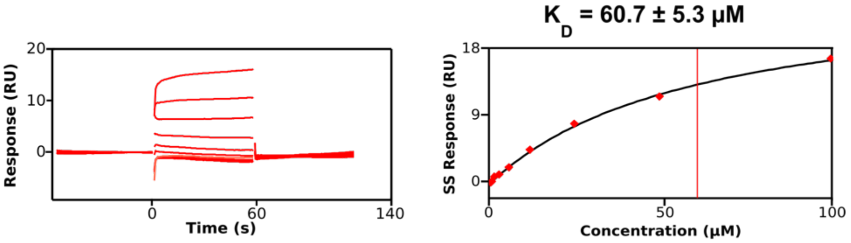

4

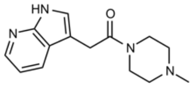

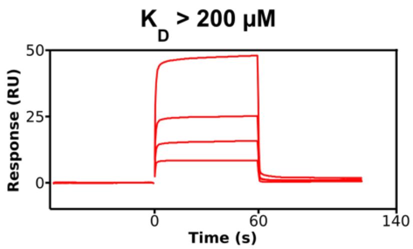

5

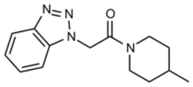

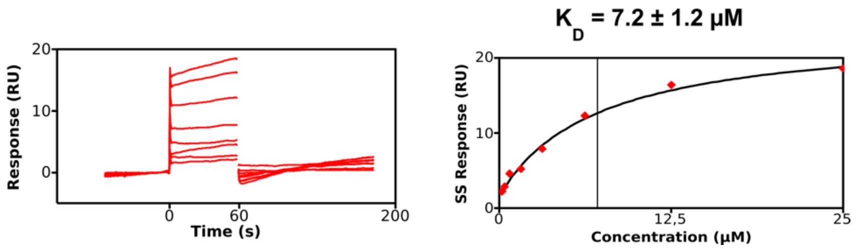

6 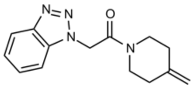

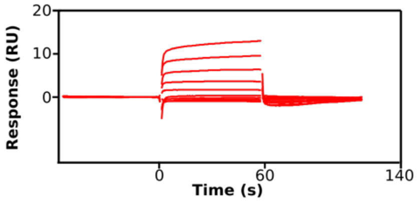

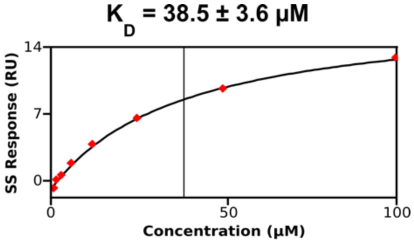

7 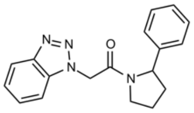

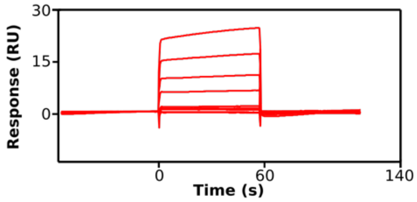

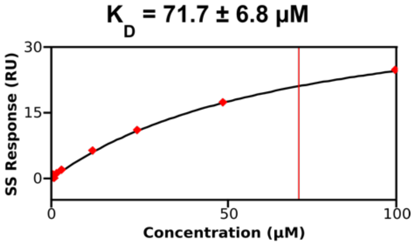

8 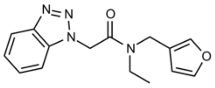

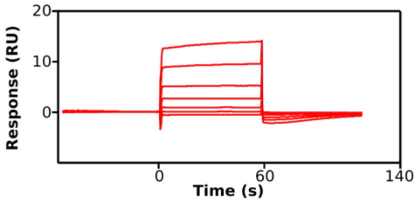

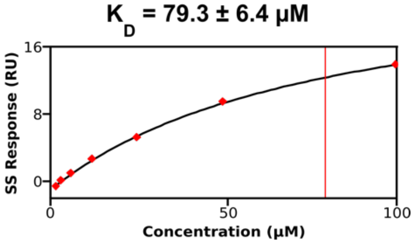

9 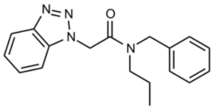

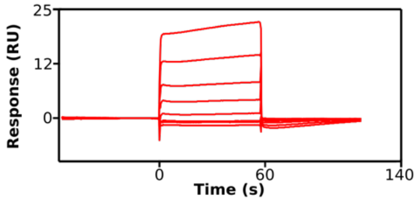

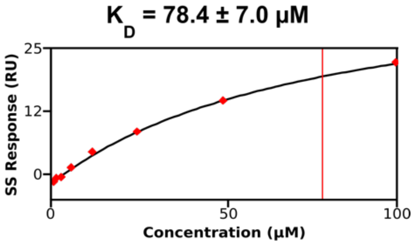

11 (rac) 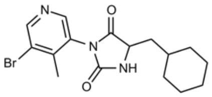

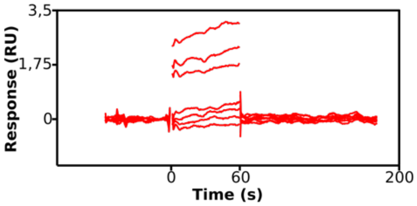

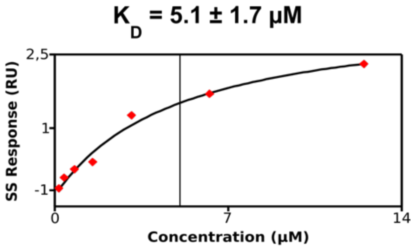

15 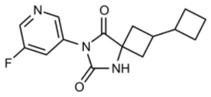

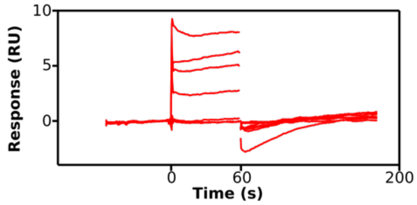

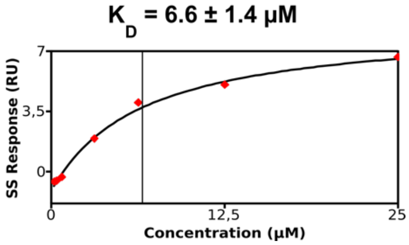

16

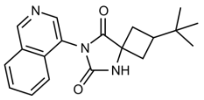

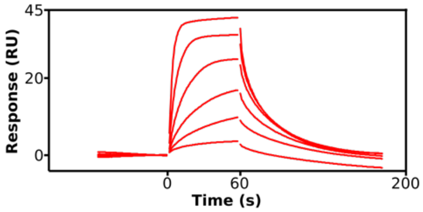

K_D = 141 ± 5 nM

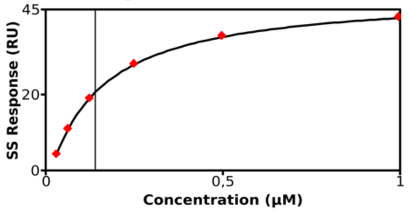

17

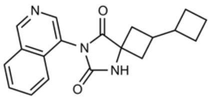

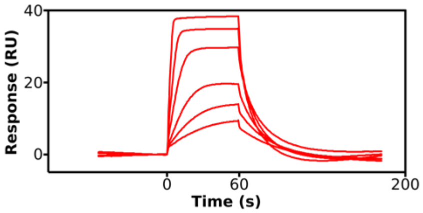

K_D = 152 ± 37 nM

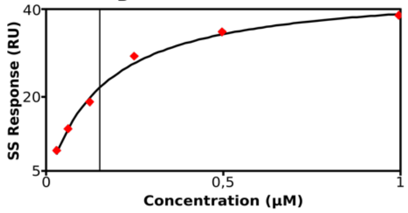

18

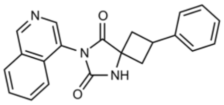

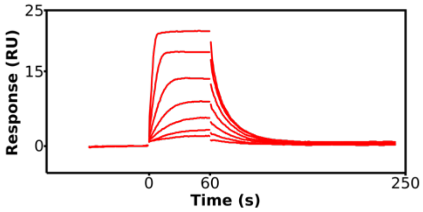

K_D = 168 ± 7 nM

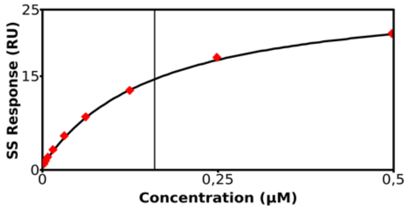

19

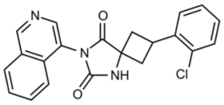

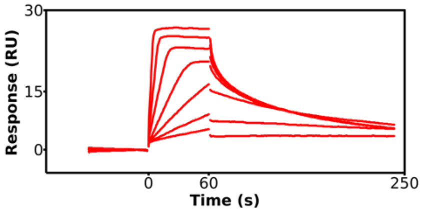

K_D = 38 ± 17 nM

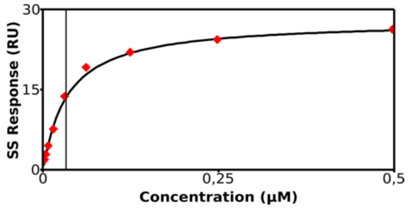

20

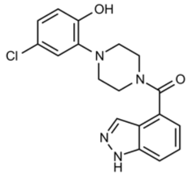

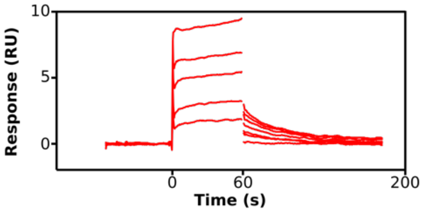

K_D = 4.7 ± 1.7 µM

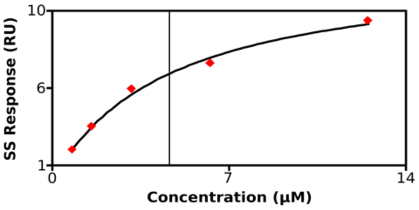

21

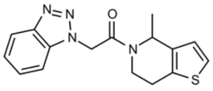

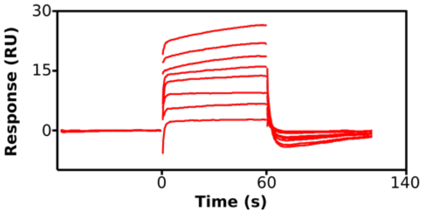

K_D = 2.3 ± 1.3 µM

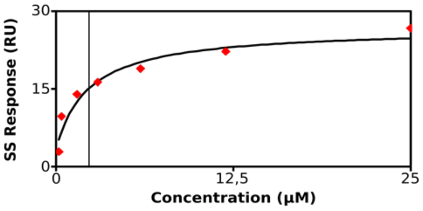

22

GC376

PF-07321332

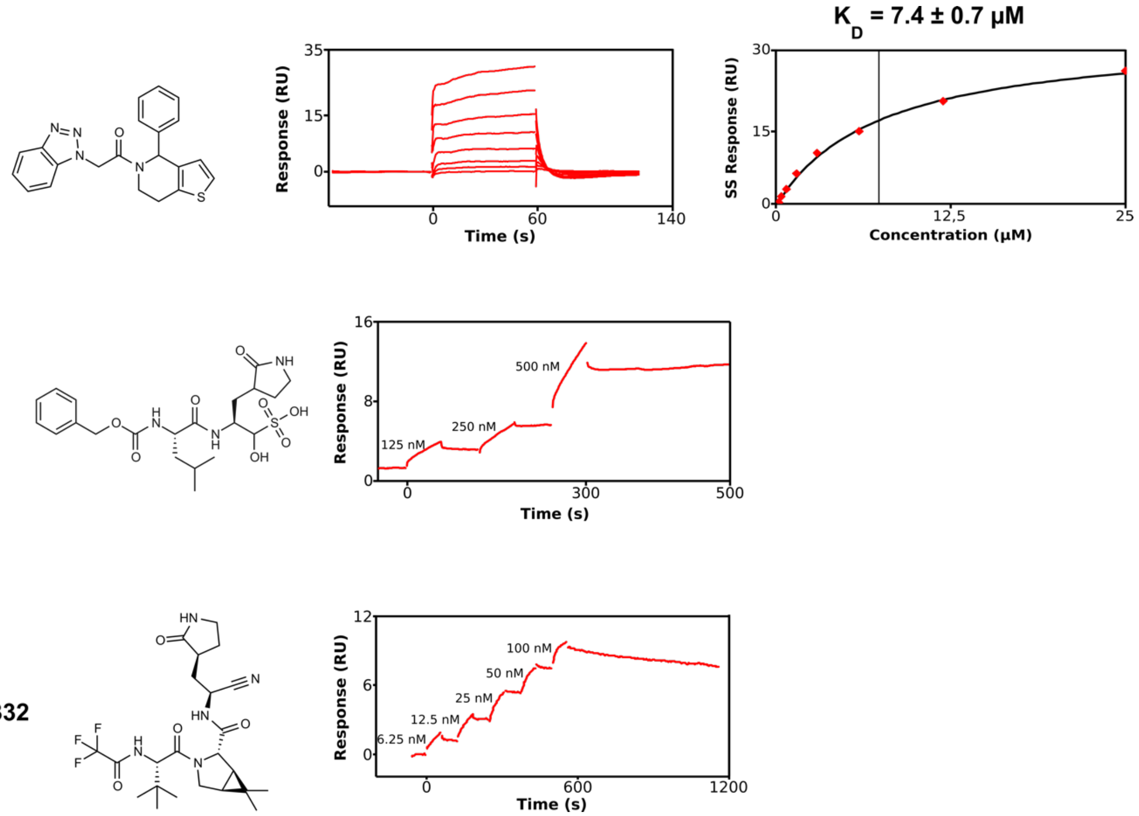

Supplementary Fig. 2. Analysis of interactions between inhibitors and M^{pro} using an SPR biosensor assay. K_D were estimated from report points at the end of the injection, assuming steady-state and a 1:1 single interaction. K_D values are reported as mean ± standard error of the fit SE (n=1). K_D values for compounds **X77**, **18** and **19** are expressed as mean ± SD from at least two independent experiments.

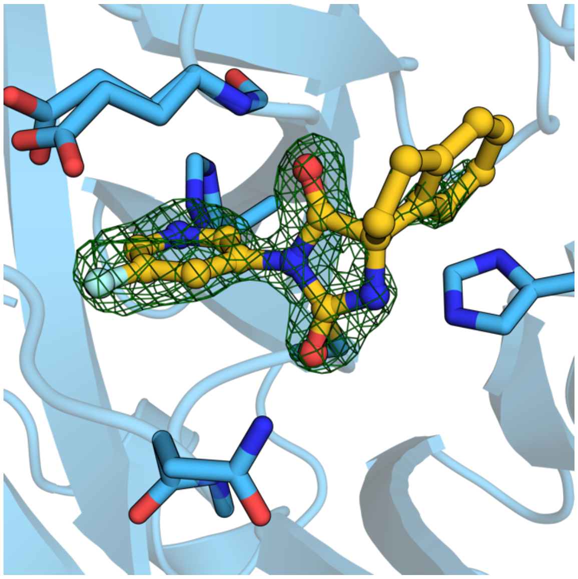

Supplementary Fig. 3. Binding mode of compound **13** with (F_o-F_c) electron density difference maps at +3σ carved at 1.5 Å from the ligand as a green isomesh (PDB accession code: 7BIJ).

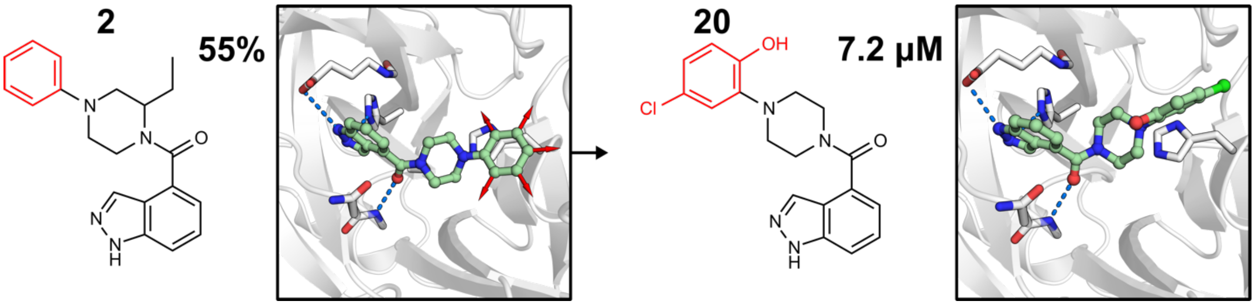

Supplementary Fig. 4. Docking pose of the scaffold of compound **2** (55% enzyme activity at 50 μM, Table 1) with growing vectors shown as arrows (left panel). Decoration of the phenyl-ring led to the discovery of compound **20** with an IC_{50} value of 7.2 μM (Supplementary Fig. 1). The docking pose of compound **20** in M^{pro} is also shown (right panel).

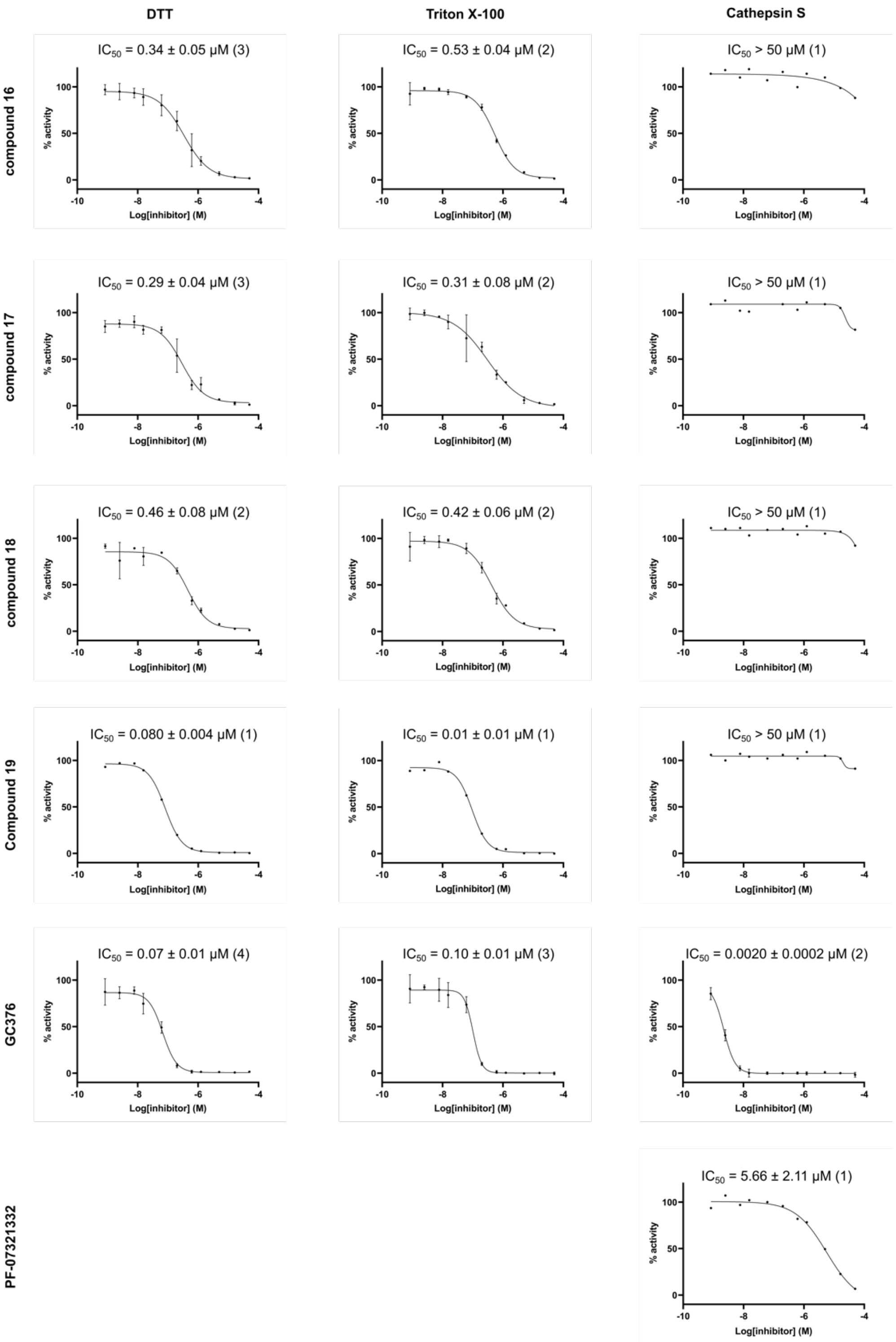

Supplementary Fig. 5. Summary of counter screens for compounds **16-19**, **GC376** and **PF-07321332**. Enzyme inhibition assays for M^{pro} were performed in the presence of either DTT or Triton X-100 (0.01%). Enzyme inhibition assay for human cathepsin S were also carried out for the same compounds. Data points represent mean ± SD from 1-3 independent experiments. IC_{50} values are expressed as mean ± SEM from (n>1) independent experiments or mean ± standard error of the fit (n=1). Note that the data for **GC376** and **PF-07321332** in these steady-state assays are not directly comparable to those for the other compounds due to their covalent mechanisms.

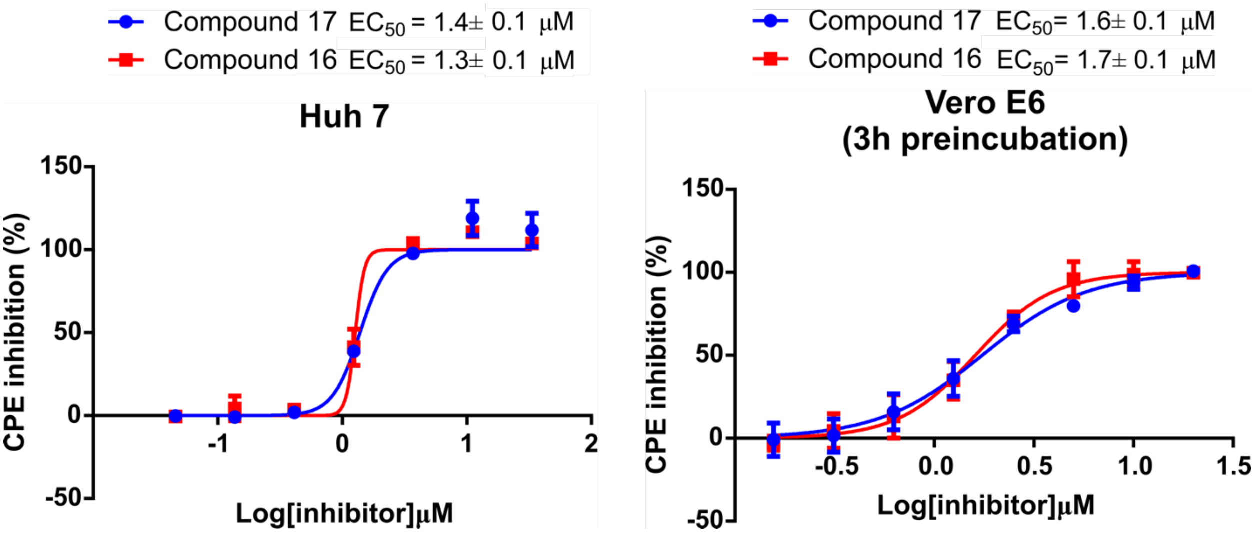

Supplementary Fig. 6. Inhibitory effect of compounds **16** and **17** on CPE induced by SARS-CoV-2 infection in Huh7 cells and Vero E6 cells (72h post infection, assay performed with a 3h preincubation step). EC_{50} values are expressed as mean ± SEM from two independent experiments. CPE inhibition in infected Vero E6 cells without pre-incubation is shown in Supplementary Fig. 8.

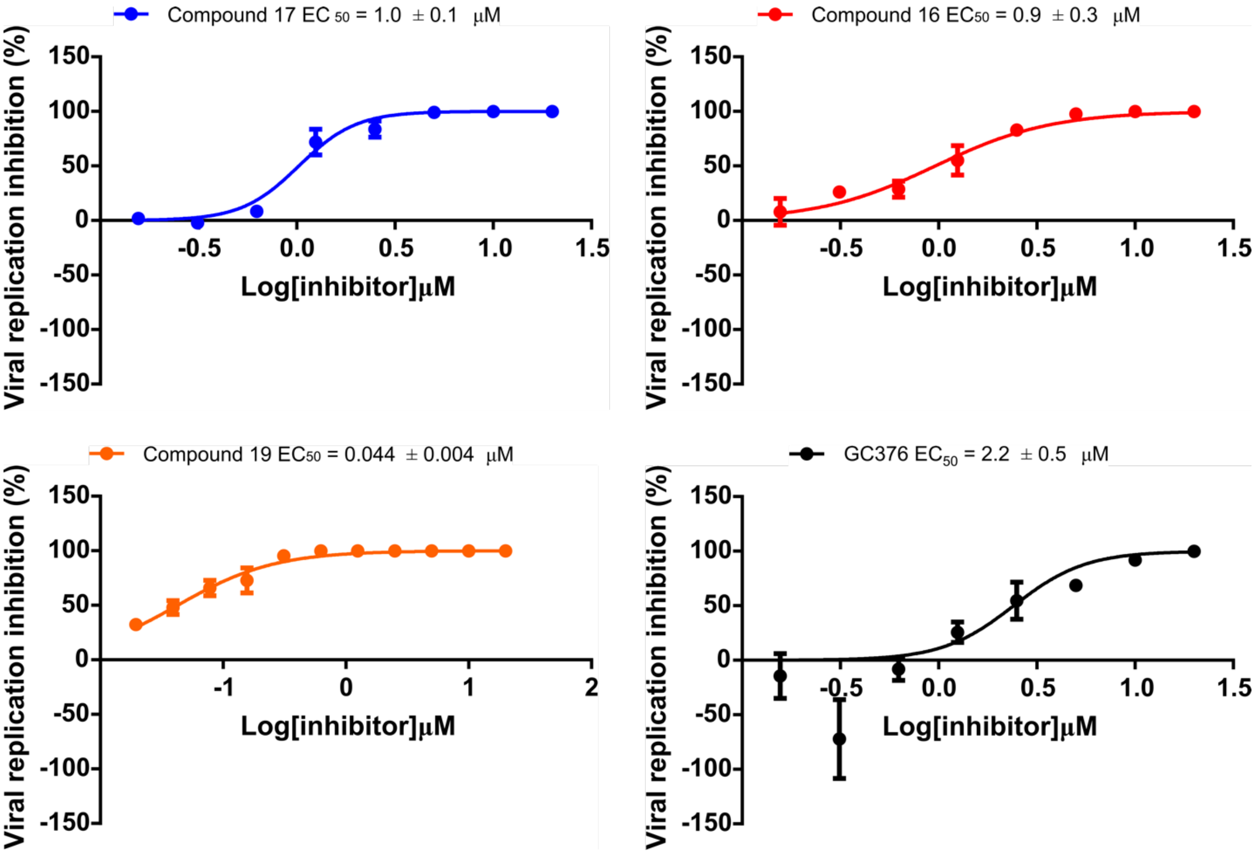

Supplementary Fig. 7. Inhibitory effect of compounds **16**, **17**, **19** and **GC376** on viral replication induced by SARS-CoV-2 infection in Vero E6 cells (RT-qPCR assay). EC_{50} values are expressed as mean ± SD from one experiment performed with triplicates.

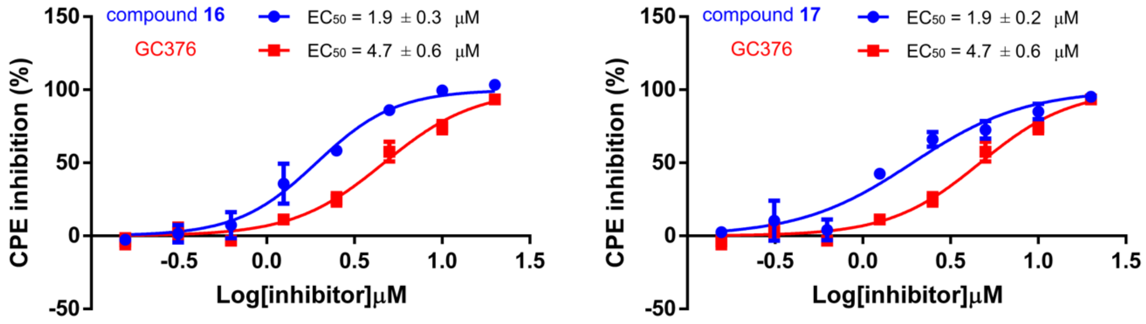

Supplementary Fig. 8. Inhibitory effect of compounds **16** and **17** on CPE induced by SARS-CoV-2 infection in Vero E6 cells without incubation. EC_{50} values are expressed as mean ± SEM from two independent experiments.

General Synthetic Procedures. All reagents were purchased from Fluorochem, Sigma-Aldrich, Enamine and Chemtronica. DCM, methanol, DMF, and acetonitrile (99.9%) were purchased from VWR International AB, whereas THF was purchased from Sigma-Aldrich. Reagents and solvents were used as such without further purification. All reactions involving air, or moisture-sensitive reagents or intermediates, were performed under a nitrogen atmosphere. LC-MS was used for monitoring reactions and assessing purity using an Agilent 1100 series HPLC having a C18 Atlantis T3 column (3.0 × 50 mm, 5 μm). Acetonitrile–water (both containing 0.1% HCOOH, flow rate 0.75 ml/min, and with a gradient of 5-95% acetonitrile over 6 min) was used as mobile phase. A Waters micromass Z_Q (model code: MM1) mass spectrometer with electrospray ionization was used for detection of molecular ions. Silica gel 60 F_{254} TLC plates from Merck were also sometimes used for monitoring reactions and particularly during purification of compounds. Visualization of the developed TLC was done using UV light (254 nm) and staining with ninhydrin or anisaldehyde. After workup, organic phases were dried over Na_2SO_4/$MgSO_4$ and filtered before being concentrated under reduced pressure. Silica gel (Matrex, 60 Å, 35–70 μm, Grace Amicon) was used for purification of intermediate compounds with flash column chromatography. Preparative reversed-phase HPLC was performed on a Kromasil C8 column (250 × 21.2 mm, 5 μm) on a Gilson HPLC equipped with Gilson 322 pump, UV/Visible-156 detector and 202 collector using acetonitrile-water gradients as eluents with a flow rate of 15 ml/min and detection at 210 or 254 nm. Unless otherwise stated, all the tested compounds were purified by HPLC. ^{1}H and ^{13}C NMR spectra for the synthesized compounds were recorded at 298 K on an Agilent Technologies 400 MR spectrometer at 400 MHz or 100 MHz, or on Bruker Avance Neo spectrometers at 500/600 MHz or 125/150 MHz. Chemical shifts are reported in parts per million (ppm, δ) referenced to the residual ^{1}H resonance of the solvent [$(CD_3)_2CO$, δ 2.05; $CDCl_3$, δ 7.26;

CD$_3$OD δ 3.31; DMSO-d_6 δ 2.50]. Splitting patterns are designated as follows: s (singlet), d (doublet), t (triplet), m (multiplet) and br (broad). Coupling constants (*J* values) are listed in hertz (Hz). The purity of the tested compounds **10, 11, 12, 16, 17, 18, 19, 22, 23, 24** and **25** is $\geq$95% as determined by high resolution ^{1}H NMR spectroscopy (600 MHz) and LCMS.

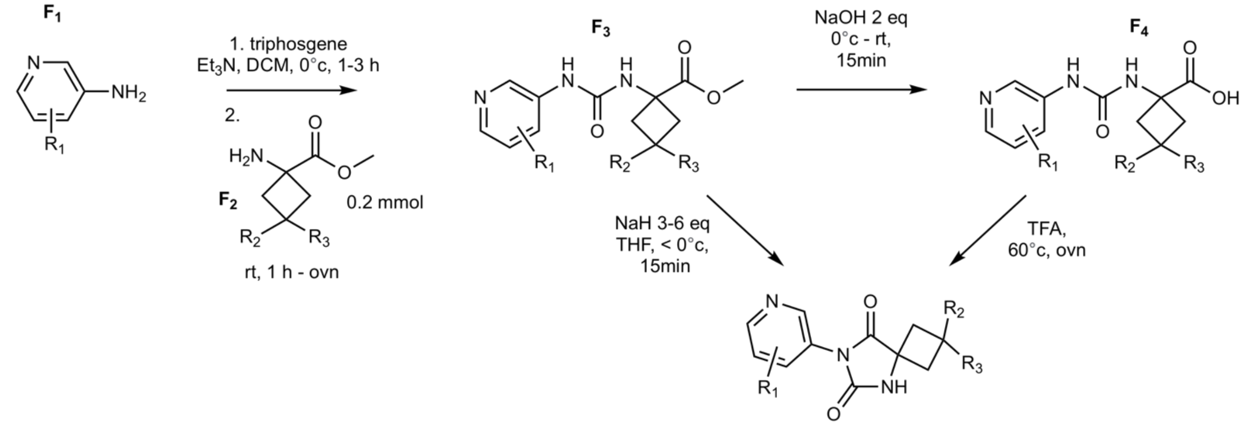

Scheme S1: General strategy for synthesis of hydantoin analogues

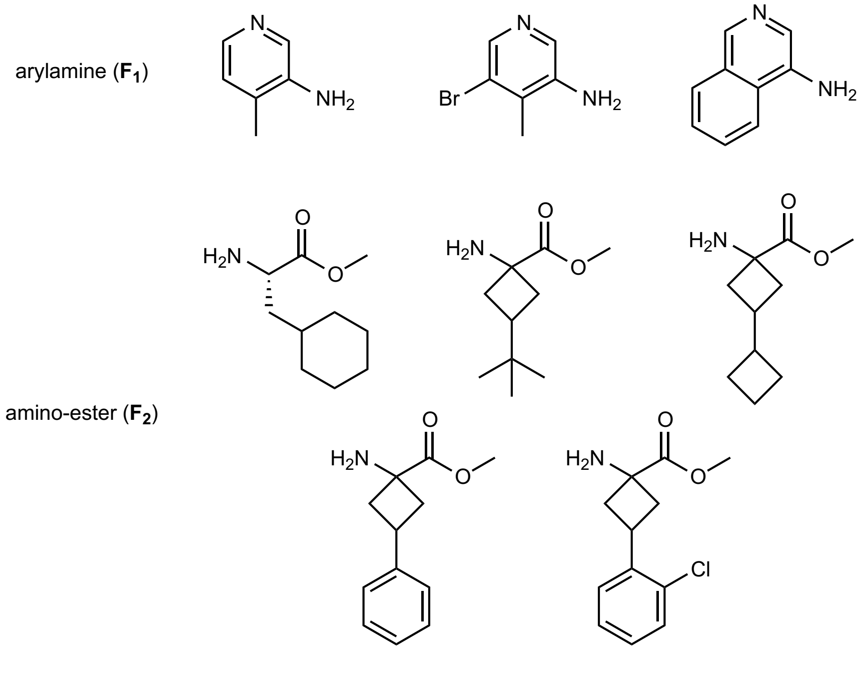

Scheme S2: Building blocks for synthesis of hydantoin analogues

General synthetic description

Triphosgene mediated formation of isocyanates from aryl amine derivatives **F_1**, followed by addition of amino acid ester **F_2** afforded the key urea ester intermediates **F_3**[1]. Hydantoin analogues could be prepared directly by cyclization of urea ester intermediates **F_3** under basic condition using NaH[1]. Alternatively, hydrolysis of esters **F_3** afforded urea acid intermediates **F_4** which were cyclized under acidic condition using TFA to afford hydantoin analogues[2].

General procedure for synthesis of urea ester intermediates F_3, modified from[1]

Et_3N (3 equiv) was added to a mixture of the aromatic amine derivative (**F_1**, 0.25 mmol, 1 equiv) in DCM (2 ml) at 0 ^oC. A solution of triphosgene (0.5 equiv) in DCM (0.5 ml) was added dropwise to the mixture. The reaction was stirred at 0 ^oC for 45 min. Then, amino acid ester hydrochloride (**F_2**, 0.2 mmol) was added to the reaction mixture in one portion. The mixture was stirred overnight at rt, then diluted with DCM (15 ml) and washed with brine. The organic phase was dried over Na_2SO_4, filtered and concentrated to give crude urea ester intermediate **F_3**, which was used as such for the next step without purification.

General procedure for cyclization of urea ester intermediates F_3 to form hydantoin analogues using NaH[1]

NaH (3-6 equiv) was added to a mixture of urea ester intermediates **F_3** in THF (2 ml) at 0 ^oC. The mixture was stirred at 0 ^oC for 15 min and then neutralized with TFA. The solvent was removed and the residue was dissolved in DMSO, filtered and purified by HPLC using 5-100% of CH_3CN in H_2O to afford the desired product as slightly yellow solid. Repurification by HPLC using 5-100% of CH_3CN in H_2O (H_2O + 0.1% TFA) afforded pure compounds as solid TFA salts.

General 2-step procedure for cyclization of urea ester intermediates F_3 to form hydantoin analogues using TFA[2]

NaOH (2 equiv) was added to a mixture of urea ester intermediates $\mathbf{F_3}$ in MeOH (2 ml) at 0 °C. The mixture was stirred at rt for 15 min, after which LCMS showed complete ester hydrolysis to acid intermediate $\mathbf{F_4}$ and partial cyclization. The reaction mixture was neutralized with TFA, then concentrated to dryness. The residue was dissolved in TFA (2 ml) and heated overnight at 60 °C. Then the solution was cooled and concentrated to dryness. The residue was dissolved in DMSO, filtered and purified by HPLC using 5-100% of CH_3CN in H_2O to afford the desired product as slightly yellow solid. Repurification by HPLC using 5-100% of CH_3CN in H_2O (H_2O + 0.1% TFA) afforded pure compounds as solid TFA salts.

General procedure for synthesis of reference compounds

HR-MS analysis was conducted using an Orbitrap mass analyzer (LTQ-Velos Pro, Thermo Fisher), loaded with an Agilent 1100 Autosampler (direct injection, methanol).

ML188: To a solution of 2-furoic acid (0.100 g, 0.89 mmol) in methanol (4 ml) was added sequentially 4-*tert*-butyl aniline (0.133 g, 142 µl, 0.89 mmol), 3-pyridine carboxaldehyde (0.096 g, 84 µl, 0.89 mmol) and *tert*-butyl isocyanide (0.074 g, 101 µl, 0.89 mmol). The mixture was sealed and stirred at room temperature for 16 hours. At completion, the vessel was opened and heated to 50 °C. After concentration of the mixture to dryness, purification was performed by silica gel column chromatography (EtOAc: isohexanes, 2:3 to 4:1) to afford the title compound as a white solid (racemic mixture).

X77: To a solution of 4-imidazolecarboxylic acid (0.51 g, 0.45 mmol) in methanol (2 ml) was added sequentially 4-*tert*-butyl aniline (0.067 g, 72 µl, 0.45 mmol), 3-pyridine carboxaldehyde (0.048 g, 42 µl, 0.45 mmol) and cyclohexyl isocyanide (0.049 g, 56 µl, 0.45 mmol). The mixture was sealed and stirred at 40 °C for 16 hours. At completion, the vessel was opened and heated to 50 °C. After concentration of the mixture to dryness, purification was performed by silica gel column chromatography (MeOH: DCM, 3:97 to 1:9) to afford the title compound as a white solid (racemic mixture).

Preparative stereoisomeric separation of compound 16

After purification by reversed phase chromatography the stereoisomers of compound **16** were separated by preparative supercritical fluid chromatography (SFC). The sample for chiral separation was prepared by dissolving 15 mg of the isomeric mixture in 0.75 mL of MeOH and the preparative run was performed by stacked injections on a SFC system connected to a PDA detector. The column used was a 5 µm, YMC Chiral Cellulose-SB, 10 mm × 250 mm (diameter x length) and the column temperature was set to 45 °C. An isocratic condition of 80% CO_2 and 20% MeOH was applied at a flow rate of 15 mL/min. The back pressure was set to 120 Bar. The PDA scanned from 220 to 400 and the stereoisomers were collected in separate fractions (with the aid of 2 mL/min of MeOH as make up solvent for the collection) and pooled from each injection.

Preparative stereoisomeric separation of compound 17

After purification by reversed phase chromatography the stereoisomers of compound **17** were separated by preparative supercritical fluid chromatography (SFC). The sample for chiral separation was prepared by dissolving 15 mg of the isomeric mixture in 0.75 mL of MeOH and the preparative run was performed by stacked injections on a SFC system connected to a PDA detector. The column used was a 5 µm, YMC Chiral Cellulose-SB, 10 mm × 250 mm (diameter x length) and the column temperature was set to 45 °C. An isocratic condition of 85% CO_2 and 15% MeOH was applied at a flow rate of 15 mL/min. The back pressure was set to 120 Bar. The PDA scanned from 220 to 400 and the stereoisomers were collected in separate fractions (with the aid of 2 mL/min of MeOH as make up solvent for the collection) and pooled from each injection.

Structural elucidation of stereoisomers via NOESY NMR

The structures of compound **16a**, **16b**, **17a** and **17b** were assigned based on 1D and 2D NMR data. Spectra for these compounds are shown below.

The assignment of the relative configuration of the compounds at C-7 was deduced from the NOESY spectra. In the NOESY spectra of compounds **16a** and **17a** missing a cross peak between H-21 and H-26 indicates that the two protons are oriented in anti-position. In the NOESY spectra of **16b** and **17b** a cross peak between H-22 and H-26 was observed, revealing that the two protons are oriented in syn-position. From these observations, the relative configuration of the compounds was determined.

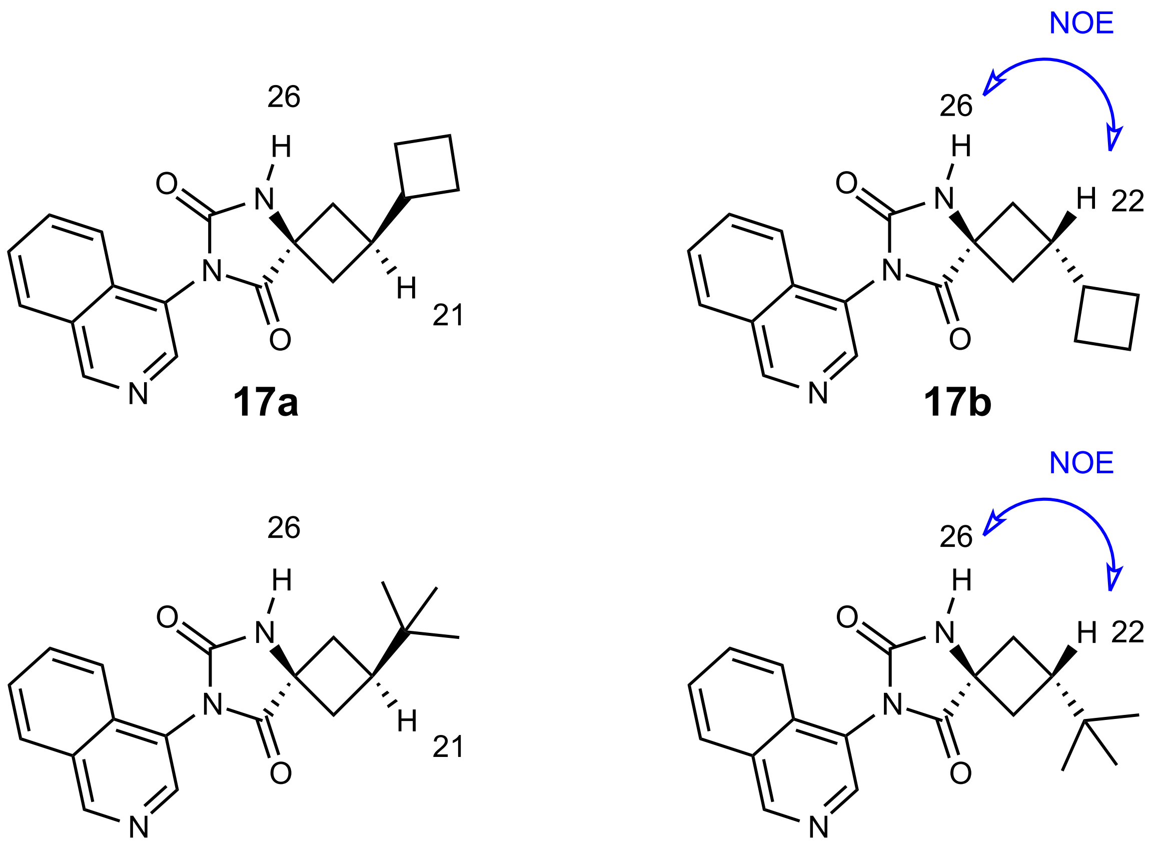

17a **17b**

16a **16b**

Supplementary Fig. 9. Relative configuration of stereoisomers elucidated via NOESY NMR.

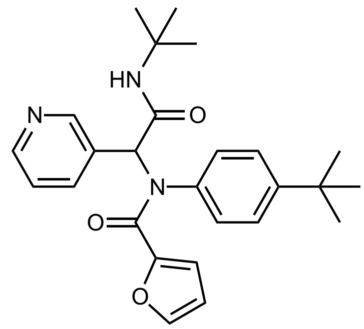

ML188 Racemic mixture.

Yield: 0.236 g (61%).

HRMS (ESI-MS): calculated for $C_{26}H_{31}N_3O_3$ $(M+H)^+$: 434.2434; found: 434.2439.

^{1}H NMR (400 MHz, $CDCl_3$) δ 8.46-8.43 (m, 2H), 7.50 (dt, *J* = 8.0, 1.7 Hz, 1H), 7.36-7.36 (m, 1H), 7.23 (d, *J* = 8.7 Hz, 2H), 7.05 (dd, *J* = 7.9, 4.8 Hz, 1H), 6.97 (br s, 2H), 6.27 (br s, 1H), 6.13 (dd, *J* = 3.6, 1.7 Hz, 1H), 6.10 (s, 1H), 5.36 (d, *J* = 3.6 Hz, 1H), 1.34 (s, 9H), 1.25 (s, 9H).

^{13}C NMR (101 MHz, $CDCl_3$) δ 167.8, 159.6, 152.5, 151.3, 149.3, 146.2, 144.9, 138.3, 136.5, 130.6, 130.2, 126.1, 122.9, 117.1, 111.2, 63.6, 51.8, 34.7, 31.3, 28.6.

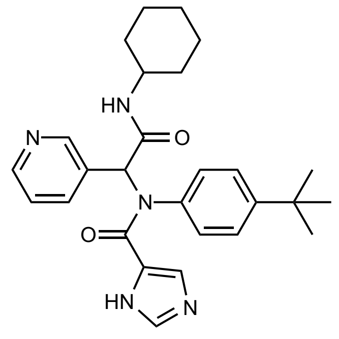

X77 Racemic mixture.

Yield: 0.091 g (44%).

HRMS (ESI-MS): calculated for $C_{27}H_{33}N_5O_2$ $(M+H)^+$: 460.2707; found: 460.2707.

^{1}H NMR (400 MHz, $CDCl_3$) δ 8.27 (br s, 1H), 8.23 (dd, *J* = 4.9, 1.3 Hz, 1H), 7.53 (s, 1H), 7.50 (dt, *J* = 8.0, 2.1 Hz, 1H), 7.21 (d, *J* = 7.3 Hz, 2H), 7.12 (dd, *J* = 7.9, 4.9 Hz, 1H), 6.18 (s, 1H), 5.35 (s, 1H), 3.63-3.57 (m, 1H), 1.80 (d, *J* = 11.9 Hz, 1H), 1.66-1.62 (m, 2H), 1.58-1.48 (m, 2H), 1.31-1.19 (m, 2H), 1.16 (s, 9H), 1.14-0.93 (m, 3H).

^{13}C NMR (101 MHz, $CDCl_3$) δ 171.3, 163.8, 154.8, 152.8, 150.5, 141.1, 138.7, 138.6, 133.8, 133.3, 130.2, 127.9, 125.6, 65.0, 51.2, 36.4, 34.41, 34.37, 32.5, 27.4, 26.95, 26.86.

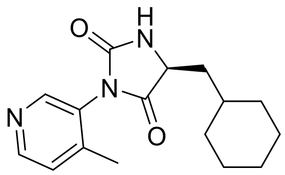

Compound 10 Stereoisomeric mixture. Hydantoin prepared using the TFA procedure. Yield: 20 mg (35%).

LCMS (ESI+): calculated for $C_{16}H_{22}N_3O_2$ $(M+H)^+$: 288.2; found: 288.3.

^{1}H NMR (600 MHz, $CDCl_3$) δ 8.72-8.48 (m, 2H), 7.50-7.41 (m, 1H), 7.03 (br s, 1H, dias), 6.78 (br s, 1H, dias), 2.34 (s, 3H), 1.96-0.91 (m, 13H).

^{13}C NMR (150 MHz, $CDCl_3$) δ 172.9, 155.1, 149.5, 149.0, 147.0, 146.8, 146.3, 128.9, 128.8, 126.7, 126.6, 55.8, 55.7, 39.5, 34.3, 33.9, 33.7, 32.3, 32.2, 26.2, 26.0, 25.8, 18.2.

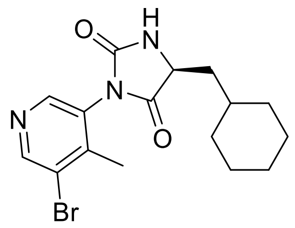

Compound 11 Stereoisomeric mixture. Hydantoin prepared using the TFA procedure.

Yield: 31 mg (42%).

LCMS (ESI+): calculated for $C_{16}H_{21}BrN_3O_2$ $(M+H)^+$: 366.1; found: 366.2.

^{1}H NMR (600 MHz, $CDCl_3$) δ 8.75 (s, 1H, dias), 8.74 (s, 1H, dias), 8.51 (s, 1H, dias), 8.42 (s, 1H, dias), 6.72 (br s, 1H, dias), 6.55 (br s, 1H, dias), 4.31-4.36 (m, 1H), 2.34 (s, 3H, dias), 2.32 (s, 3H, dias), 1.94-0.94 (m, 13H).

^{13}C NMR (150 MHz, $CDCl_3$) δ 172.7, 155.0, 150.1, 149.6, 148.2, 147.9, 146.4, 146.3, 128.9, 128.8, 124.3, 55.9, 55.7, 40.0, 39.5, 34.4, 33.8, 32.3, 32.2, 26.1, 26.0, 25.8, 18.6, 18.5.

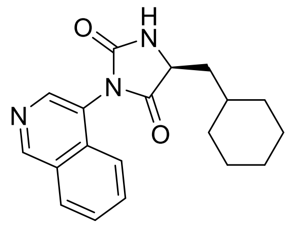

Compound 12 Stereoisomeric mixture as TFA salt. Hydantoin prepared using the TFA procedure.

Yield: 20 mg (31%).

LCMS (ESI+): calculated for $C_{19}H_{11}N_3O_2$ $(M+H)^+$: 324.2; found: 324.3.

^{1}H NMR (600 MHz, DMSO-d6) δ 9.47 (s, 1H), 8.81 (s, 1H, dias), 8.78 (s, 1H, dias), 8.50 (s, 1H, dias), 8.47 (s, 1H, dias), 8.31-8.28 (m, 1H), 7.95-7.61 (m, 3H), 4.59-4.40 (m, 1H), 1.96-0.83 (m, 13H).

^{13}C NMR (150 MHz, DMSO-d6) δ 174.4, 174.3, 155.5, 153.0, 152.9, 142.4, 142.1, 132.7, 132.4, 131.9, 131.7, 128.4, 128.2, 128.1, 124.2, 124.0, 122.1, 121.3, 33.3, 33.2, 33.1, 31.6, 25.8, 25.6, 25.4.

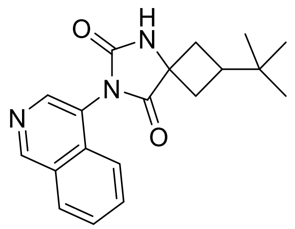

Compound 16 Stereoisomeric mixture as TFA salt. Hydantoin prepared using the TFA procedure. Yield: 21 mg (30%).

LCMS (ESI+): calculated for $C_{19}H_{22}N_3O_2$ $(M+H)^+$: 324.2; found: 324.3.

^{1}H NMR (600 MHz, CD_3OD) δ 9.54 (s, 1H), 8.56 (d, *J* = 8.7 Hz, 1H), 8.37 (d, *J* = 8.3 Hz, 1H), 8.05-8.01 (m, 1H), 7.90 (t, *J* = 7.5 Hz, 1H), 7.85-7.81 (m, 1H), 2.75-2.23 (m, 5H), 0.93 (s, 9H. dias), 0.92 (s, 9H, dias).

^{13}C NMR (150 MHz, CD_3OD) δ 178.7, 176.9, 156.9, 156.4, 152.8, 152.7, 140.1, 140.0, 135.7, 135.6, 135.3, 130.8, 127.4, 127.2, 123.3, 123.2, 59.9, 58.2, 40.8, 38.9, 35.6, 35.1, 34.7, 34.3, 32.1, 31.9, 26.5, 26.4.

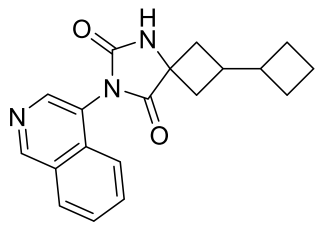

Compound 17 Stereoisomeric mixture as TFA salt. Hydantoin prepared using the TFA procedure.

Yield: 29 mg (45%).

LCMS (ESI+): calculated for $C_{19}H_{20}N_3O_2$ $(M+H)^+$: 322.2; found: 322.3.

^{1}H NMR (600 MHz, CD_3OD) δ 9.51 (s, 1H), 8.54 (d, *J* = 8.8 Hz, 1H), 8.35 (d, *J* = 8.3 Hz, 1H), 8.02-7.98 (m, 1H), 7.88 (d, *J* = 7.7 Hz, 1H), 7.82-7.78 (m, 1H), 2.80-1.69 (m, 12H).

^{13}C NMR (150 MHz, CD_3OD) δ 178.7, 177.3, 156.7, 156.4, 153.1, 153.0, 140.6, 140.5, 135.5, 135.1, 130.6, 130.4, 130.3, 127.2, 127.0, 123.2, 123.2, 60.5, 59.0, 41.7, 40.8, 37.6, 37.2, 36.8, 36.4, 33.9, 32.6, 26.2, 25.9, 18.8, 18.7.

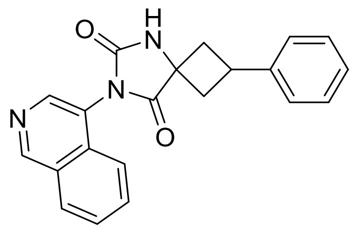

Compound 18 TFA salt. Hydantoin prepared using the TFA procedure.

Yield: 10 mg (14%).

LCMS (ESI+): calculated for $C_{21}H_{18}N_3O_2$ $(M+H)^+$: 344.1; found: 344.2.

^{1}H NMR (600 MHz, CD_3OD) δ 9.55 (s, 1H), 8.61 (s, 1H), 8.38 (d, *J* = 8.3 Hz, 1H), 8.06-8.03 (m, 1H), 7.93-7.89 (m, 2H), 7.38-7.33 (m, 4H), 7.26-7.20 (m, 1H), 3.79-3.72 (m, 1H), 3.21-3.11 (m, 2H), 2.75-2.65 (m, 2H).

^{13}C NMR (150 MHz, CD_3OD) δ 178.5, 156.4, 153.0, 145.2, 140.3, 135.7, 135.2, 130.7, 130.3, 129.7, 127.7, 127.1, 123.3, 59.1, 42.0, 41.6, 32.7.

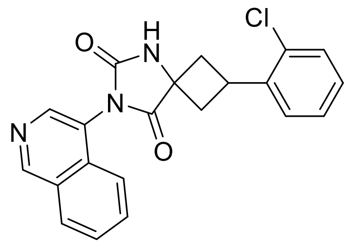

Compound 19 Single stereoisomer as TFA salt. Hydantoin prepared using the TFA procedure.

Yield: 40 mg (41%).

LCMS (ESI+): calculated for $C_{21}H_{17}ClN_3O_2$ $(M+H)^+$: 378.1; found: 378.2.

^{1}H NMR (600 MHz, DMSO-d_6) δ 9.46 (s, 1H), 9.00 (s, 1H), 8.56 (s, 1H), 8.29 (d, *J* = 8.2 Hz, 1H), 7.89 (dd, *J* = 7.3, 0.4 Hz, 1H), 7.80 (t, *J* = 7.5 Hz, 1H), 7.77 (d, *J* = 8.4 Hz, 1H), 7.52 (d, *J* = 7.4 Hz, 1H), 7.45 (d, *J* = 8.0 Hz, 1H), 7.43 (t, *J* = 7.5 Hz, 1H), 7.32-7.26 (m, 1H), 3.89-3.74 (m, 1H), 3.19-3.06 (m, 2H), 2.64-2.59 (m, 2H).

^{13}C NMR (150 MHz, DMSO-d_6) δ 176.4, 154.1, 153.2, 142.9, 140.5, 132.5, 132.4, 131.7, 129.2, 128.4, 128.1, 128.1, 128.0, 127.4, 127.3, 123.6, 121.6, 56.9, 38.7, 28.7.

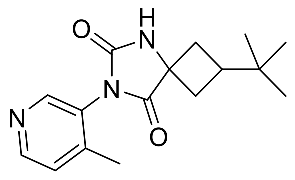

Compound 22 Stereoisomeric mixture. Hydantoin prepared using the NaH procedure.

Yield: 20 mg (35%).

LCMS (ESI+): calculated for $C_{16}H_{22}N_3O_2$ $(M+H)^+$: 288.2; found: 288.3.

^{1}H NMR (600 MHz, $CDCl_3$) δ 8.52 (t, *J* = 5.0 Hz, 1H), 8.45 (d, *J* = 4.0 Hz, 1H), 7.30 (t, *J* = 4.6 Hz, 1H), 6.60 (br s, 1H, dias), 6.18 (br s, 1H, dias), 2.78-2.11 (m, 8H), 0.89 (s, 9H, dias), 0.86 (s, 9H, dias).

^{13}C NMR (150 MHz, CD_3OD) δ 175.6, 173.9, 154.9, 154.4, 149.1, 149.0, 148.8, 148.7, 146.5, 128.1, 128.0, 125.9, 58.4, 56.6, 39.8, 37.3, 34.9, 34.4, 33.7, 33.2, 31.2, 31.0, 26.0, 25.9, 17.7, 17.6.

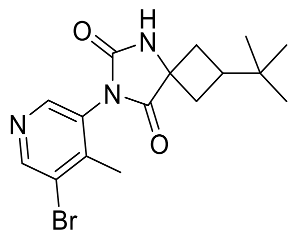

Compound 23 Stereoisomeric mixture. Hydantoin prepared using the NaH procedure. Yield: 10 mg (13%).

LCMS (ESI+): calculated for $C_{16}H_{21}BrN_3O_2$ $(M+H)^+$: 366.1; found: 366.2.

^{1}H NMR (600 MHz, $CDCl_3$) δ 8.74 (s, 1H), 8.49 (s, 1H), 7.10 (s, 1H, dias), 6.66 (s, 1H, dias), 2.73-2.66 (m, 1H), 2.59-2.42 (m, 2H), 2.31 (s, 3H, dias). 2.29 (s, 3H, dias), 2.23-2.15 (m, 2H), 0.89 (s, 9H, dias), 0.87 (s, 9H, dias).

^{13}C NMR (150 MHz, $CDCl_3$) δ 175.4, 173.6, 154.4, 153.9, 149.8, 149.6, 148.1, 146.4, 146.3, 129.1, 129.0, 124.4, 58.5, 56.8, 39.7, 37.4, 34.9, 33.8, 33.2, 31.2, 31.0, 26.0, 25.9, 18.6, 18.5.

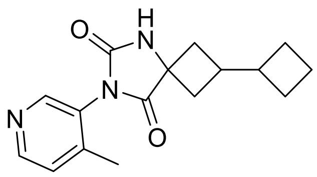

Compound 24 Stereoisomeric mixture. Hydantoin prepared using the NaH procedure.

Yield: 9 mg (16%).

LCMS (ESI+): calculated for $C_{16}H_{20}N_3O_2$ $(M+H)^+$: 286.2; found: 286.3.

^{1}H NMR (600 MHz, $CDCl_3$) δ 8.54-8.42 (m, 2H), 7.33-7.26 (m, 1H), 7.22 (br s, 1H, dias), 6.85 (br s, 1H, dias), 2.72-2.29 (m, 5H), 2.26 (s, 3H, dias), 2.24 (s, 3H, dias), 2.07-1.51 (m, 7H).

^{13}C NMR (150 MHz, $CDCl_3$) δ 175.7, 174.5, 154.9, 154.5, 148.6, 148.5, 148.4, 147.1, 128.3, 128.2, 126.1, 59.0, 57.5, 40.4, 39.1, 37.1, 36.6, 36.0, 35.5, 32.6, 31.3, 25.2, 24.9, 17.9, 17.7, 17.7.

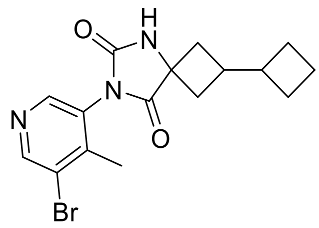

Compound 25 Stereoisomeric mixture. Hydantoin prepared using the NaH procedure. Yield: 33 mg (45%).

LCMS (ESI+): calculated for $C_{16}H_{19}BrN_3O_2$ $(M+H)^+$: 364.1; found: 364.2.

^{1}H NMR (600 MHz, $CDCl_3$) δ 8.75 (s, 1H), 8.57 (s, 1H), 7.35 (br s, 1H, dias), 6.98 (br s, 1H, dias), 2.71-2.34 (m, 5H), 2.34 (s, 3H, dias), 2.32 (s, 3H, dias), 2.14-1.61 (m, 7H).

^{13}C NMR (150 MHz, $CDCl_3$) δ 175.3, 174.0, 154.0, 153.7, 149.4, 149.3, 148.6, 148.4, 145.6, 145.4, 129.4, 129.3, 124.4, 59.2, 57.7, 39.1, 37.1, 36.6, 36.1, 35.4, 32.6, 31.3, 25.2, 24.9, 18.9, 18.8, 17.9, 17.8.

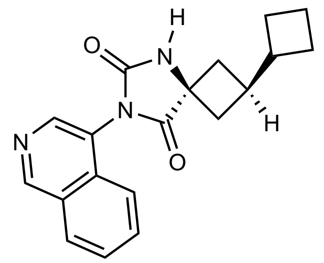

Compound 17a single stereoisomer

^{1}H NMR (601 MHz, DMSO) δ 9.43 (s, 1H), 8.98 (s, 1H), 8.50 (s, 1H), 8.27 (d, *J* = 8.1 Hz, 1H), 7.86 (t, *J* = 7.6 Hz, 2H), 7.78 (t, *J* = 7.5 Hz, 2H), 7.66 (d, *J* = 8.4 Hz, 1H), 2.66 (ddd, *J* = 11.9, 7.8, 4.7 Hz, 2H), 2.61 (ddd, *J* = 12.5, 7.9, 4.6 Hz, 2H), 2.44 (q, *J* = 8.5 Hz, 1H), 2.36 (dt, *J* = 15.6, 7.7 Hz, 2H), 2.10 (dt, *J* = 21.7, 10.4 Hz, 3H), 1.96 (qd, *J* = 8.2, 4.0 Hz, 3H), 1.87 – 1.80 (m, 1H), 1.77 (td, *J* = 9.5, 4.5 Hz, 1H), 1.68 (p, *J* = 8.9 Hz, 3H).

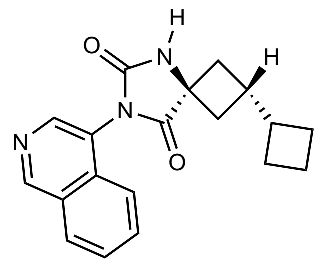

Compound 17b single stereoisomer

^{1}H NMR (601 MHz, DMSO) δ 9.44 (s, 1H), 9.26 (s, 1H), 8.50 (s, 1H), 8.28 (d, *J* = 8.2 Hz, 1H), 7.87 (dd, *J* = 8.4, 6.9 Hz, 1H), 7.79 (t, *J* = 7.5 Hz, 1H), 7.63 (d, *J* = 8.4 Hz, 1H), 2.61 – 2.52 (m, 2H), 2.50 – 2.47 (m, 1H), 2.46 – 2.37 (m, 3H), 1.97 (qd, *J* = 8.3, 4.2 Hz, 2H), 1.87 – 1.72 (m, 2H), 1.63 (p, *J* = 8.9 Hz, 2H).

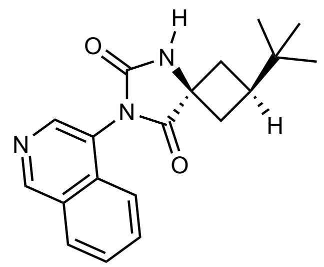

Compound 16a single stereoisomer

^{1}H NMR (601 MHz, DMSO) δ 9.44 (s, 1H), 8.98 (s, 1H), 8.51 (s, 1H), 8.28 (d, *J* = 8.2 Hz, 1H), 7.87 (dd, *J* = 8.4, 6.9 Hz, 1H), 7.79 (t, *J* = 7.5 Hz, 1H), 7.67 (d, *J* = 8.4 Hz, 1H), 2.50 (s, 6H), 2.35 – 2.18 (m, 4H), 0.86 (s, 9H).

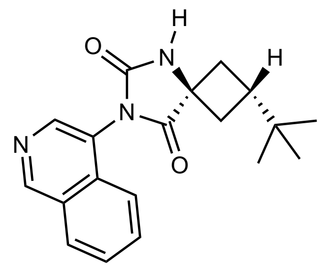

Compound 16b single stereoisomer

^{1}H NMR (601 MHz, DMSO) δ 9.43 (s, 1H), 9.33 (s, 1H), 8.48 (s, 1H), 8.27 (d, *J* = 8.2 Hz, 1H), 7.86 (dd, *J* = 8.3, 6.9 Hz, 1H), 7.78 (t, *J* = 7.5 Hz, 1H), 7.62 (d, *J* = 8.4 Hz, 1H), 2.59 – 2.51 (m, 3H), 2.23 (dq, *J* = 12.1, 5.6 Hz, 1H), 2.15 (dq, *J* = 11.7, 5.5 Hz, 1H), 0.86 (s, 9H).

NMR spectra of synthesized compounds

^{1}H NMR (600 MHz, $CDCl_3$) spectrum of compound **10**

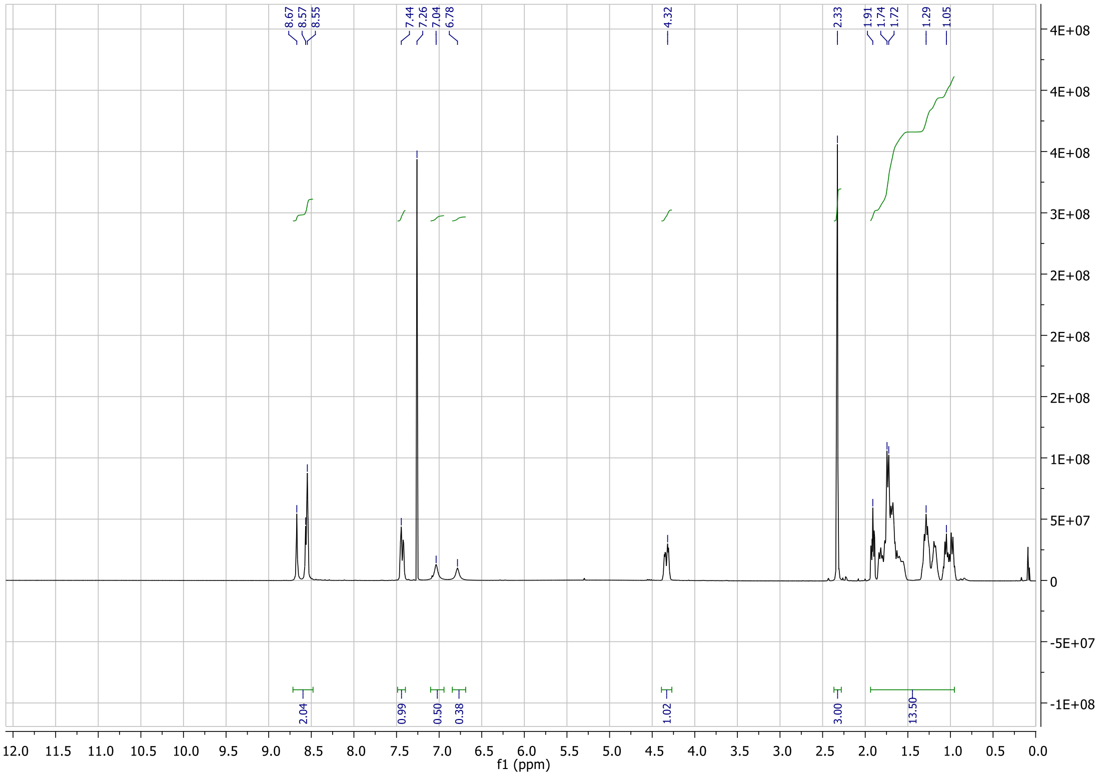

^{13}C NMR (150 MHz, $CDCl_3$) spectrum of compound **10**

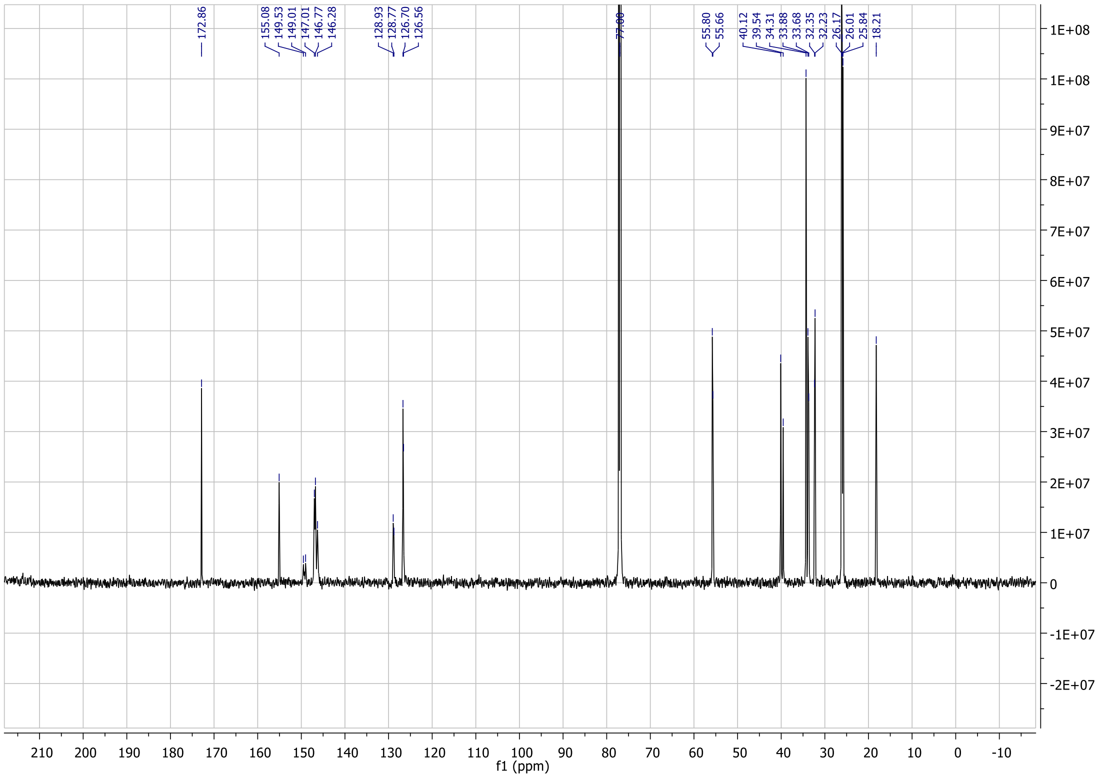

^{1}H NMR (600 MHz, $CDCl_3$) spectrum of compound **11**

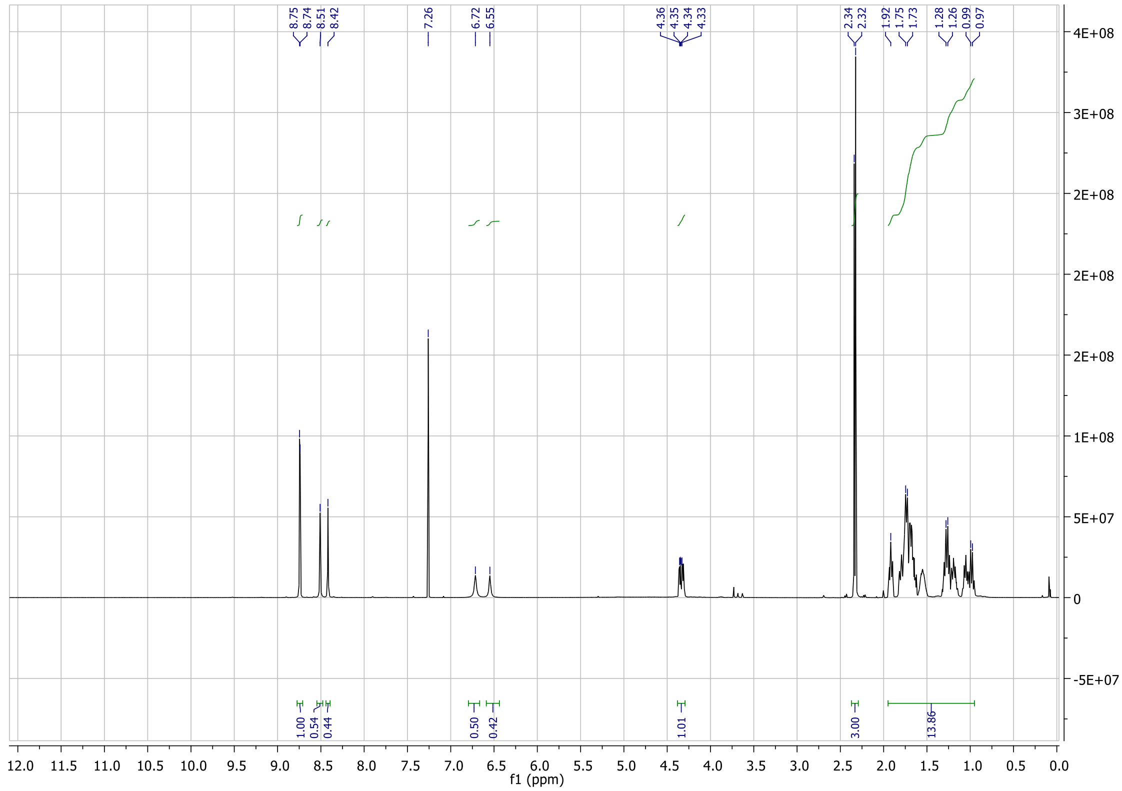

^{13}C NMR (150 MHz, $CDCl_3$) spectrum of compound **11**

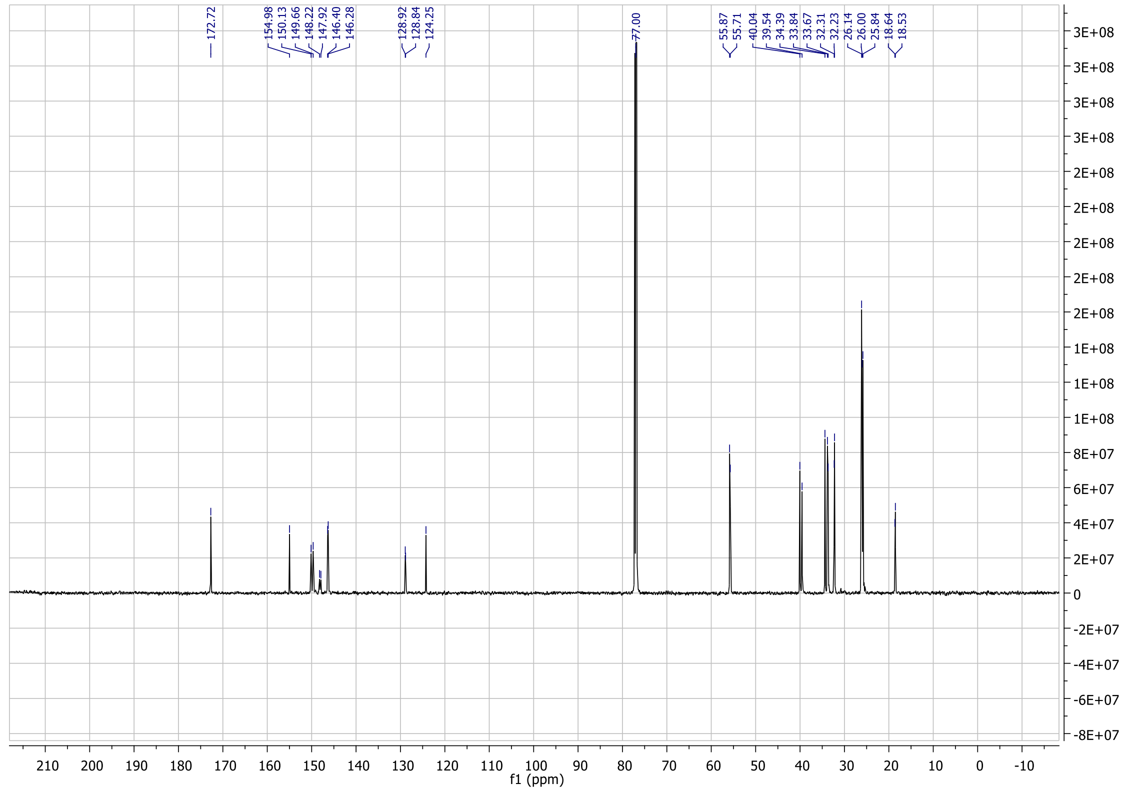

^{1}H NMR (600 MHz, DMSO-d_6) spectrum of compound **12**

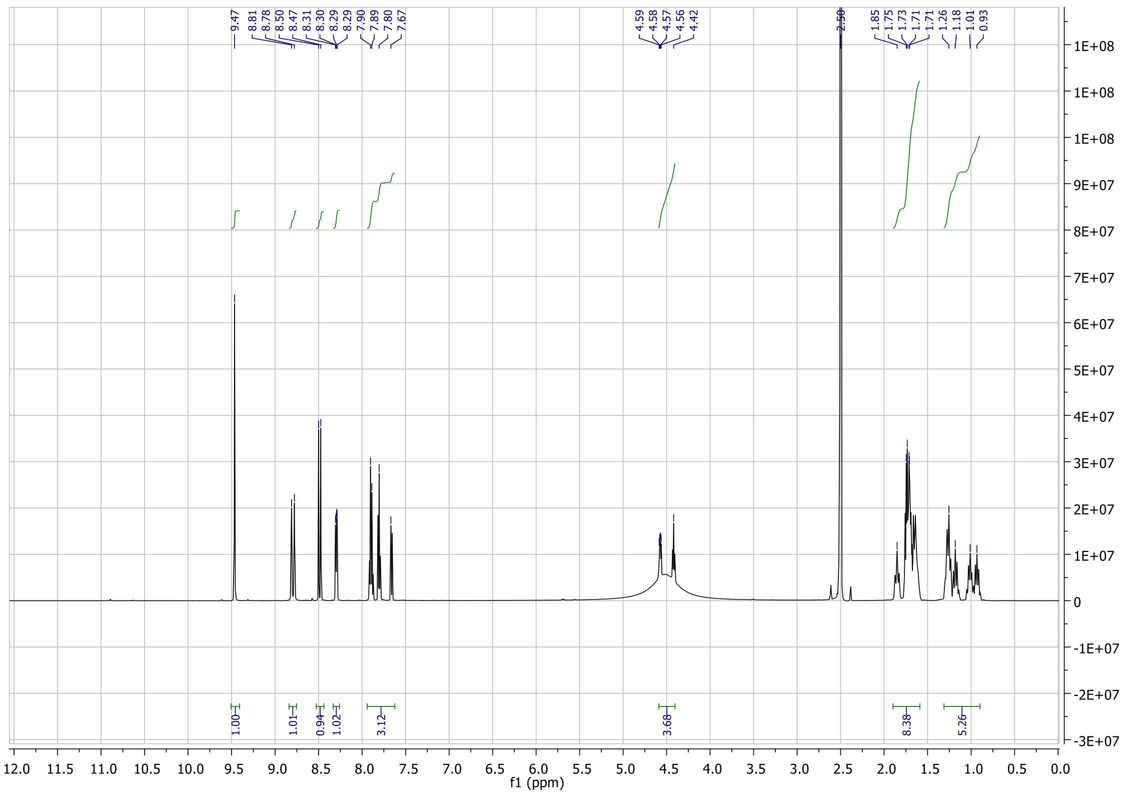

^{13}C NMR (150 MHz, DMSO-d_6) spectrum of compound **12**

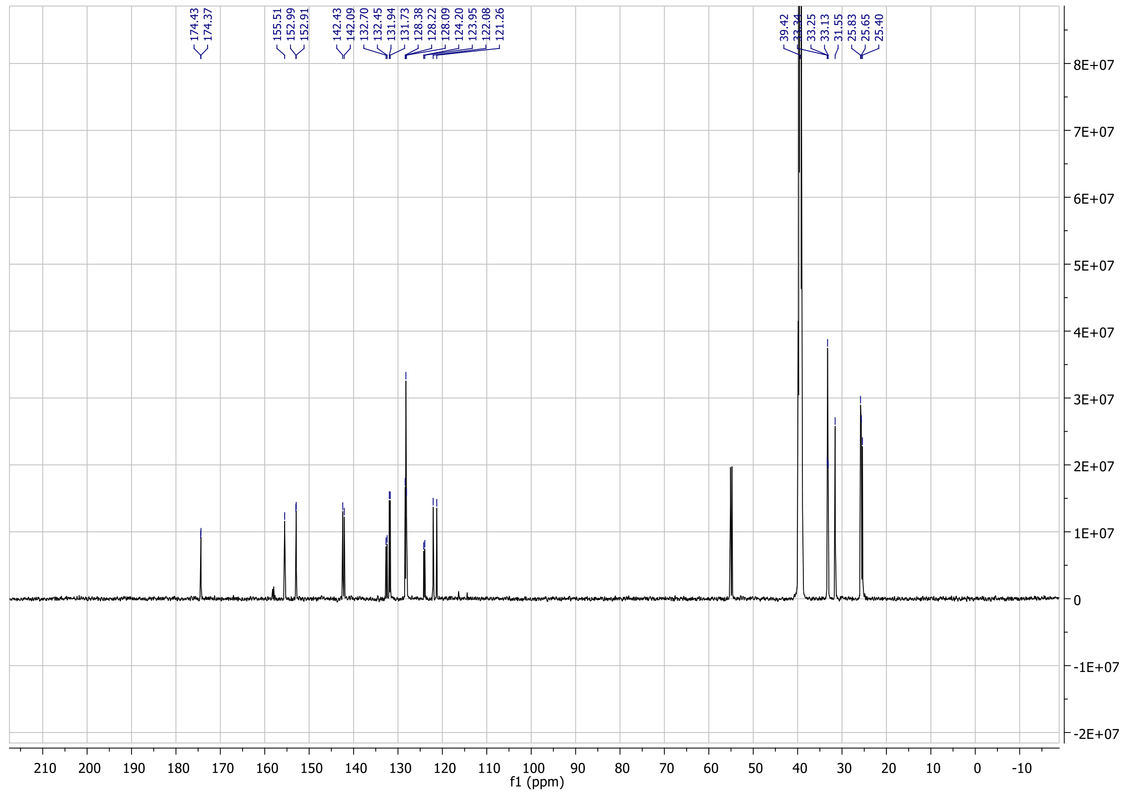

^{1}H NMR (600 MHz, CD_3OD) spectrum of compound **16**

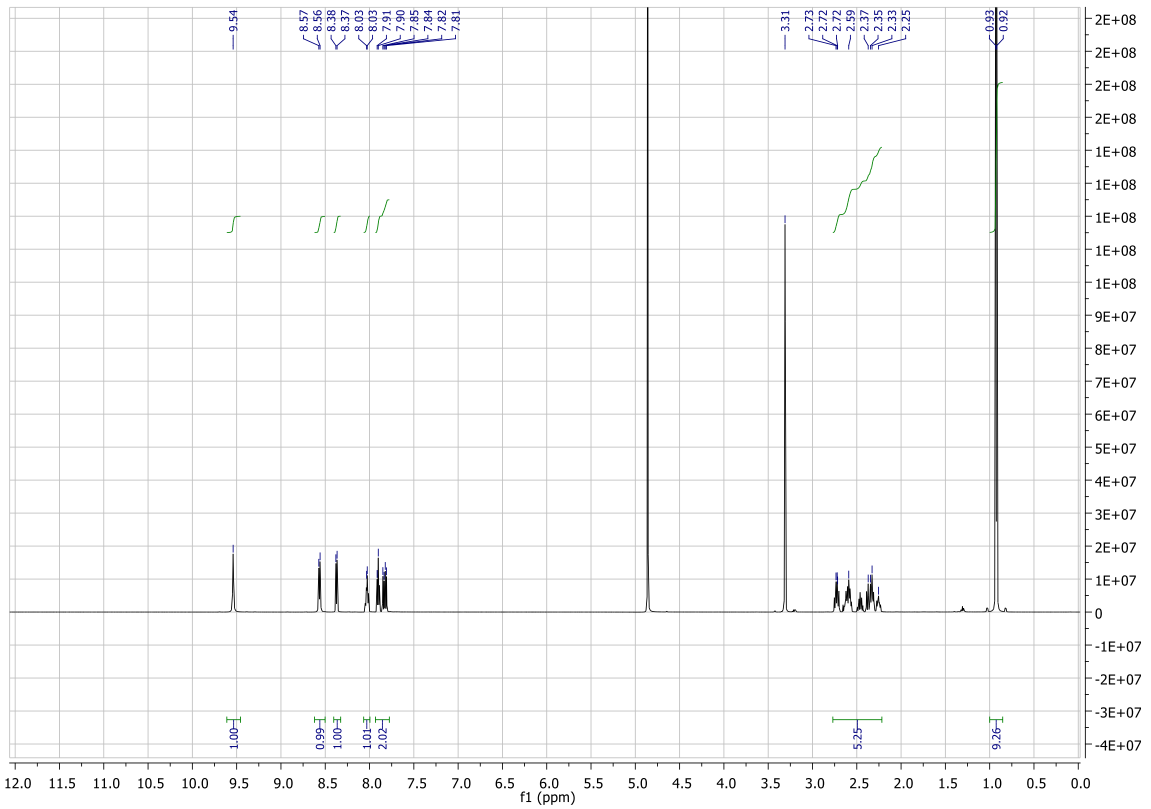

^{13}C NMR (150 MHz, CD_3OD) spectrum of compound **16**

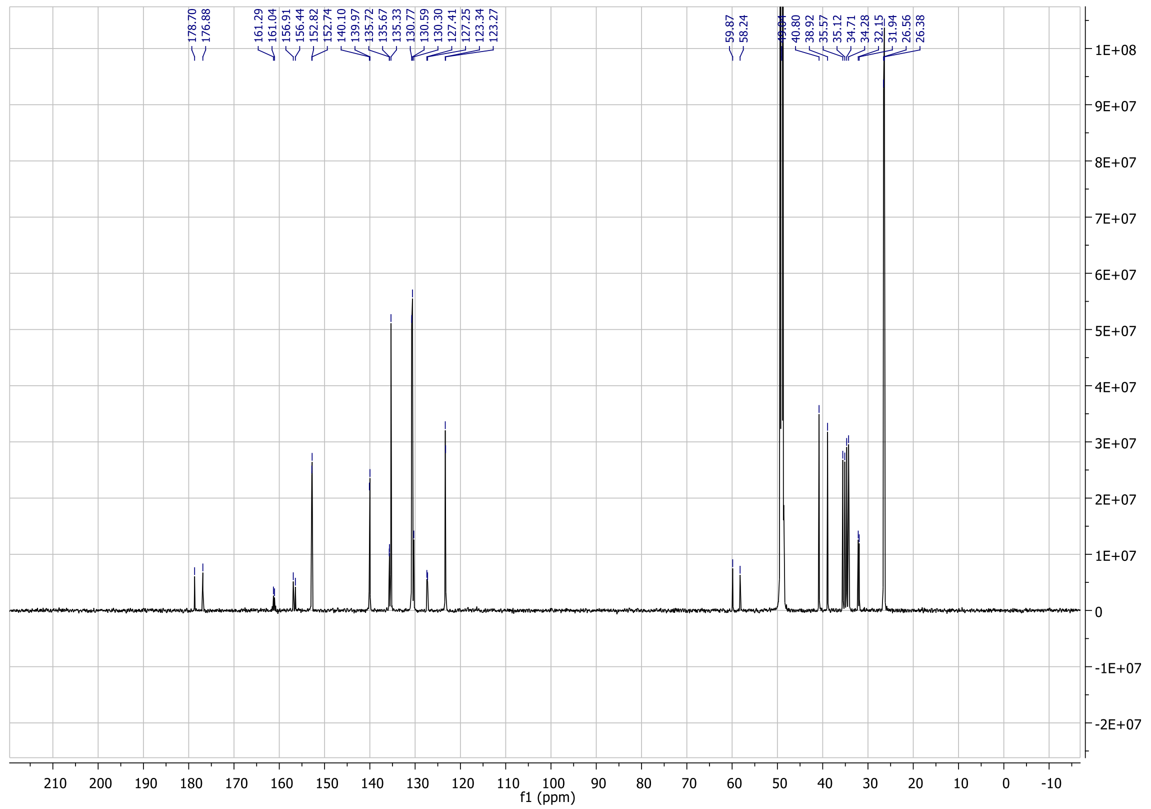

^{1}H NMR (600 MHz, CD_3OD) spectrum of compound **17**

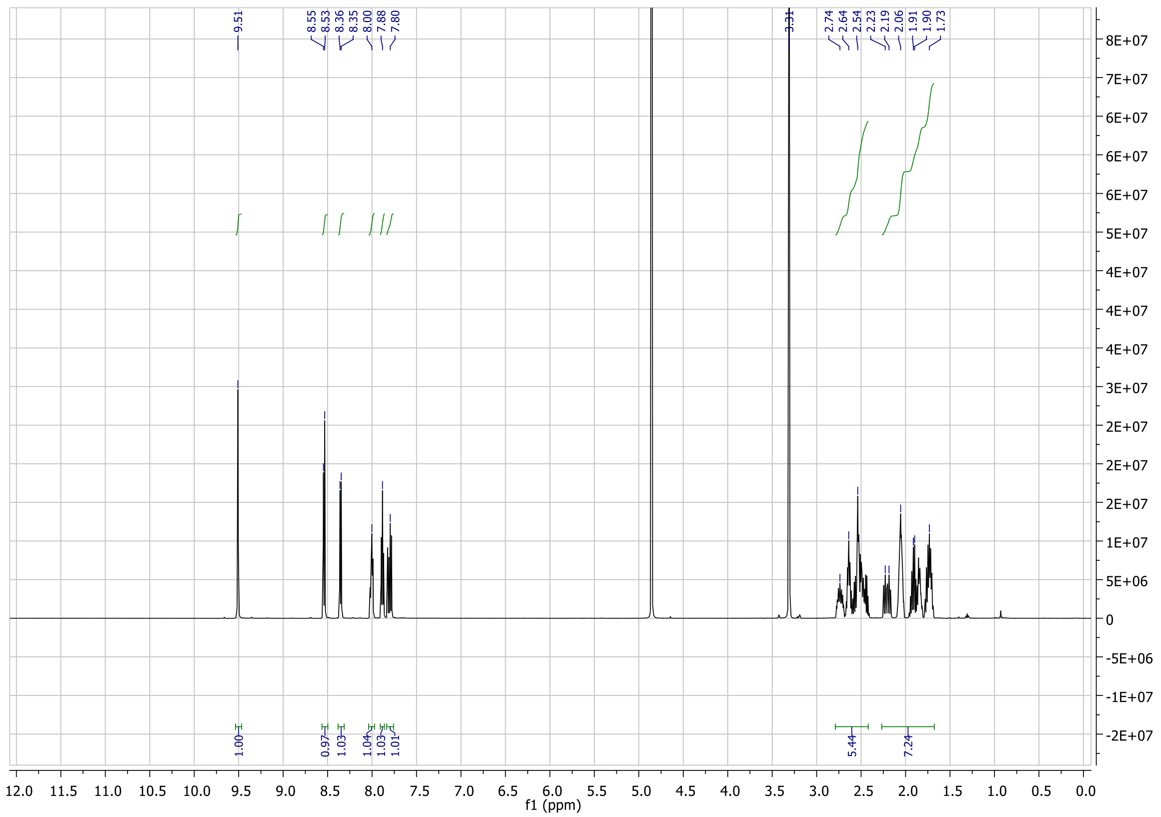

^{13}C NMR (150 MHz, CD_3OD) spectrum of compound **17**

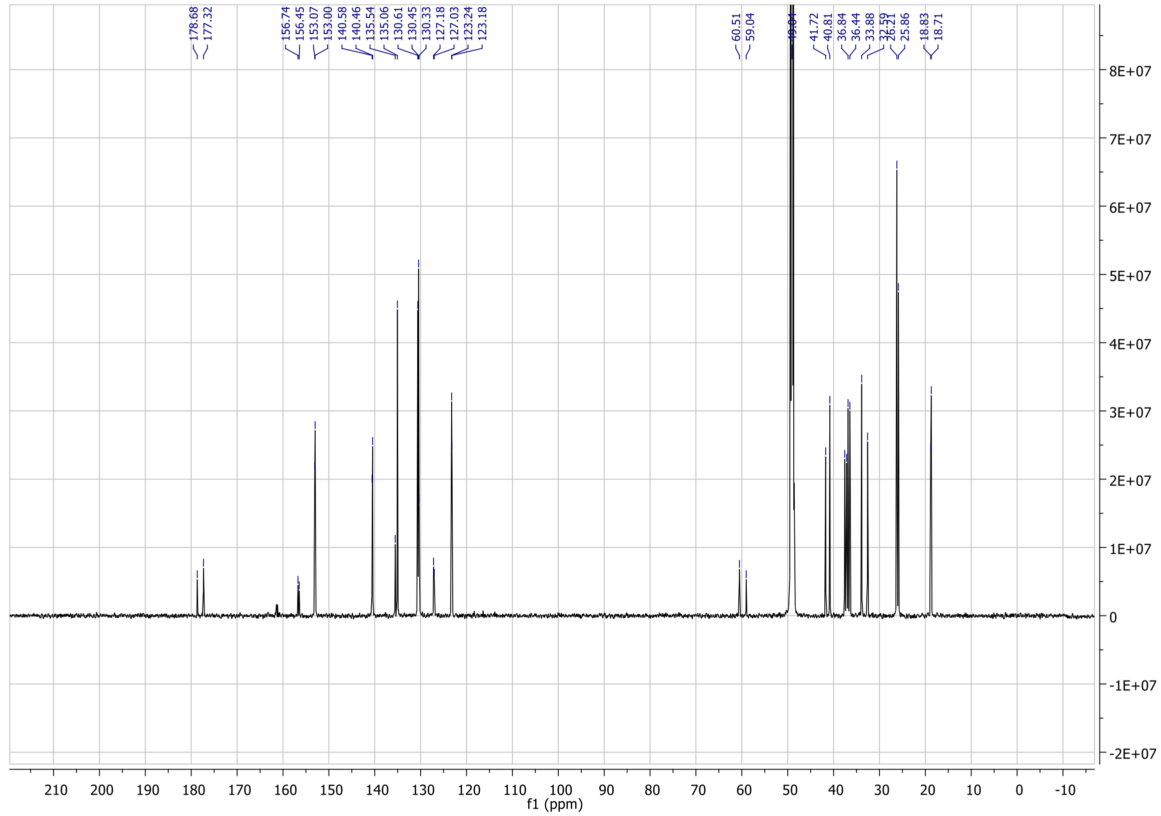

^{1}H NMR (600 MHz, CD_3OD) spectrum of compound **18**

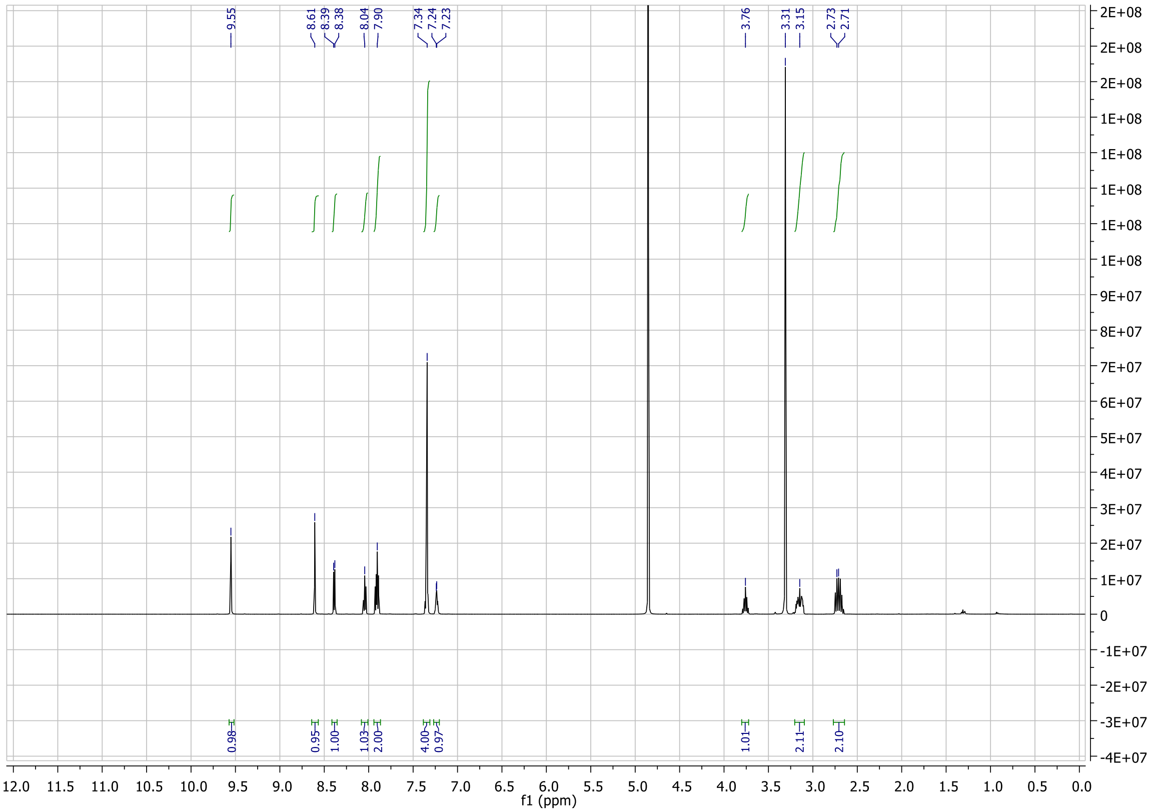

^{13}C NMR (150 MHz, CD_3OD) spectrum of compound **18**

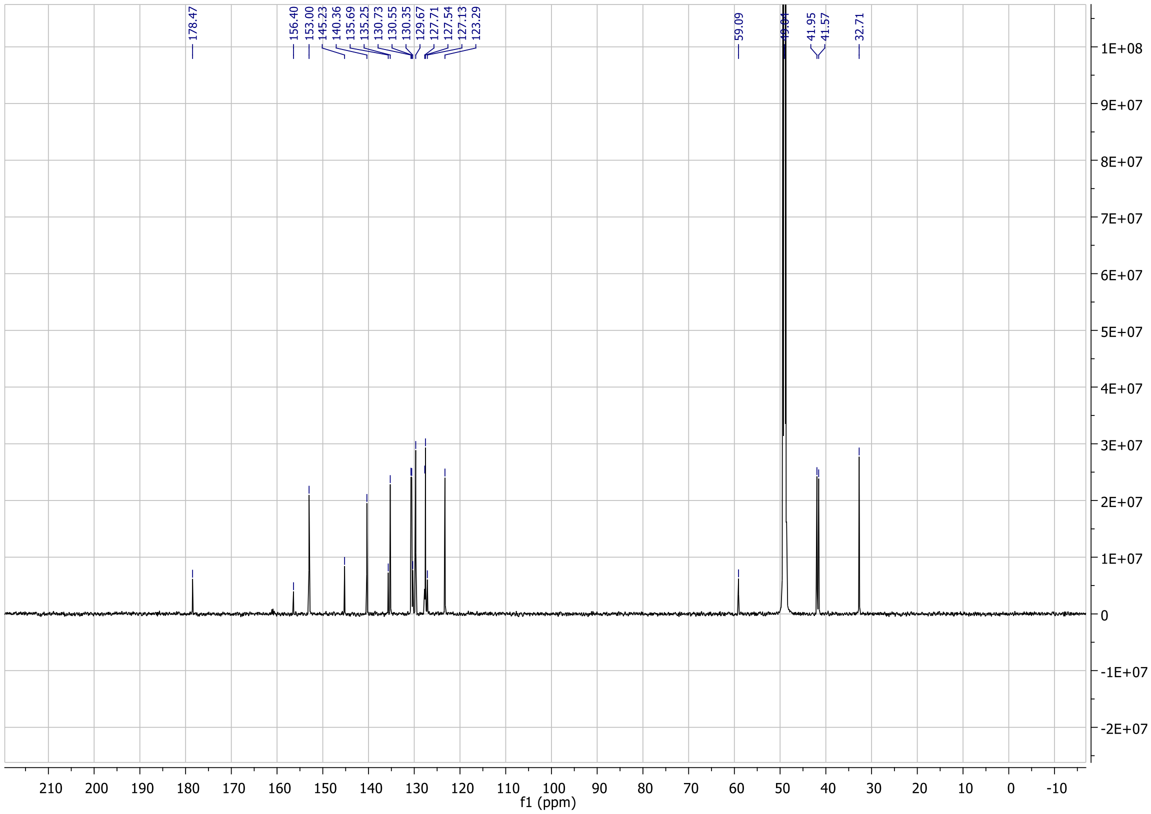

^{1}H NMR (600 MHz, $CDCl_3$) spectrum of compound **19**

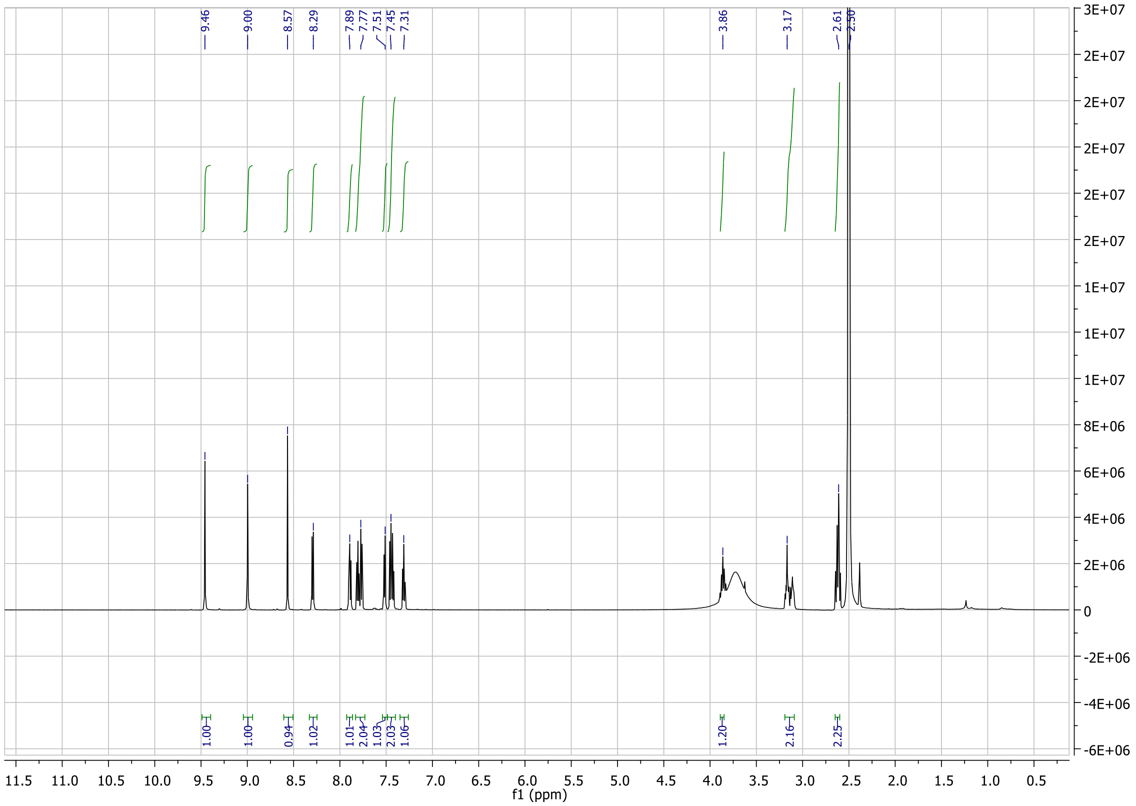

^{13}C NMR (150 MHz, $CDCl_3$) spectrum of compound **19**

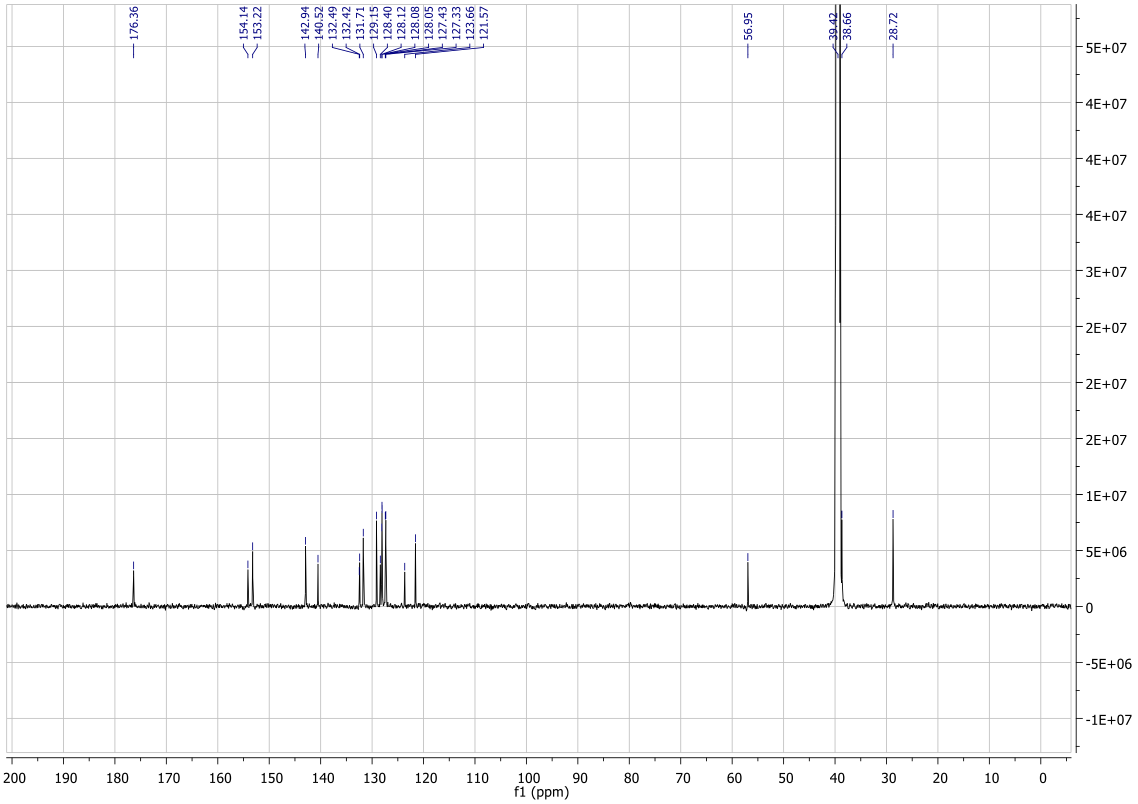

^1H NMR (600 MHz, $CDCl_3$) spectrum of compound **22**

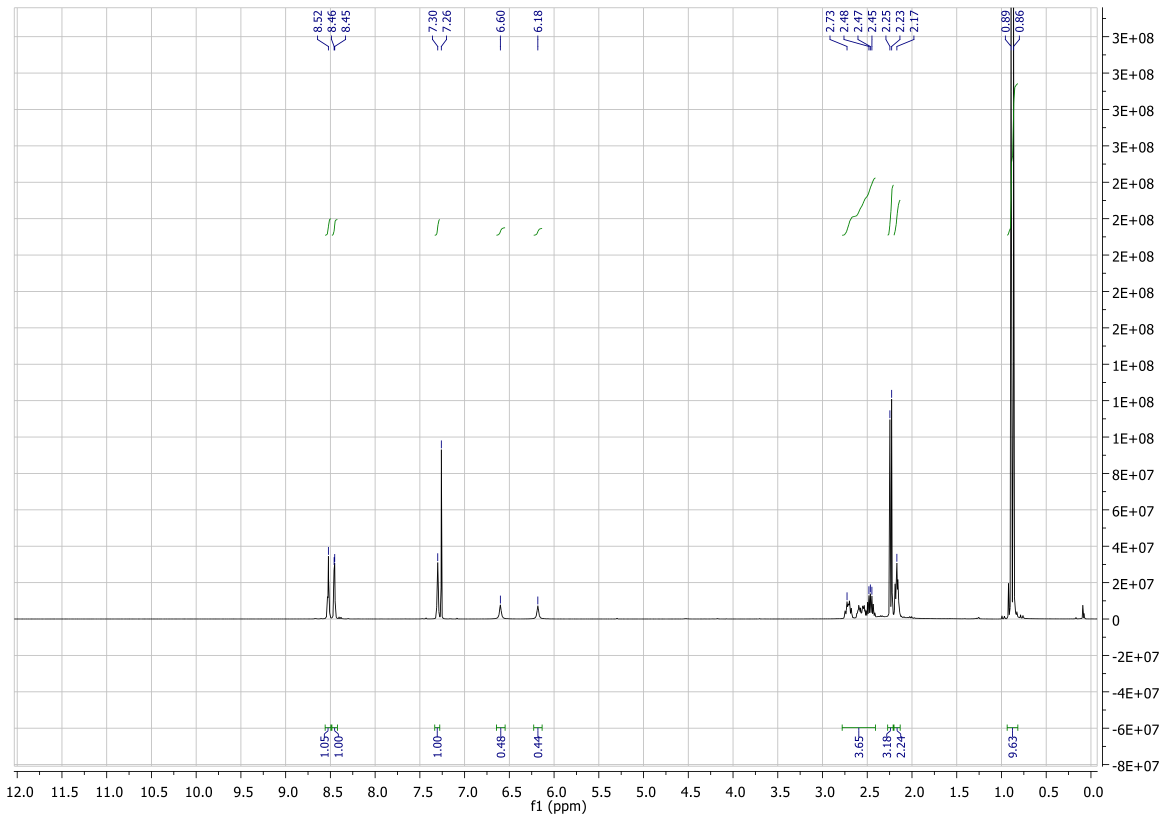

^{13}C NMR (150 MHz, $CDCl_3$) spectrum of compound **22**

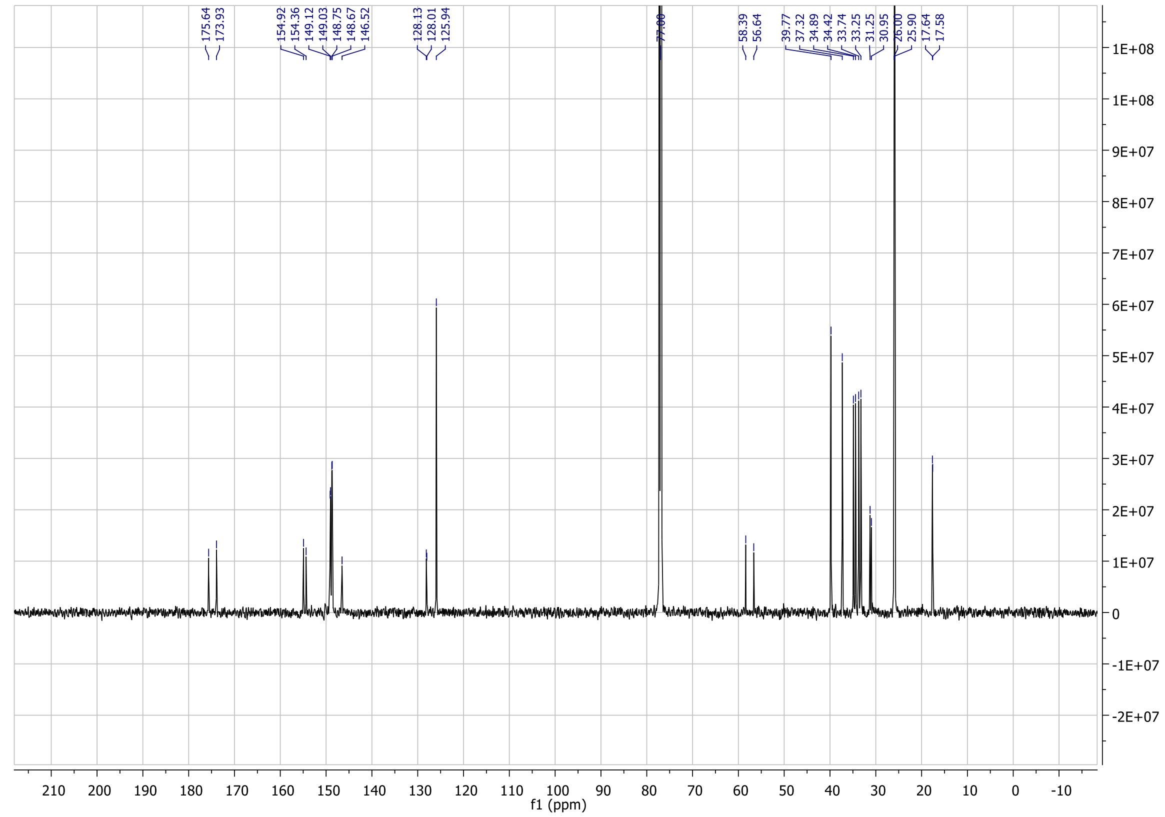

^{1}H NMR (600 MHz, CDCl$_3$) spectrum of compound **23**

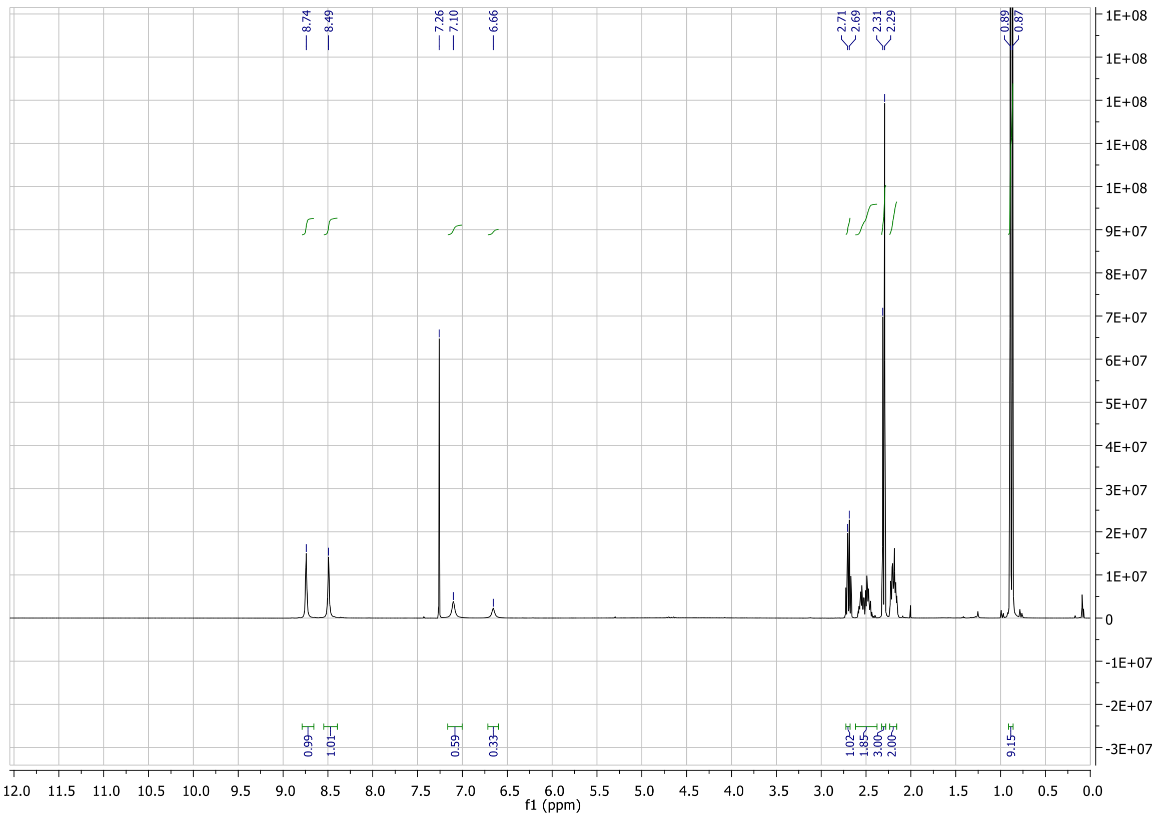

^{13}C NMR (150 MHz, CDCl$_3$) spectrum of compound **23**

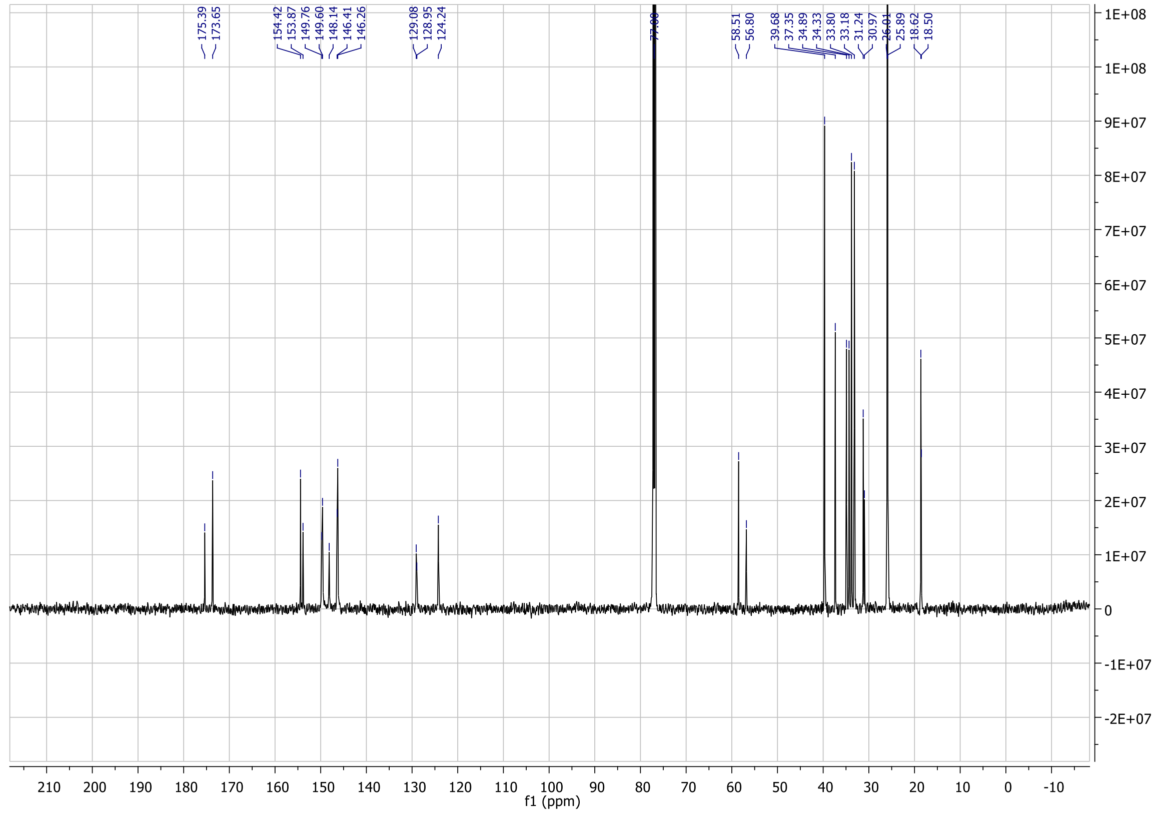

^{1}H NMR (600 MHz, $CDCl_3$) spectrum of compound **24**

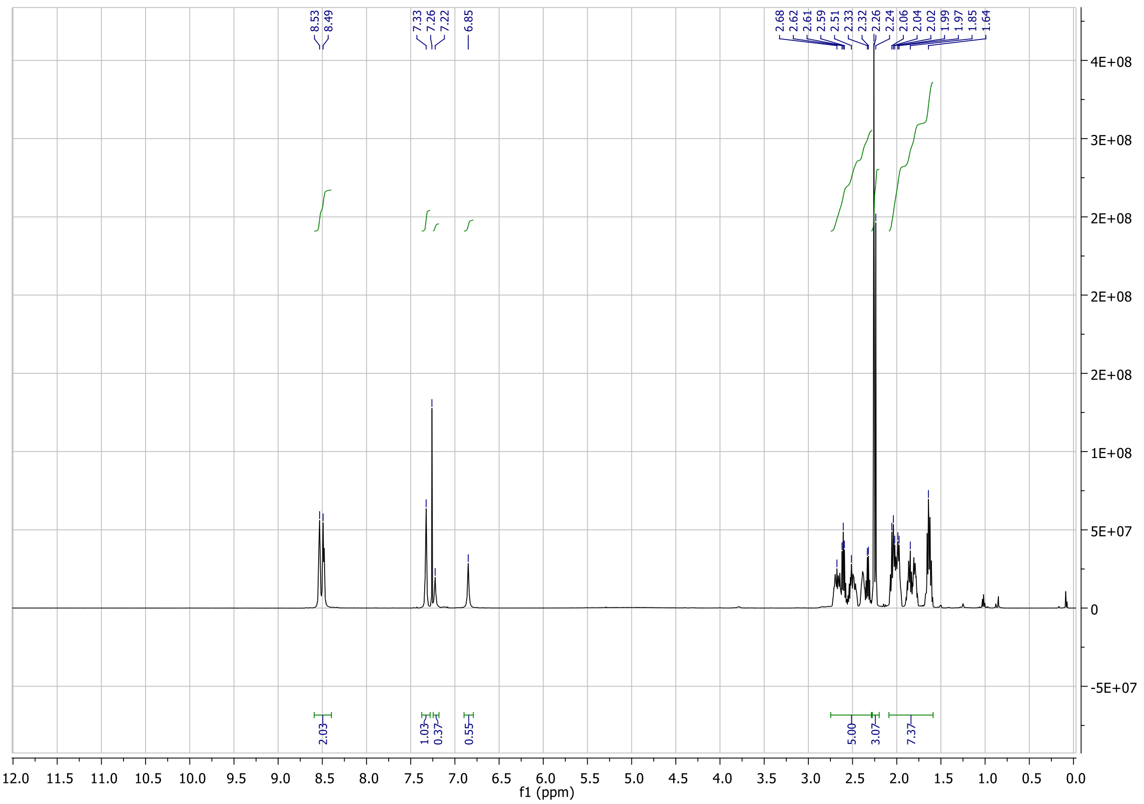

^{13}C NMR (150 MHz, $CDCl_3$) spectrum of compound **24**

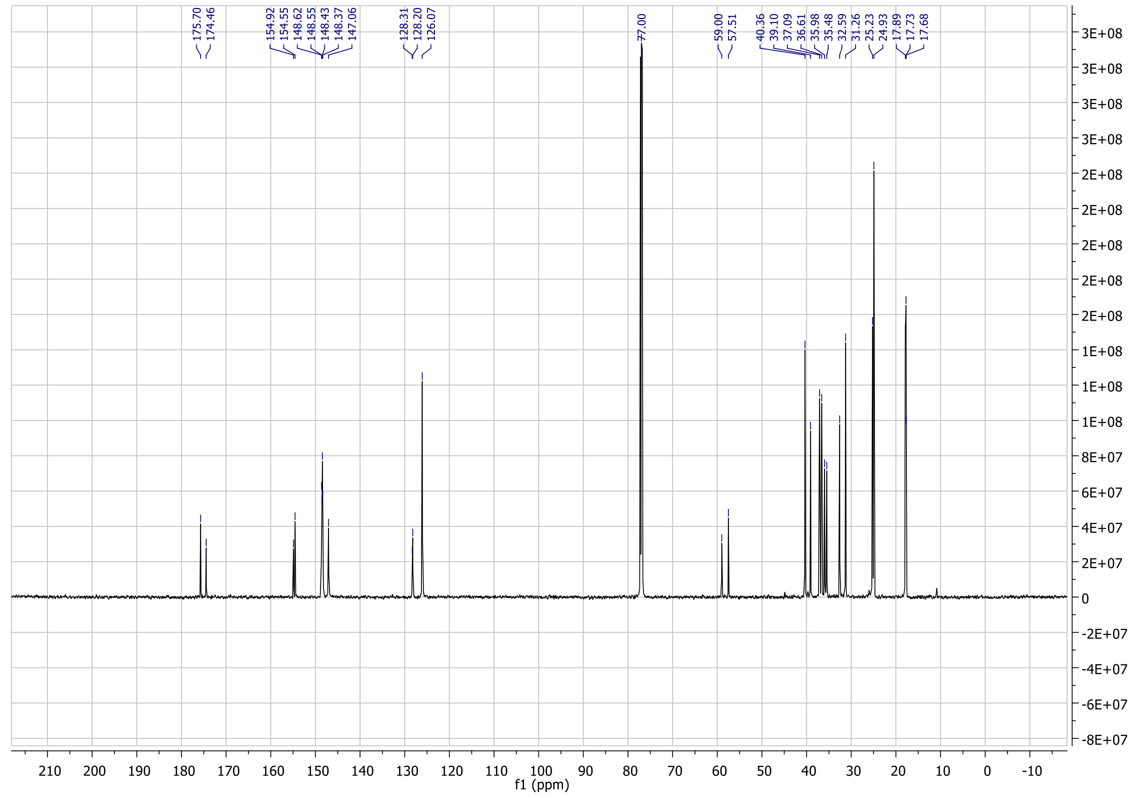

^{1}H NMR (600 MHz, $CDCl_3$) spectrum of compound **25**

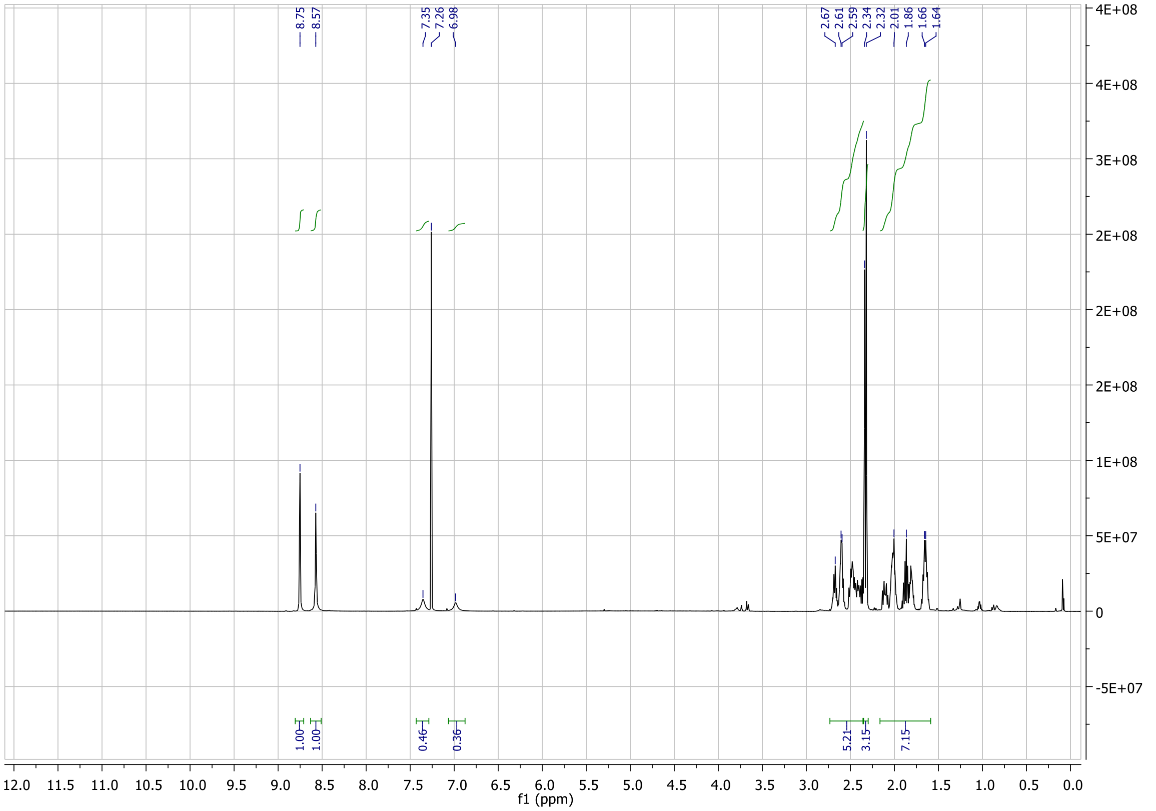

^{13}C NMR (150 MHz, $CDCl_3$) spectrum of compound **25**

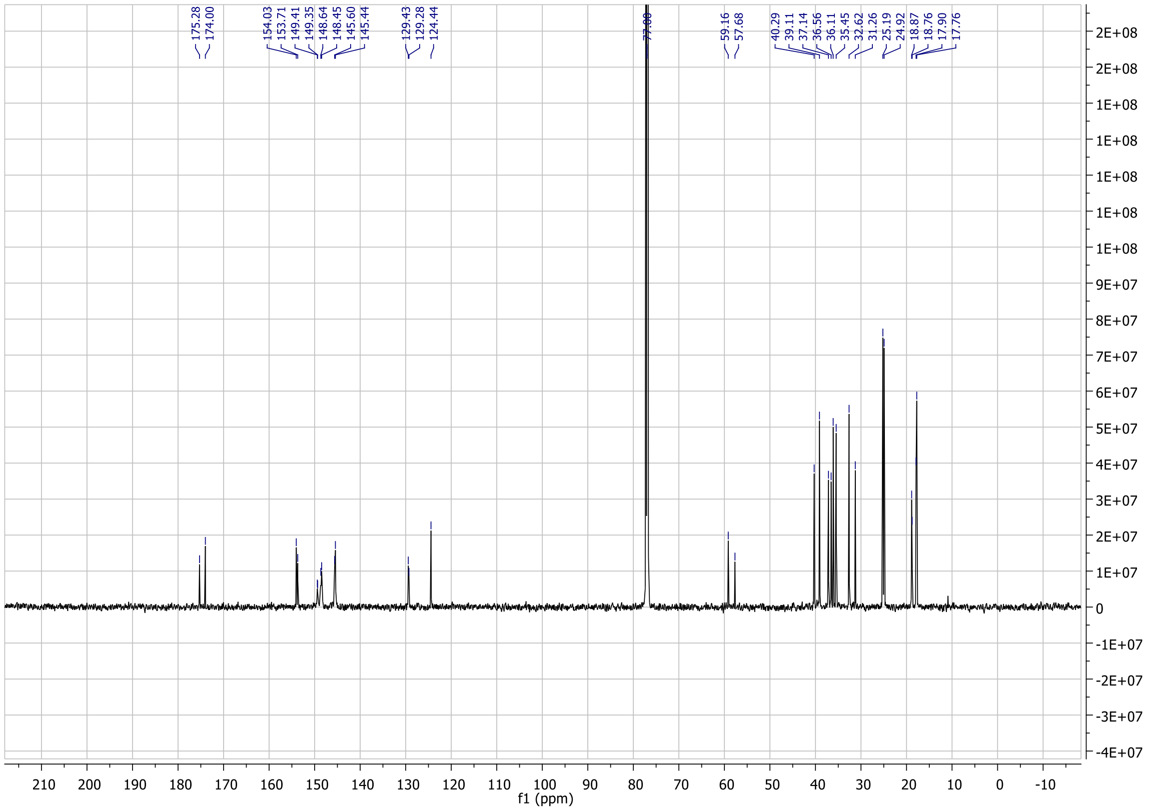

^{1}H NMR (600 MHz, DMSO-d$_6$) spectrum of compound **17a**

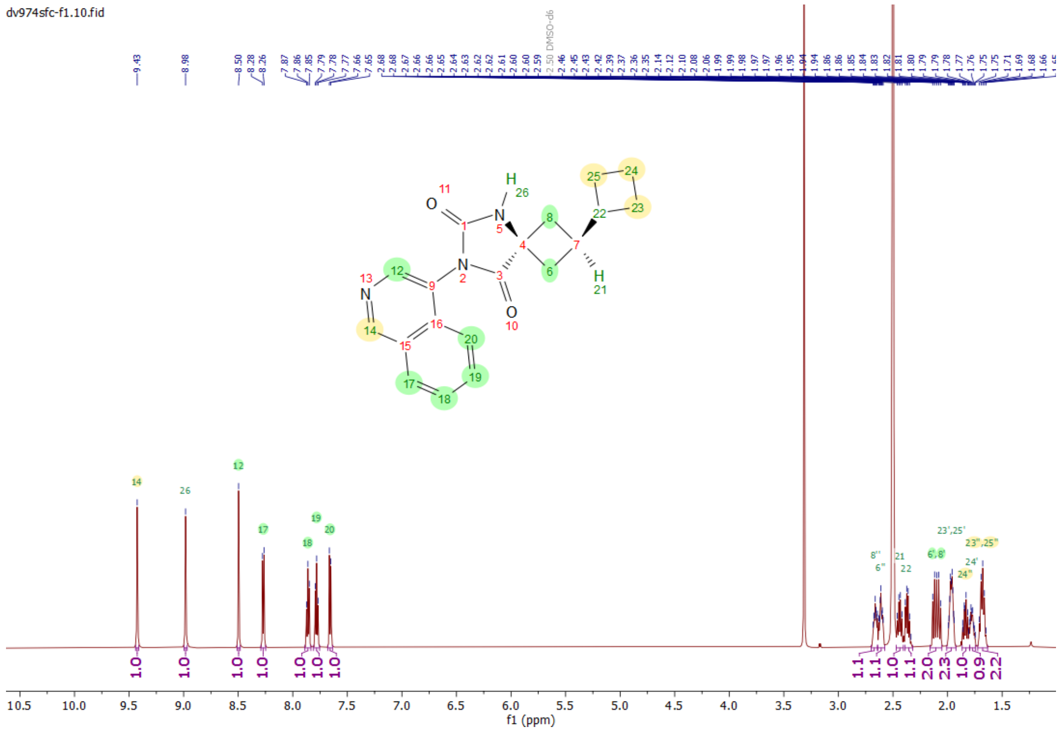

HSQC spectrum of compound **17a**

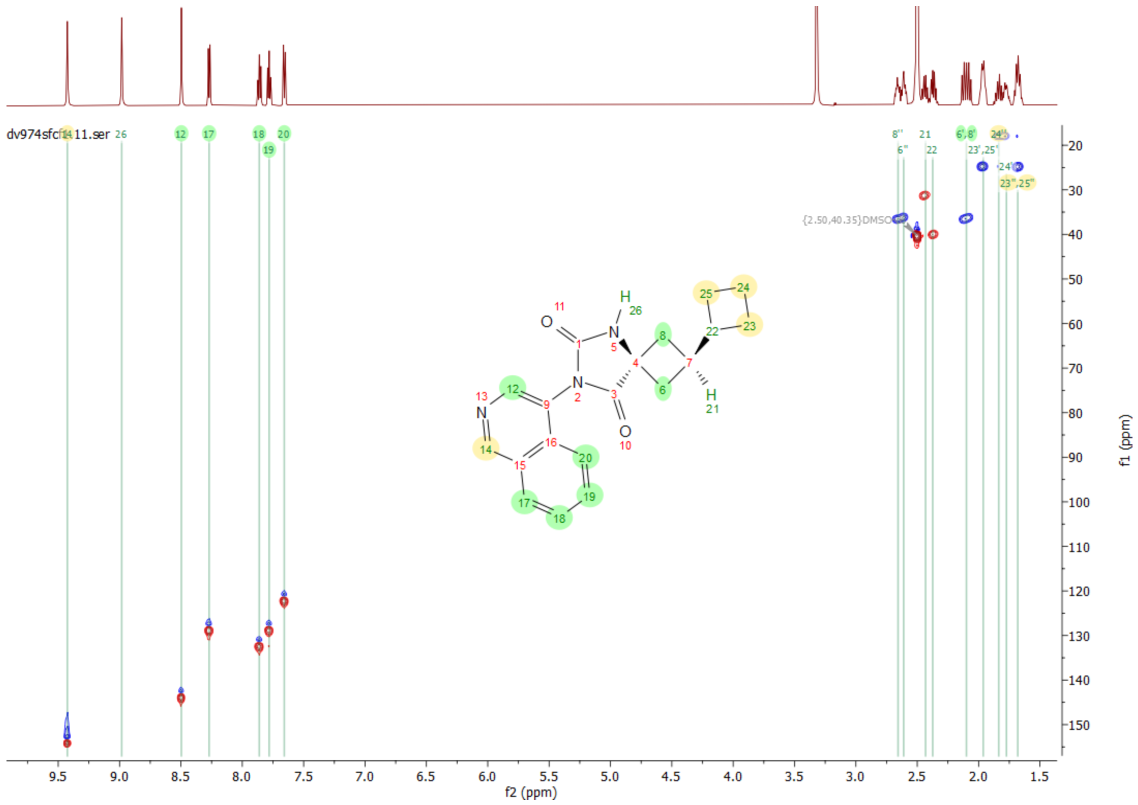

HMBC spectrum of compound **17a**

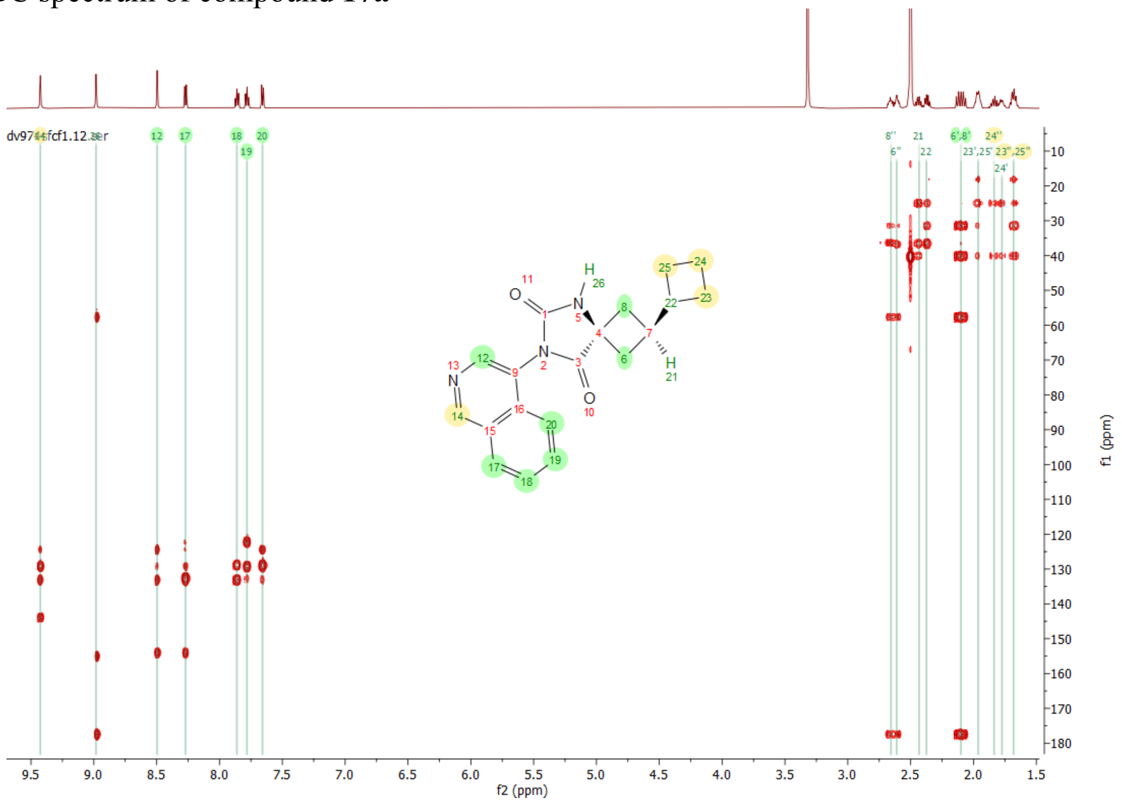

NOESY spectrum of compound **17a**

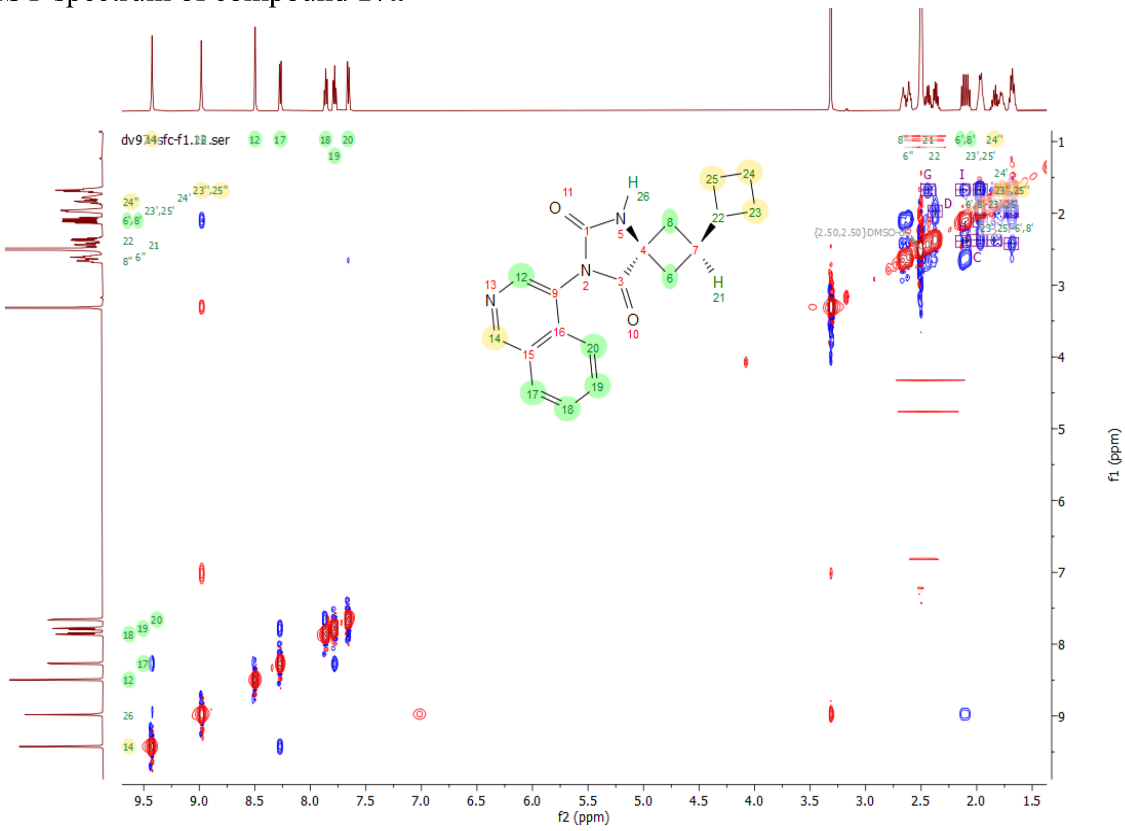

Expanded NOESY spectrum of compound **17a**

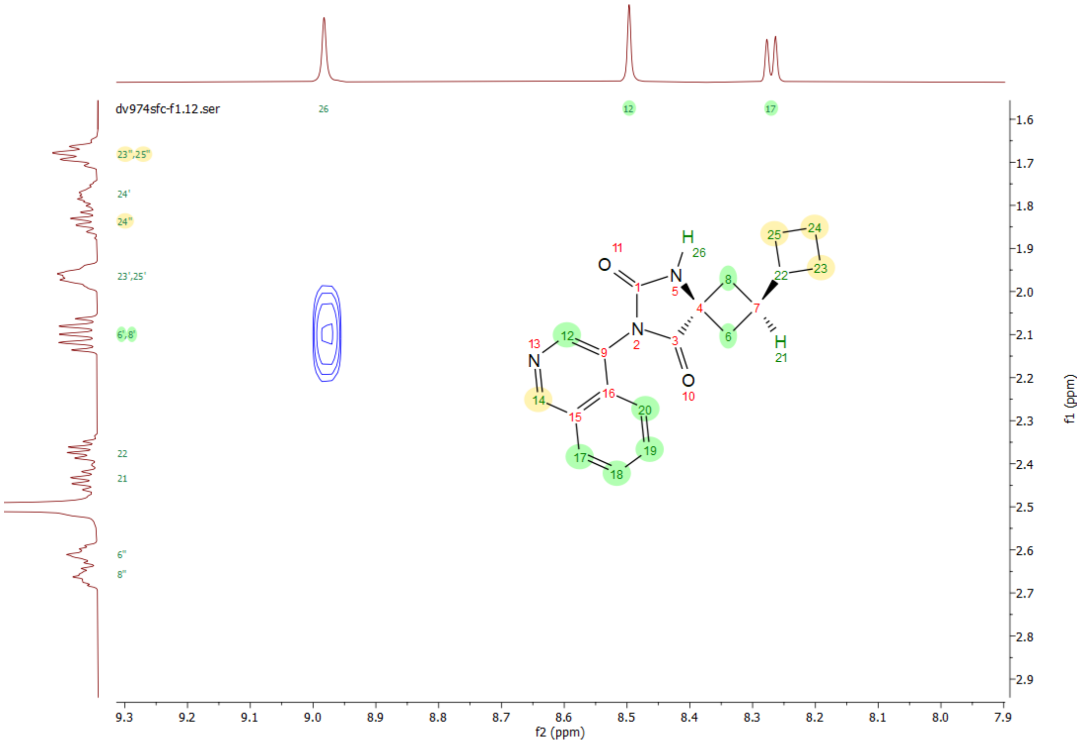

^{1}H NMR (600 MHz, DMSO-d$_6$) spectrum of compound **17b**

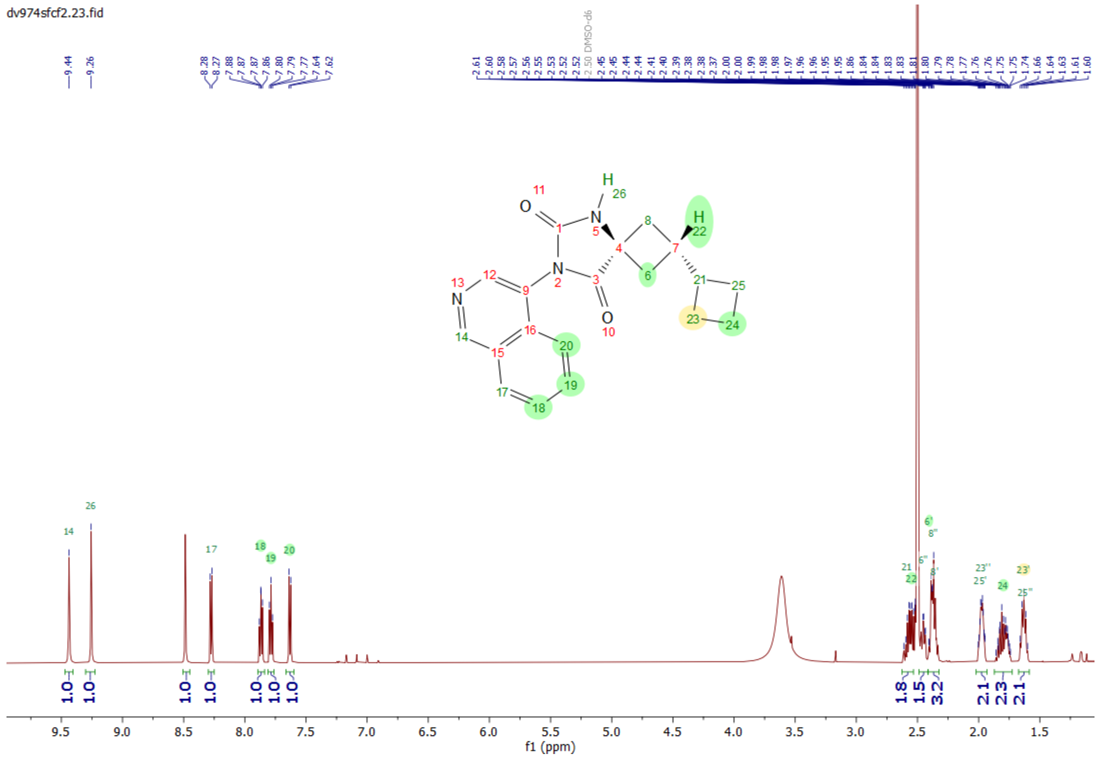

HSQC spectrum of compound **17b**

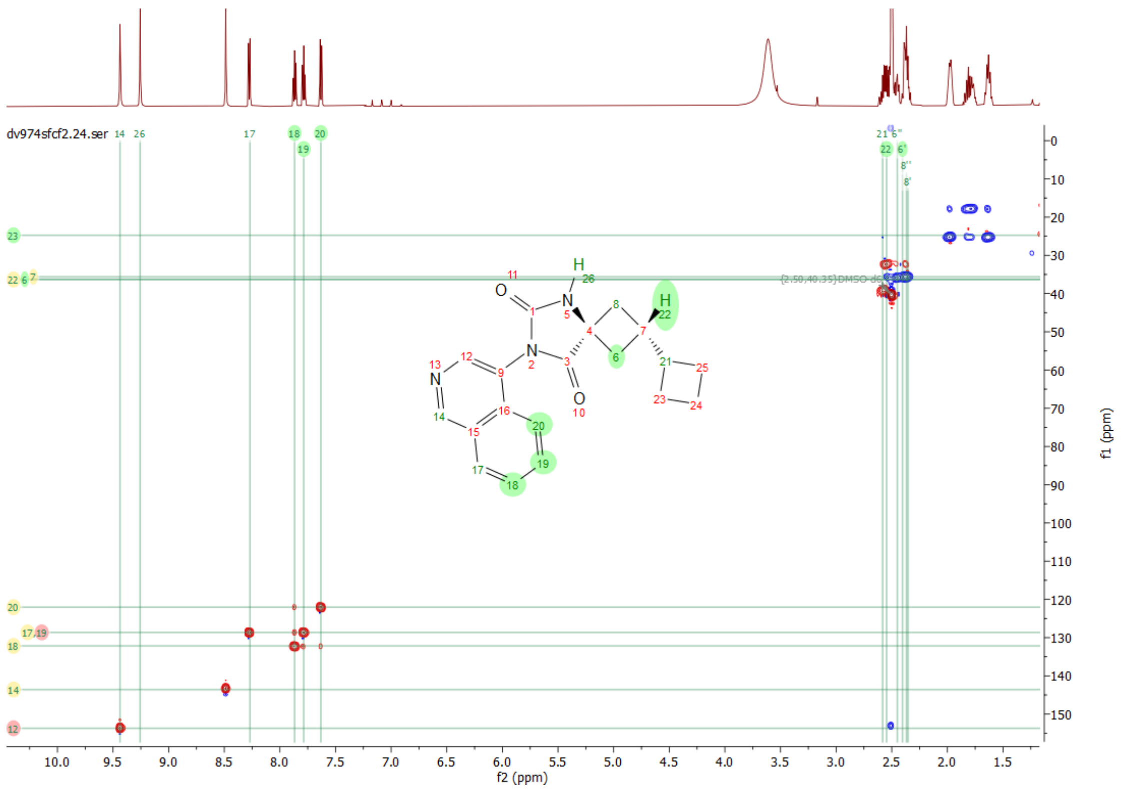

HMBC spectrum of compound **17b**

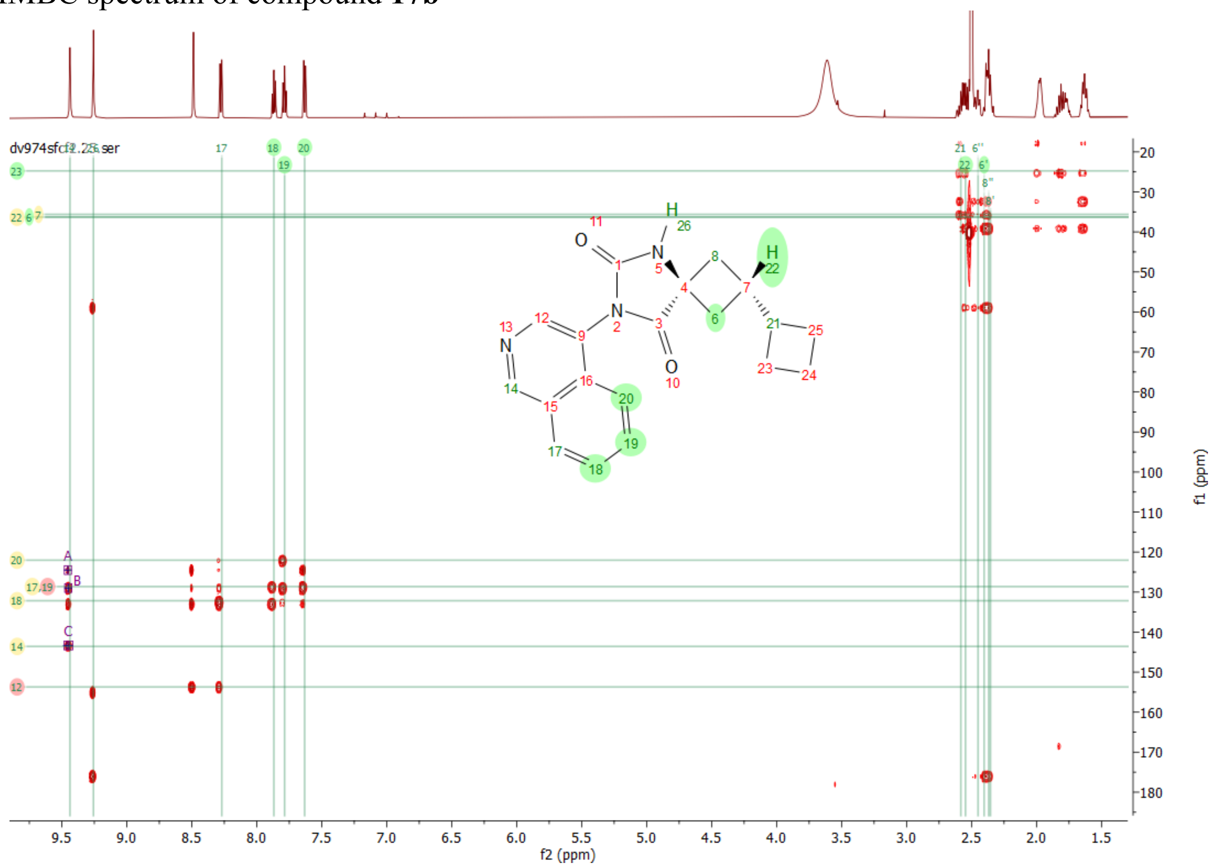

NOESY spectrum of compound **17b**

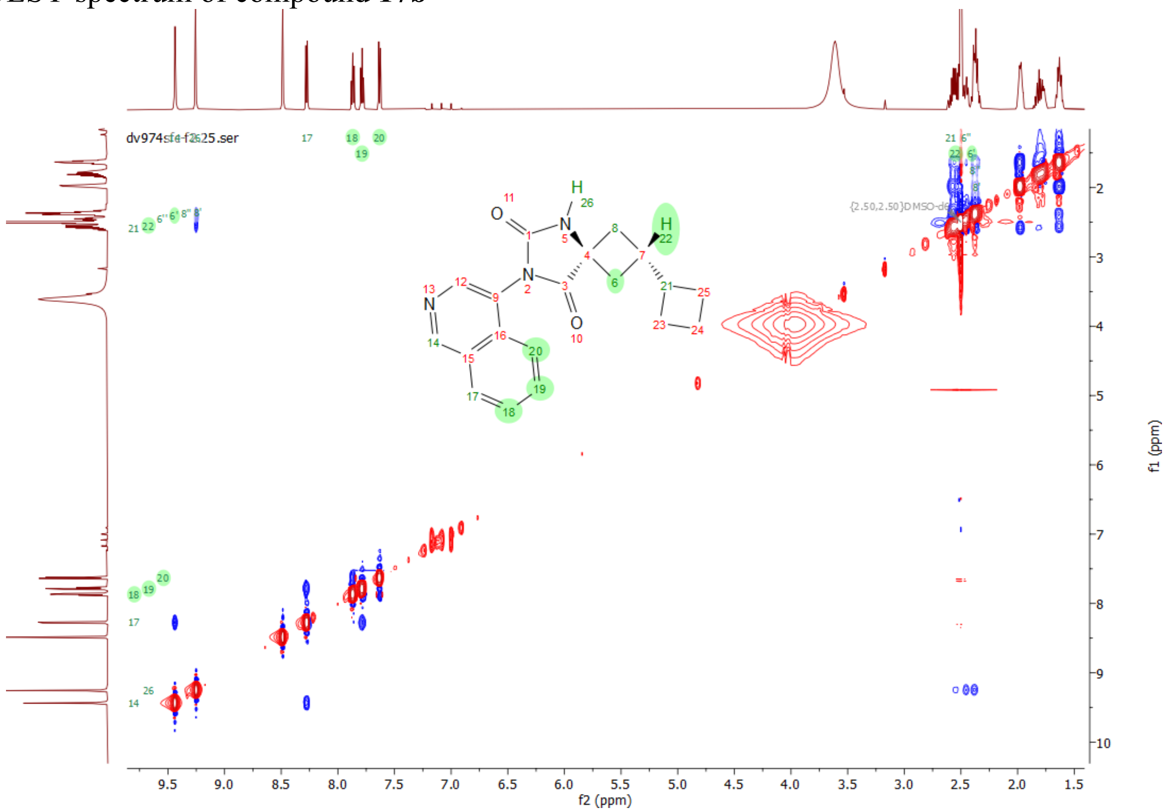

Expanded NOESY spectrum of compound **17b**

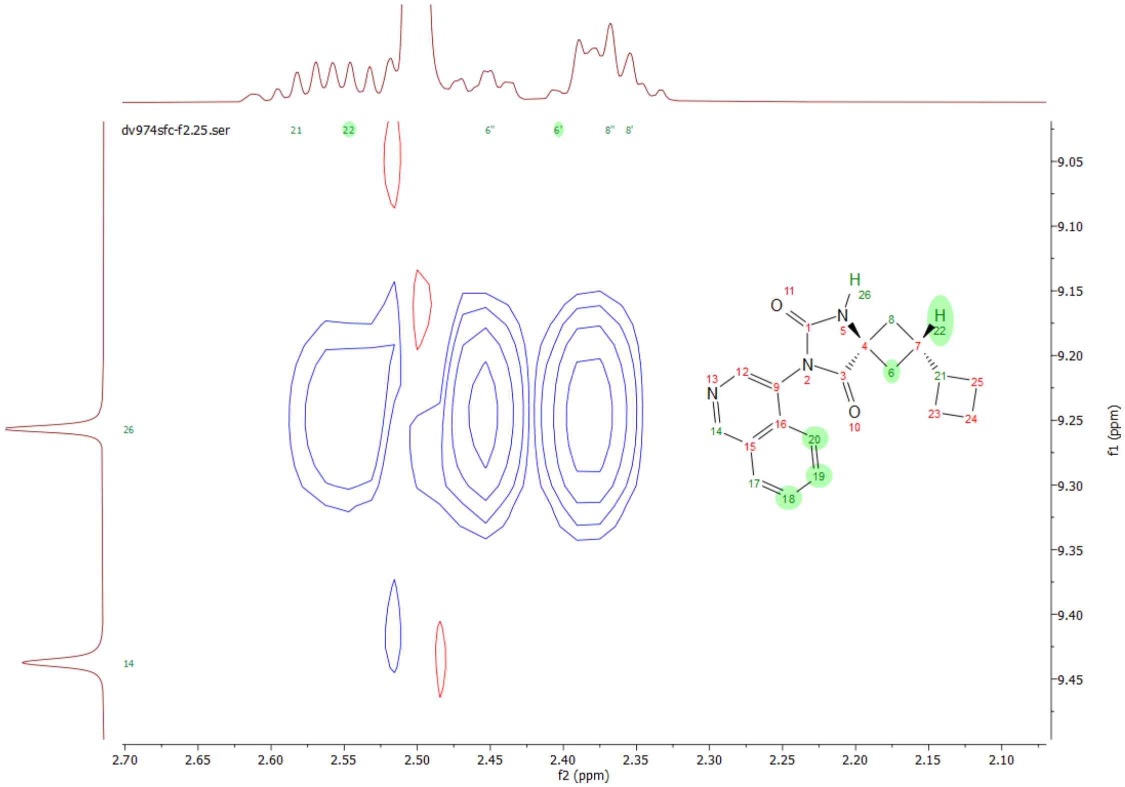

^{1}H NMR (600 MHz, DMSO-d6) spectrum of compound **16a**

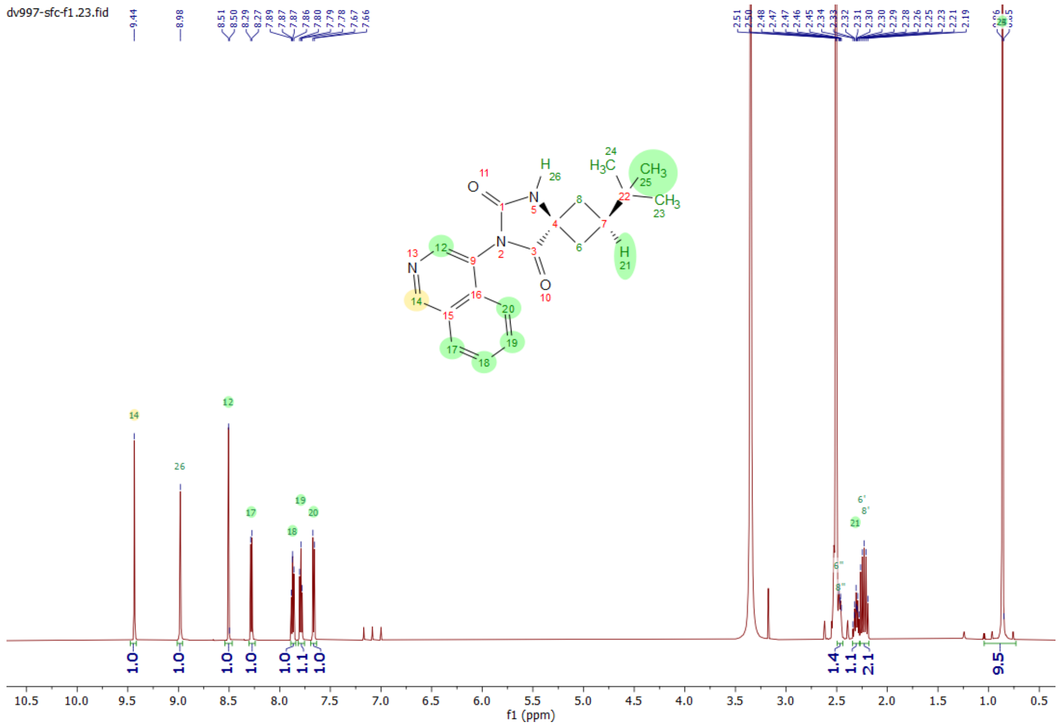

HSQC spectrum of compound **16a**

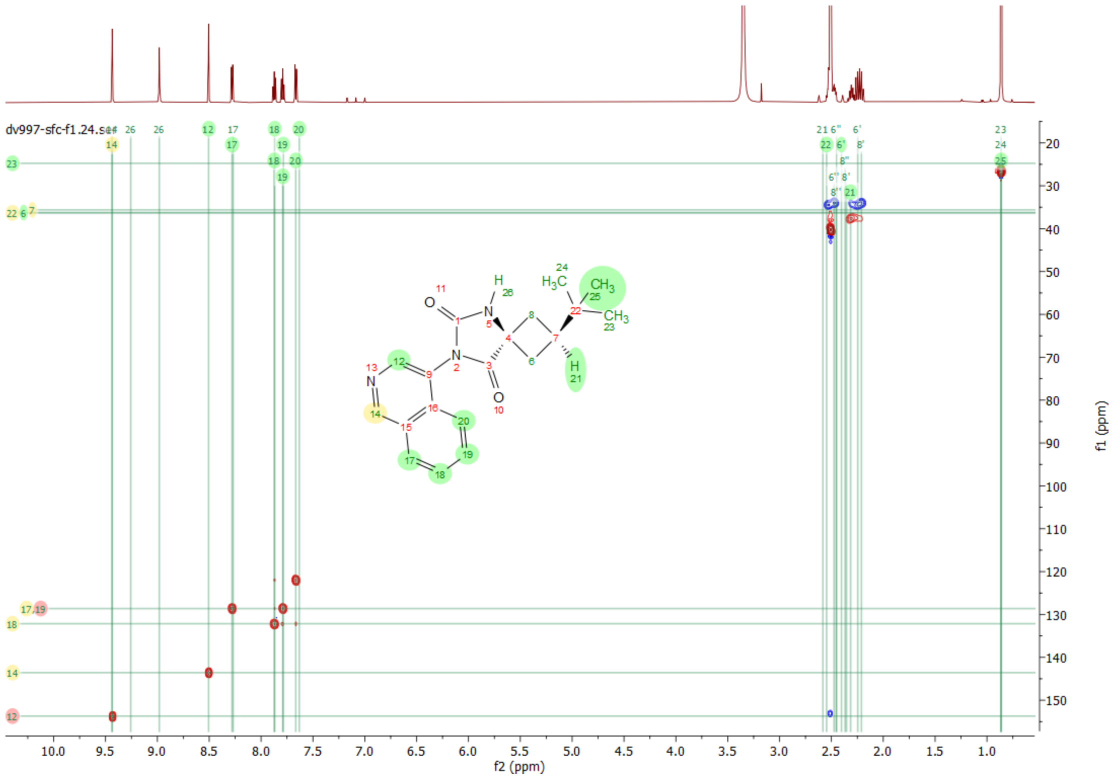

NOESY spectrum of compound **16a**

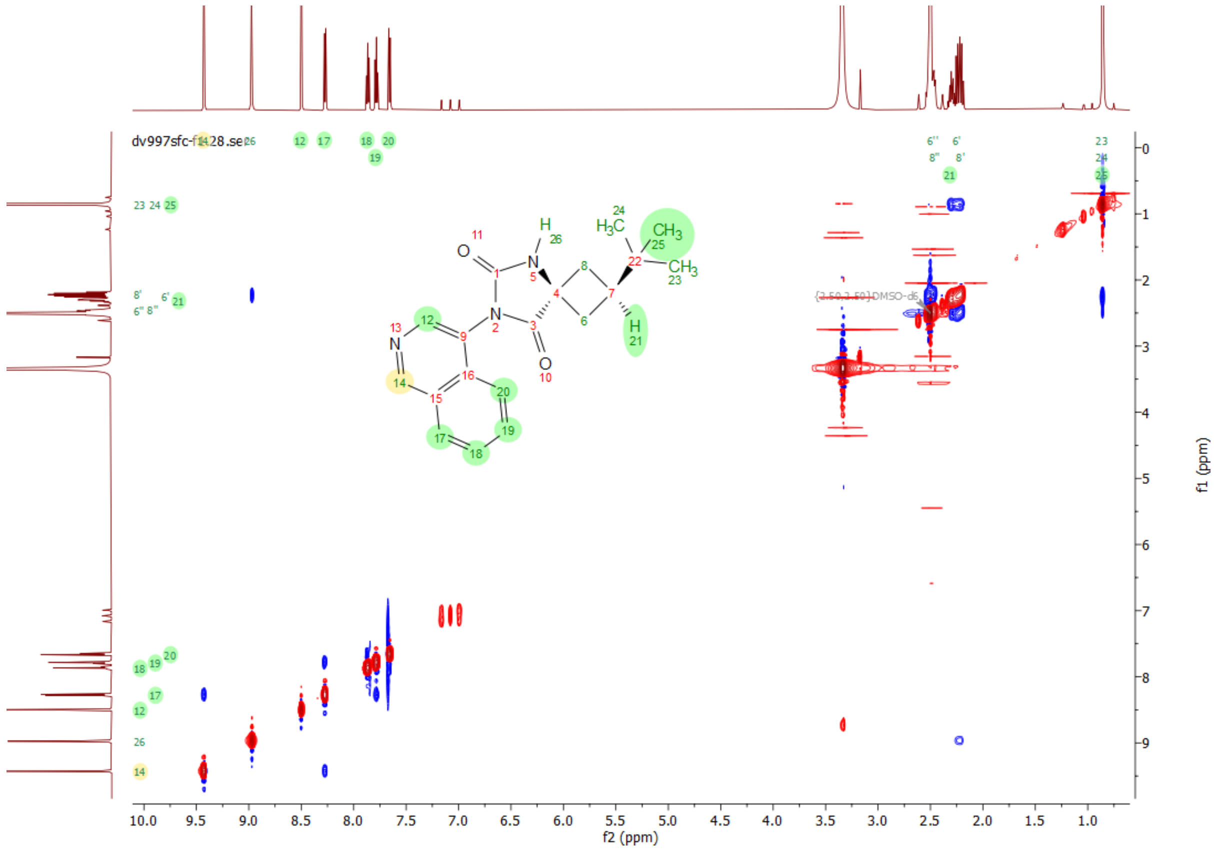

Expanded NOESY spectrum of compound **16a**

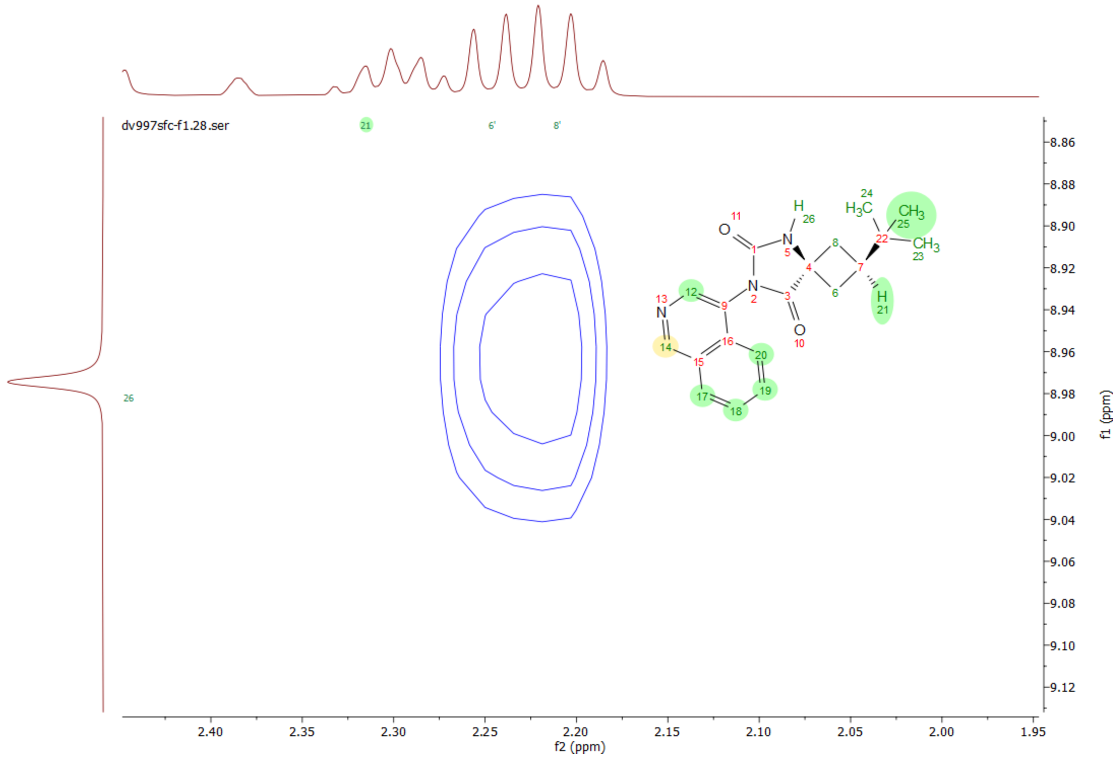

^{1}H NMR (600 MHz, DMSO-d$_6$) spectrum of compound **16b**

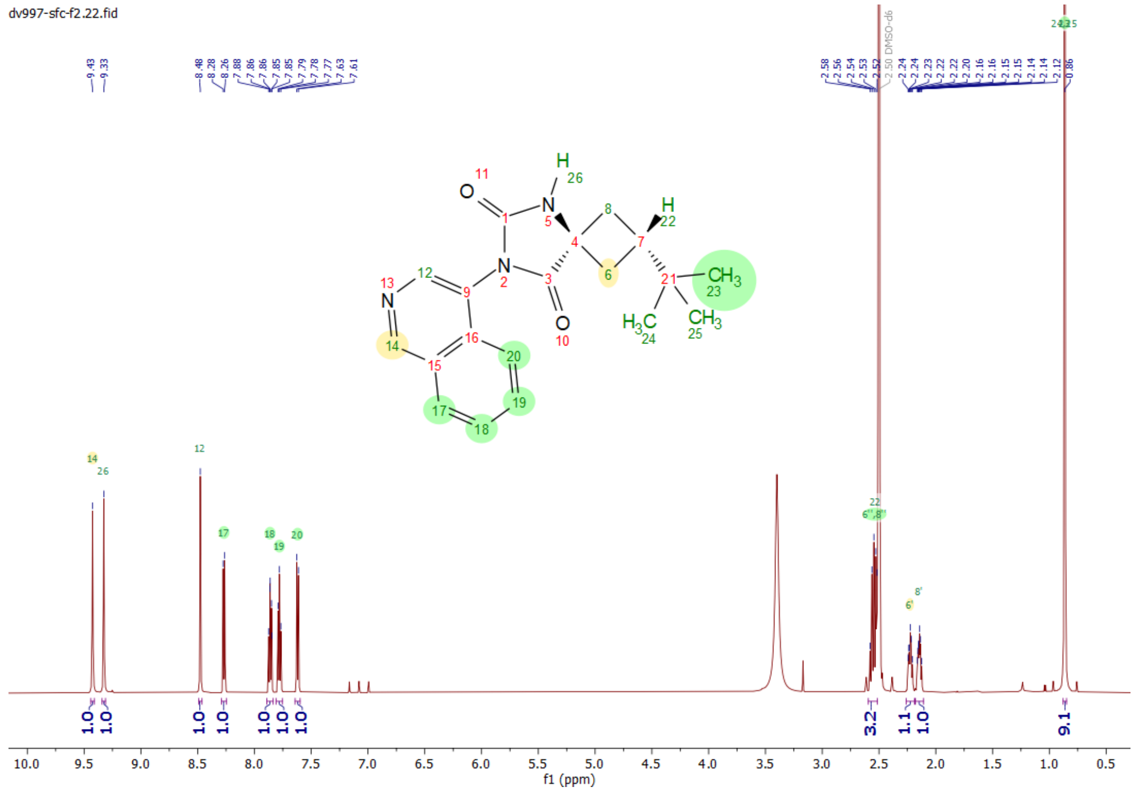

HSQC spectrum of compound **16b**

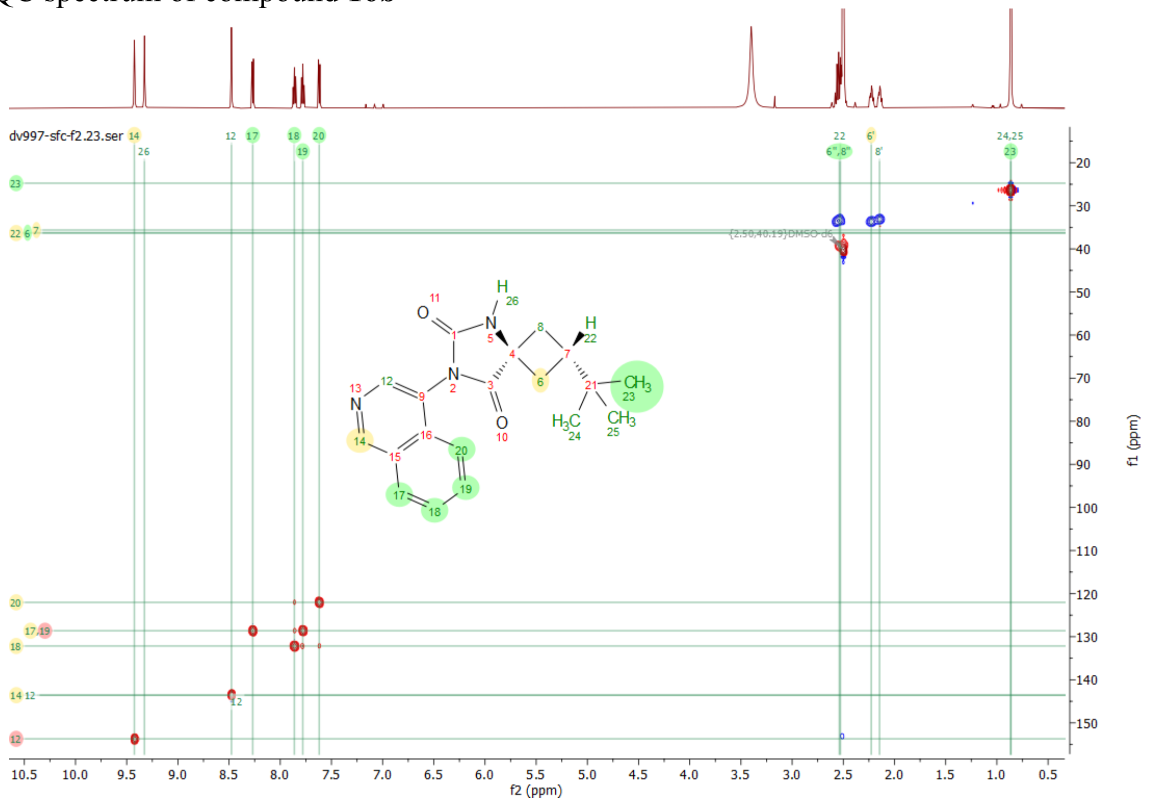

NOESY spectrum of compound **16b**

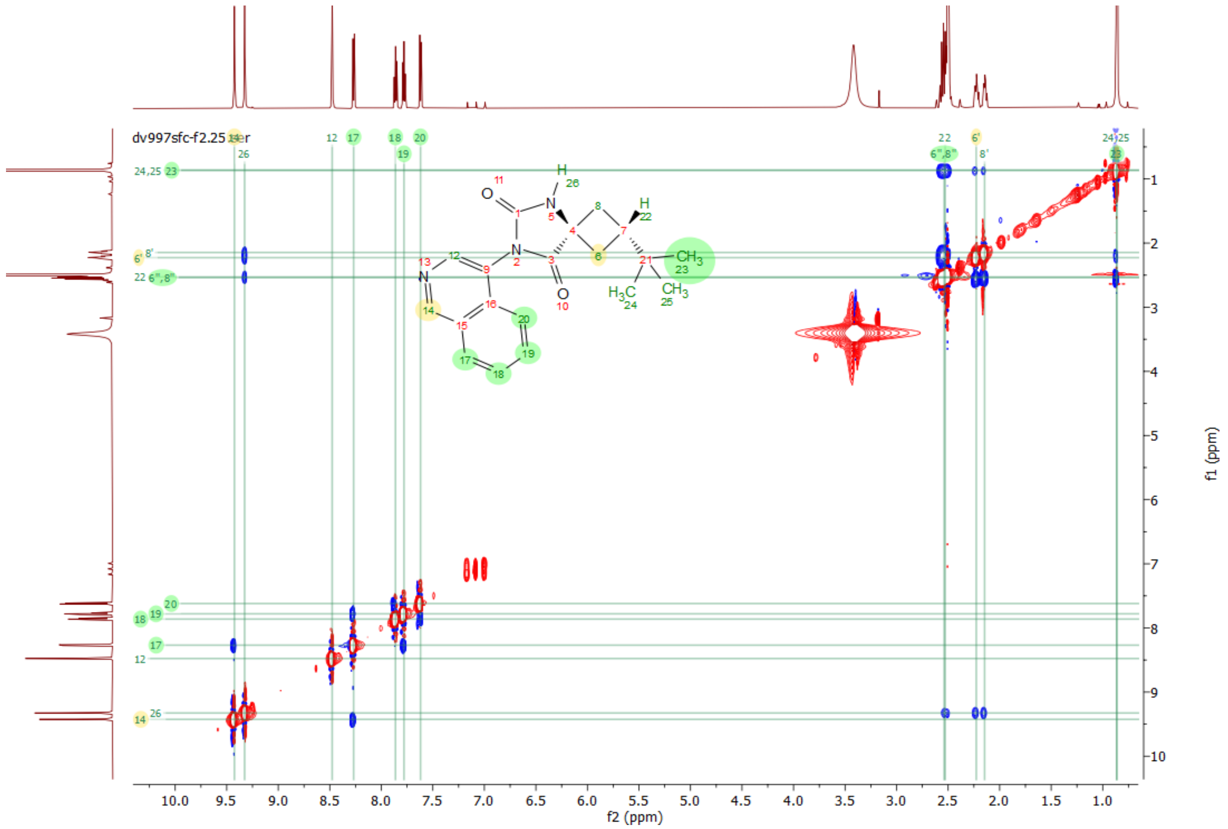

Expanded NOESY spectrum of compound **16b**

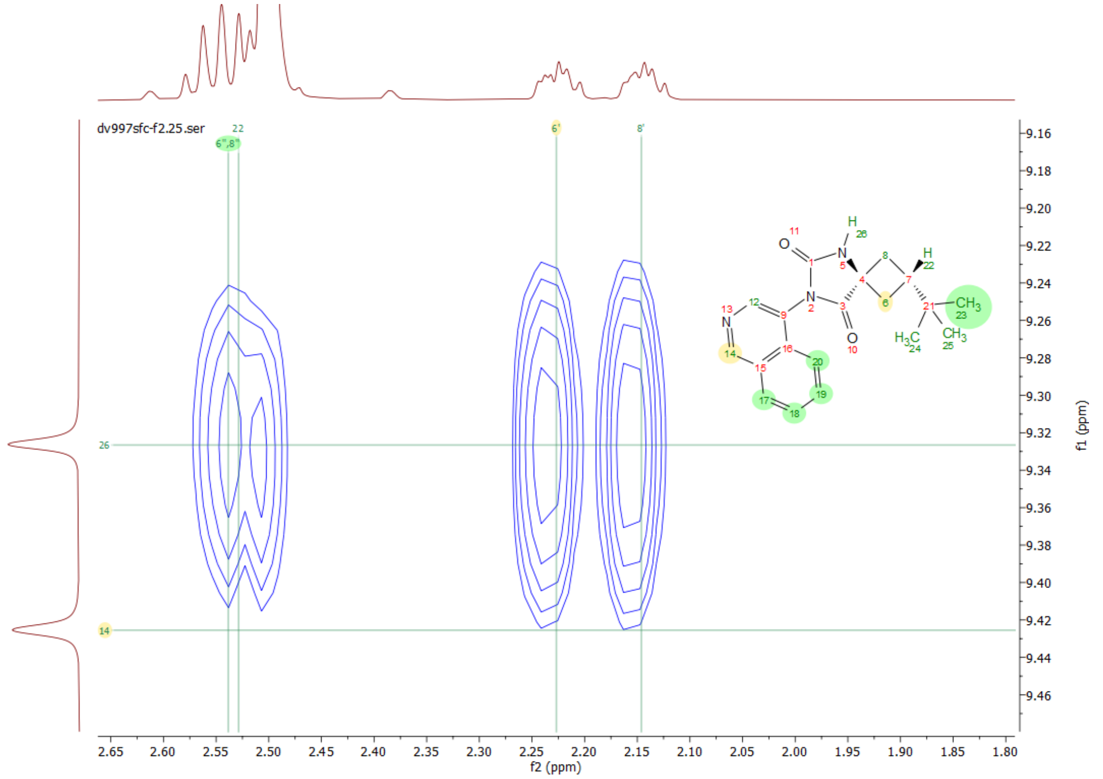

^{1}H NMR (400 MHz, $CDCl_3$) spectrum of **ML188**

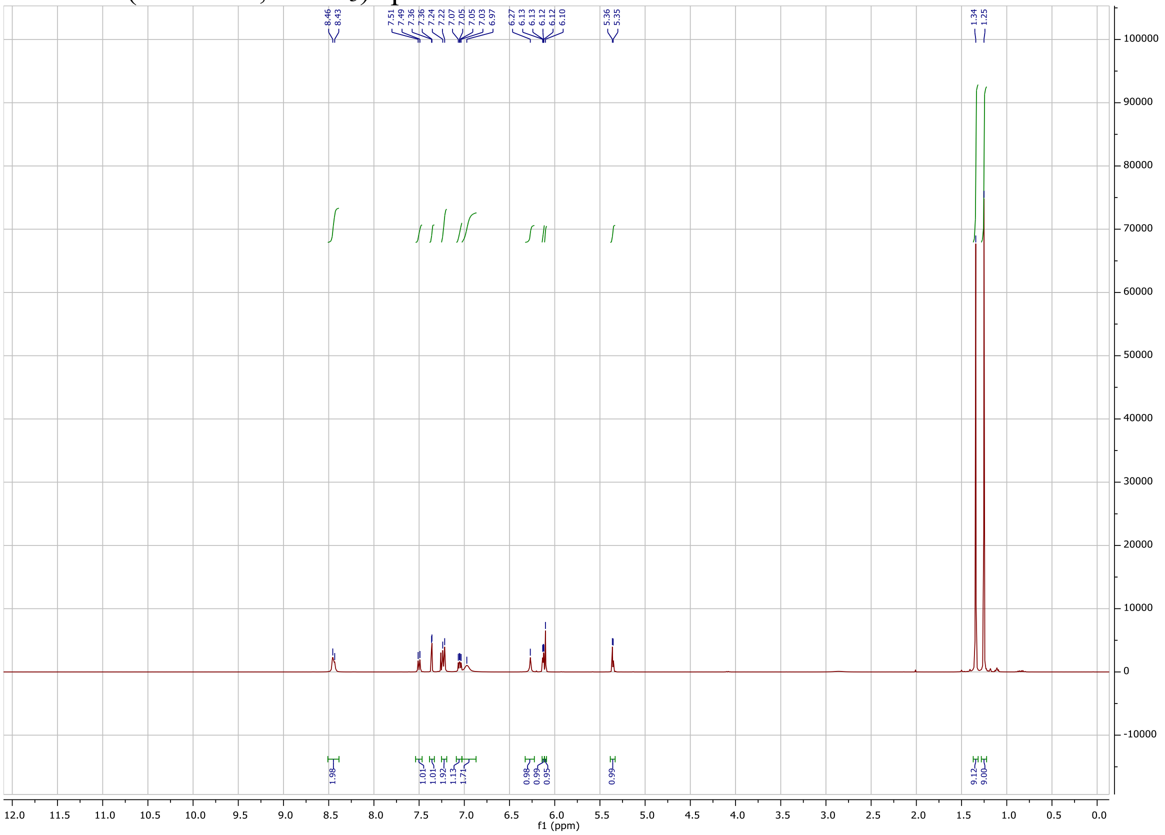

^{13}C NMR (101 MHz, $CDCl_3$) spectrum of **ML188**

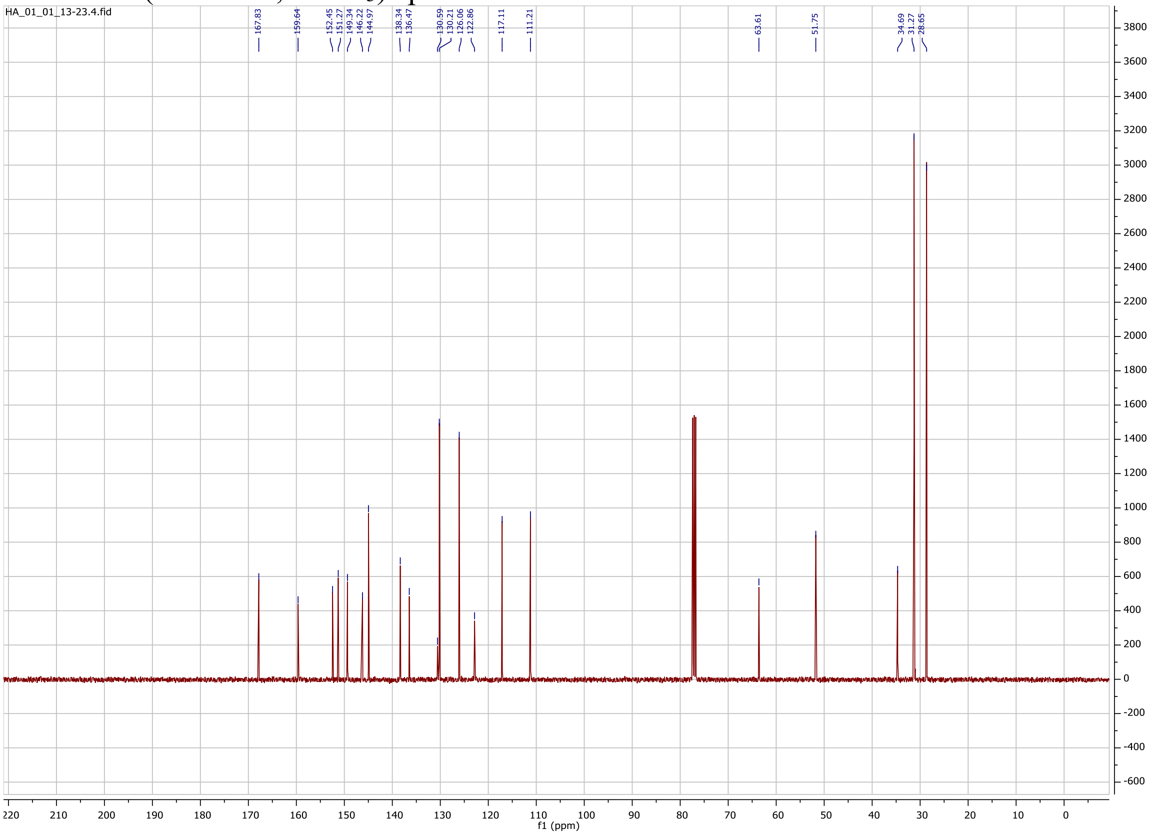

^{1}H NMR (400 MHz, MeOD) spectrum of **X77**

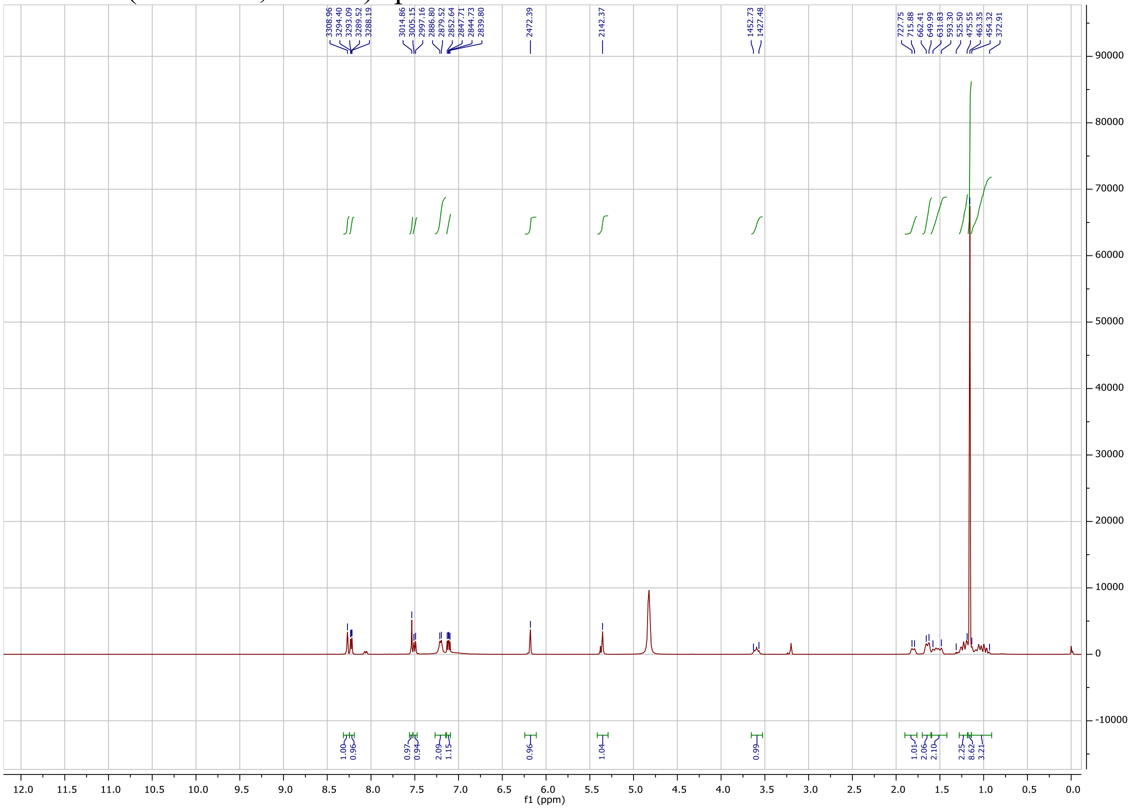

^{13}C NMR (101 MHz, MeOD) spectrum of **X77**

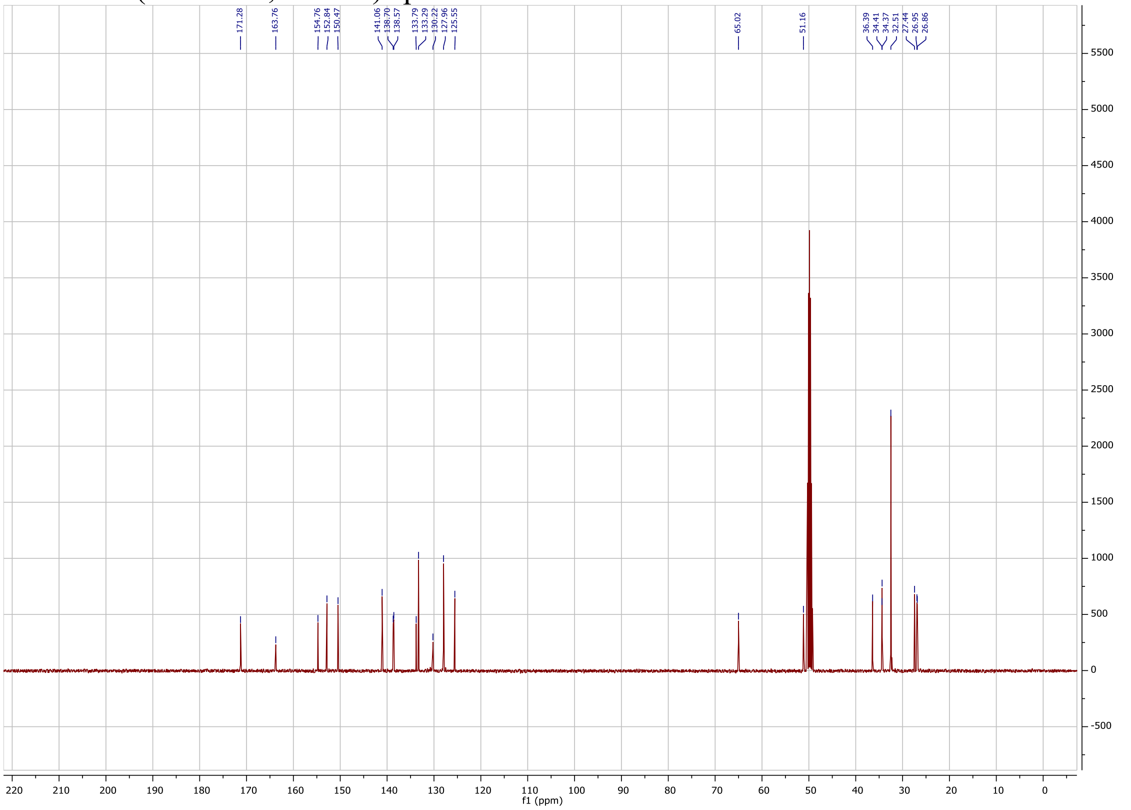

References

(1) Chen, Y.; Su, L.; Yang, X.; Pan, W.; Fang, H. Enantioselective Synthesis of 3,5-Disubstituted Thiohydantoins and Hydantoins. *Tetrahedron.* 2015, 71, 9234–9239.

(2) Hamuro, Y.; Scialdone, M. A.; DeGrado, W. F. Resin-to-Resin Acyl- and Aminoacyl-Transfer Reactions Using Oxime Supports. *J. Am. Chem. Soc.* 1999, 121, 1636–1644.
